# The evolutionary history of the *Arabidopsis lyrata *complex: a hybrid in the amphi-Beringian area closes a large distribution gap and builds up a genetic barrier

**DOI:** 10.1186/1471-2148-10-98

**Published:** 2010-04-08

**Authors:** Roswitha Schmickl, Marte H Jørgensen, Anne K Brysting, Marcus A Koch

**Affiliations:** 1 Heidelberg University, Heidelberg Institute of Plant Sciences, Biodiversity and Plant Systematics, Im Neuenheimer Feld 345, D-69120 Heidelberg, Germany; 2 Centre for Ecological and Evolutionary Synthesis (CEES), Department of Biology, University of Oslo, PO Box 1066 Blindern, NO-0316 Oslo, Norway

## Abstract

**Background:**

The genomes of higher plants are, on the majority, polyploid, and hybridisation is more frequent in plants than in animals. Both polyploidisation and hybridisation contribute to increased variability within species, and may transfer adaptations between species in a changing environment. Studying these aspects of evolution within a diversified species complex could help to clarify overall spatial and temporal patterns of plant speciation. The *Arabidopsis lyrata *complex, which is closely related to the model plant *Arabidopsis thaliana*, is a perennial, outcrossing, herbaceous species complex with a circumpolar distribution in the Northern Hemisphere as well as a disjunct Central European distribution in relictual habitats. This species complex comprises three species and four subspecies, mainly diploids but also several tetraploids, including one natural hybrid. The complex is ecologically, but not fully geographically, separated from members of the closely related species complex of *Arabidopsis halleri*, and the evolutionary histories of both species compexes have largely been influenced by Pleistocene climate oscillations.

**Results:**

Using DNA sequence data from the nuclear encoded cytosolic phosphoglucoisomerase and Internal Transcribed Spacers 1 and 2 of the ribosomal DNA, as well as the *trn*L/F region from the chloroplast genome, we unravelled the phylogeography of the various taxonomic units of the *A. lyrata *complex. We demonstrate the existence of two major gene pools in Central Europe and Northern America. These two major gene pools are constructed from different taxonomic units. We also confirmed that *A. kamchatica *is the allotetraploid hybrid between *A. lyrata *and *A. halleri*, occupying the amphi-Beringian area in Eastern Asia and Northern America. This species closes the large distribution gap of the various other *A. lyrata *segregates. Furthermore, we revealed a threefold independent allopolyploid origin of this hybrid species in Japan, China, and Kamchatka.

**Conclusions:**

Unglaciated parts of the Eastern Austrian Alps and arctic Eurasia, including Beringia, served as major glacial refugia of the Eurasian *A. lyrata *lineage, whereas *A. halleri *and its various subspecies probably survived in refuges in Central Europe and Eastern Asia with a large distribution gap in between. The North American *A. lyrata *lineage probably survived the glaciation in the southeast of North America. The dramatic climatic changes during glaciation and deglaciation cycles promoted not only secondary contact and formation of the allopolyploid hybrid *A. kamchatica*, but also provided the environment that allowed this species to fill a large geographic gap separating the two genetically different *A. lyrata *lineages from Eurasia and North America. With our example focusing on the evolutionary history of the *A. lyrata *species complex, we add substantial information to a broad evolutionary framework for future investigations within this emerging model system in molecular and evolutionary biology.

## Background

Molecular biological research during the last decade has largely focussed on model organisms such as *Drosophila melanogaster*, *Caenorhabditis elegans*, and *Arabidopsis thaliana*. Now that knowledge in molecular genetics, cell and developmental biology of these organisms has greatly increased, closely related organisms emerge as promising for studying characteristics not possible to elucidate with model and/or single organisms [1,2].

*Arabidopsis lyrata *L. is a close relative of *A. thaliana*, from which it diverged approximately five million years ago [3,4]. *Arabidopsis lyrata *s.l. represents a small species complex of four species and several putative subspecies with a circumpolar arctic-alpine distribution (Additional file 1, Table S1). Populations have been adapted to various ecological conditions, including the harsh environment of the arctic tundra, cryptic warm-stage refugia (exposed rocks, rocky slopes) in Central Europe, and different edaphic conditions with substrates such as dolomite, silicious bedrocks, and even heavy metal rich serpentine soil in Central Europe (Lower Austria, personal observation) and the USA [Maryland; [5]] [6]. Most members of the species complex are perennial diploid outbreeders (2*n *= 2*x *= 16), but also tetraploid cytotypes occur [7,6,8]. There are numerous aspects of the biology of the *A. lyrata *complex that differ from and cannot be addressed in *A. thaliana*, like self-incompatibility and perennial life cycle.

The *A. lyrata *complex has already proven to be a suitable study system for the analysis of character traits such as flowering time [9,10] or pathogen defense [11]. Additionally, molecular mechanisms for the function of sporophytic self-incompatibility have been investigated [12-20], and comparative approaches to analyse sporophytic self-incompatibility in diploids versus polyploids are underway (Jørgensen, unpublished data). Whole genome sequencing of *A. lyrata *was finished last year, and data have been available for a few months (The *A. lyrata *genome sequence assembly v1.0, http://genome.jgi-psf.org/Araly1/Araly1.info.html), enabling direct comparisons with the *A. thaliana *genome. However, in contrast to *A. thaliana*, where the evolutionary history has been analysed in more detail [e.g. [21,22]], evolutionary studies on the *A. lyrata *complex have so far been largely restricted to a small number of populations from Central Europe [23-26] or larger sample sizes with a more general genus-wide perspective [27,28]. A detailed European study has revealed that population structure is dominated by regional genetic bottlenecks, and genetic structure exists within continents [Ansell, personal communication]. This suggests a comprehensive global study is necessary to resolve the evolutionary history of this complex.

In this study we present the first worldwide evolutionary history of the *A. lyrata *complex, covering its whole range of distribution and all taxonomically defined units. We use a widely applied nuclear encoded marker system (ITS, internal transcribed spacer region of nuclear encoded ribosomal DNA) to study gene flow between populations, a maternally inherited chloroplast genome marker (*trn*L intron (*trn*L) and *trn*L/F intergenic spacer (*trn*L/F-IGS) of tRNA^Ser ^and tRNA^Thr^, respectively) to investigate migrational movements due to seed dispersal, and the nuclear encoded housekeeping gene *Pgi*C (cytosolic phosphoglucoisomerase), a single copy gene, to discriminate between hybridising taxa. We aim to focus on the following four aspects: (1) Unravelling general phylogeographic patterns of the *A. lyrata *complex by identifying the main genetic lineages, and interpreting genetic variation in space and time [29], in the context of both climatic and geological events throughout Pleistocene glaciation cycles; (2) Evaluating the role of hybridisation and polyploidisation in the origin of *A. kamchatica *(Fisch. ex DC.) K. Shimizu & Kudoh, an amphi-Beringian member of the *A. lyrata *complex; (3) Explaining Pleistocene and postglacial migration routes by analysing genetic diversity statistics: The arctic-alpine *A. lyrata *complex is one of the rare examples among higher plants with a distribution in both Central Europe and North America and, additionally, a circumpolar distribution - other examples are *Cassiope tetragona *[30], and *Saxifraga oppositifolia *[31]; and (4) Studying the role of Beringia as a refugia for populations of arctic *A. lyrata*, since Beringia is assumed to be one of the major refugia for arctic plants during Pleistocene glaciations [30-36].

## Methods

### Plant material

**Figure 1:**
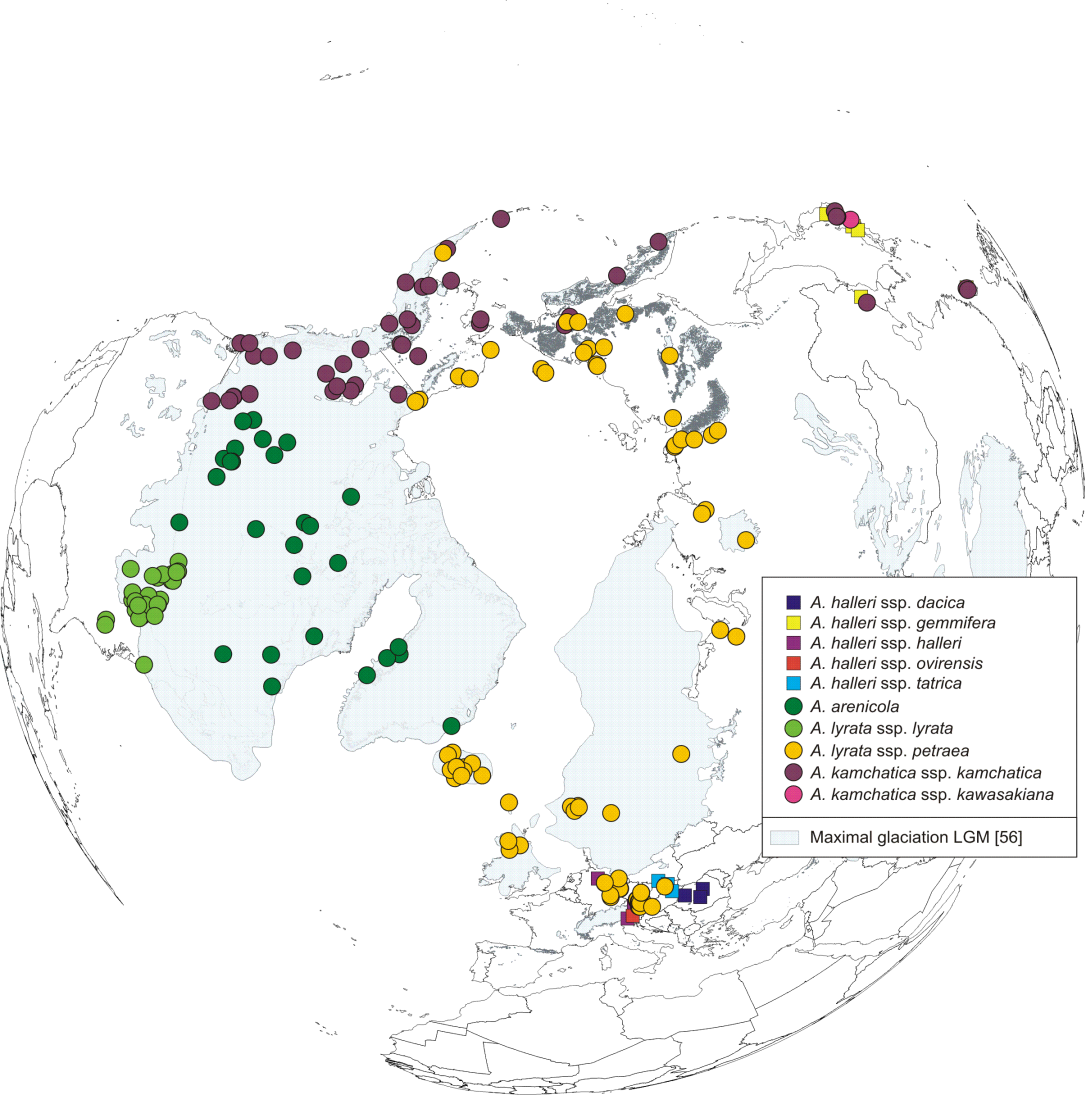
**Distribution of *Arabidopsis *accessions investigated**. Accessions of the *Arabidopsis lyrata *complex were analysed for nuclear ITS and *Pgi*C regions, and cpDNA *trn*L/F. Accessions of the *Arabidopsis halleri *complex were analysed for *Pgi*C only. Maximal glaciation of the LGM is drawn according to Ehlers and Gibbard [56].

Altogether 467 accessions of the *A. lyrata *complex were analysed: 295 accessions newly analysed within this study, 39 accessions sequenced by Schmickl et al. [28], and 133 accessions analysed by Koch and Matschinger [27]. Plant material was mainly collected from herbarium vouchers from BM (Natural History Museum, London), CAS (California Academy of Sciences, San Fransisco), DAO (Vascular Plant Herbarium, Agriculture and Agri-Food Canada, Ottawa), DH (Hobert and William Smith Colleges, New York), LE (The V.L. Komarov Botanical Institute, Russian Academy of Sciences, St. Petersburg), LI (Upper Austrian Provincial Museum, Linz), O (Natural History Museum, University of Oslo, Oslo), W (Natural History Museum, Vienna), and partly collected in the field, documented at HEID (Herbarium University of Heidelberg, Heidelberg). Taxon determination followed the voucher labels, and was verified with floras and determination keys [e.g. [37]; Flora of North America, Al-Shehbaz, personal communication]. In our study we followed the taxonomy of [37]. Twentyfive accessions of the various subspecies of *A. halleri *(L.) O'Kane & Al-Shehbaz were analysed from throughout the distribution range because of the evidence that this taxon served as a putative parent of *A. kamchatica*, previously also treated as *A. lyrata *ssp. *kamchatica *[[27]; and references therein], an evolutionary scenario which was recently confirmed by [8]. The distribution of the investigated accessions is shown in Figure 1. The accession list is provided with Additional file 2, Table S2.

The following short overview of *A. lyrata *and *A. halleri *taxonomy will introduce the concept of Al-Shehbaz and O'Kane [37]. Elven (ed.) [38] persues a different taxonomic concept, which is summarised in the supplementary material (Additional file 1, Table S1), but will not be discussed here. Apart from *A. thaliana*, the *A. lyrata *complex is one of three major species complexes in the genus *Arabidopsis *[27], the other two being the *A. arenosa *(L.) Lawalrée and *A. halleri *complexes [39,6,27,41]. The *A. lyrata *complex is considered by different authors to include different numbers of taxa of various ranking, distribution areas, and ploidy levels (Additional file 1, Table S1). Al-Shehbaz and O'Kane [37] treat the complex as one species, *A. lyrata*, with three subspecies: ssp. *lyrata*, ssp. *petraea *(L.) O'Kane & Al-Shehbaz, and ssp. *kamchatica *(Fisch. ex DC.) O'Kane & Al-Shehbaz. Subspecies *lyrata *is considered to be broadly amphi-Pacific and of two ploidy levels (2*n *= 16, 32), ssp. *petraea *to be Northern Eurasian and Central European with the same two ploidy levels, and *kamchatica *to be amphi-Pacific and tetraploid (2*n *= 32). A comprehensive quantitative morphological analysis using multivariate statistics is still not available for the *A. lyrata *complex. Moreover, information on the ploidy level is largely lacking throughout the distribution range, and further cytological investigations might be important for taxon delimitation [7,8]. *Arabidopsis arenicola *(Richardson) Al-Shehbaz, Elven, D.F. Murray & Warwick has only recently been included as part of the *A. lyrata *complex [42]. It has, for a long time, been placed within the genus *Arabis *L. Elven (ed.) [38] considers this taxon to be diploid (2*n *= 16) and distributed in north-eastern North America, and they are supported by Al-Shehbaz in the upcoming Flora of North America [Al-Shehbaz, personal communication]. Many of the mentioned taxa include two ploidy levels, suggesting frequent polyploidisation events within the *A. lyrata *complex. An allopolyploid origin of *kamchatica *has already been confirmed based on nuclear DNA sequences, with *A. lyrata *and *A. halleri *ssp. *gemmifera *(Matsum.) O'Kane & Al-Shehbaz as possible parental taxa [[43,27,28,8]; Jørgensen et al., unpublished data]. Otherwise, little is known with regard to the number of polyploid units and their origins.

Five subspecies have been recognised in *A. halleri*: ssp. *halleri*, ssp. *ovirensis *(Wulfen) O'Kane & Al-Shehbaz, ssp. *dacica *(Heuff.) Kolník, comb. nov., ssp. *tatrica *(Pawl.) Kolník, comb. nov., all distributed in Central Europe, and ssp. *gemmifera *in Eastern Asia, supported by both morphometric analysis (Kolnik, unpublished data) and genetic AFLP data (Marhold, unpublished data).

### DNA isolation, amplification and sequencing

Total DNA was obtained from dried leaf material and extracted according to the CTAB protocol of Doyle and Doyle [44] with the following modifications: 50-75 mg of dry leaf tissue were ground in 2 ml tubes using a Retsch swing mill (MM 200), 2 units of RNase A per extraction were added to the isolation buffer, and the DNA pellets were washed twice with 70% ethanol. DNA was dissolved in 50 μl TE-buffer for storage and diluted 1:3 in TE-buffer before use.

For the cpDNA markers *trn*L intron and *trn*L/F intergenic spacer (*trn*L/F-IGS), primers and PCR cycling scheme followed the protocol of Dobeš et al. [45], using a PTC200 (MJ Research, Waltham, USA) thermal cycler. The PCR reaction volume of 50 μl contained 1× PCR buffer (10 mM TRIS/50 mM KCl buffer, pH 8.0), 3 mM MgCl_2_, 0.4 μM of each primer, 0.2 mM of each dNTP, 1 U Taq DNA polymerase (Amersham Biosciences, Chalfont St Giles, England), and approximately 1 ng of template DNA. Amplified sequences of *trn*L/F-IGS included the complete *trn*L/F-IGS and the first 18 bases of the *trn*F gene. Amplification of the nuclear marker internal transcribed spacer region (ITS) was performed according to Dobeš et al. [46]. PCR reaction conditions were the same as for the two cpDNA markers described above, and PCR cycling scheme was 5 min at 95°C, 35 cycles of 1 min at 95°C, 1 min at 48°C, and 1 min at 72°C, 10 min extension at 72°C, and a final hold at 4°C. PCR products spanned the entire ITS1, 5.8 S rDNA, and ITS2 region.

Before sequencing PCR products were checked for length and concentrations on 1.5% agarose gels and purified with the NucleoFast Kit (Macherey-Nagel, Düren, Germany). Cycle sequencing was performed using the DYEnamic ET Terminator Cycle Sequencing Kit (Amersham Biosciences, Chalfont St Giles, England) and the original primers. Both strands were amplified in order to gain the complete sequence. PCR products were resolved in 10 μl loading solution and run on a MegaBace 500 sequencer.

#### trnL/F and ITS sequence definition and map reconstruction

Plastidic *trn*L/F sequences were defined as haplotypes and suprahaplotypes following our previous studies [27,41,28]: Haplotypes are characterised by multiple *trn*F pseudogenes in the 3'-region of the *trn*L/F-IGS close to the functional *trn*F gene [47,27,41,50]. When defining *trn*L/F suprahaplotypes, we excluded the pseudogene-rich region. Pseudogenes evolve with a 10 times higher mutation rate than single nucleotide polymorphisms, which makes them non-applicable for phylogenetic reconstructions on the species level [50]. Additionally, their boundaries are under ongoing discussion. An alternative interpretation is provided by Ansell et al. [51]. In summary, haplotypes belonging to one suprahaplotype share the same base order throughout the whole sequence except for the pseudogene-rich region, where they vary in both length and base content. Suprahaplotypes differ from each other only by single point mutations and/or indels. Newly defined *trn*L/F haplotypes were assigned to GenBank numbers [GQ922894-GQ922903] (Additional file 2, Table S2).

ITS sequences were obtained from direct sequencing of PCR products in order to detect hybrid individuals according to ambiguous base positions. Sequences were defined as ITS types and supratypes following our previous studies [27,41,28]: Most ITS types contained ambiguous sites as a result of the direct sequencing approach. They were combined to ITS supratypes by replacing the ambiguous sites by the bases with higher fluorescence intensity in the electropherogramm. Sequences with equal fluorescence intensity of the two bases at the ambiguous positions were only found between ITS supratypes b and e and labelled b/e ambiguous. Koch and Matschinger [27] already showed that analyses with either ITS types or ITS supratypes produce the same results. However, working with a limited number of ITS supratypes in contrast to a vast number of ITS types contributes to a clearer data display. Multiple ITS types within a single individual indicate the natural variability among ITS loci within an individual as result of either mutations or gene flow between individuals. In these individuals concerted evolution, which denotes the process of DNA sequence homogenisation among members of multigene families by gene conversion and/or unequal crossing over [52-55], is not yet completed. Newly defined ITS types were assigned to GenBank numbers [GQ922904-GQ922910] (Additional file 2, Table S2).

Maps were constructed using BioOffice version 2.0.6 to create shapefiles and drawn with ArcView version 8.2. Shapefiles for visualising the maximum extent of the ice sheets during the LGM (Last Glacial Maximum) were provided by Ehlers and Gibbard [56].

### Network analyses and genetic diversity statistics

Network analyses and genetic diversity statistics were exclusively performed using the *trn*L/F suprahaplotypes, as the pseudogene-rich region is not applicable for phylogenetic reconstructions on the species level [50]. The network was constructed using TCS version 1.21 [57] using the statistical parsimony algorithm [58]. Gaps (except polyT stretches) were coded as single additional binary characters. Newly characterised suprahaplotypes were added to the *trn*L/F network of the *A. lyrata *complex published earlier [28]. Genetic diversity statistics were performed with Arlequin version 3.11 [59]. Pairwise genetic differentiation was calculated among the following nine taxonomic and regional groups: *A. lyrata *ssp. *petraea *from (1) unglaciated Central Europe, (2) glaciated northern Europe, (3) northern Russia and western Beringia, (4) eastern Beringia; *A. lyrata *ssp. *lyrata *from (5) unglaciated North America and the glaciated Great Lakes region; (6) *Arabidopsis arenicola *from glaciated North America and Greenland; and *A. kamchatica *from (7) Japan, (8) north-eastern Russia, (9) Alaska and western Canada. F_ST _values, regarding haplotype frequencies only, and Φ_ST _values, which take into account the genetic relationship between haplotypes as pairwise character differences, were calculated. The significance of differentiation was examined using a permutation test with 1000 permutations. Additionally, genetic diversity was estimated as nucleotide diversity π.

### ITS parsimony analysis

ITS data were analysed based on a 652 bp alignment of ITS supratypes [27]. The total number of variable sites was 44, with 16 of them parsimony informative. From altogether 10 supratypes of both the *A. lyrata *complex and *A. halleri *ssp. *gemmifera*, a strict consensus tree was constructed using maximum parsimony with MEGA version 4.1 [60]. Heuristic searches were performed with 10 random addition sequences and Closest Neighbour Interchange (CNI) branch swapping. Bootstrap values were calculated based on 500 replicates. Length of the most parsimonious trees was 48 mutational steps with a consistency index (CI) = 0.89 and a retention index (RI) = 0.96 (autapomorphies excluded). *Arabidopsis thaliana *was used as outgroup.

### Primer design for the nuclear marker *PgiC*

Various higher plants are known to have a duplicated locus of the cytosolic enzyme phosphoglucoisomerase [61-63], and the loci are normally unlinked. Also within the genus *Arabidopsis*, extensive sequencing of the *Pgi*C locus using a cloning strategy revealed a duplication, both in the *A. lyrata *and *A. halleri *complex, and this duplication must have predated the evolutionary split between these two species complexes (Additional file 3, Figure S1; Jørgensen, unpublished data). Both loci were initially simultaneously amplified with the general forward primer 5'-TGCTGTSAGCACTAATCTTGCG-3' and the general reverse primer 5'-TCGAACCCGGGAGAGGTAGACCA-3', following the protocol of Wright et al. [23]. The resulting sequence data showed that a group of alleles at the *Pgi*C1 locus were exclusively found in *A. halleri *and the allopolyploid *A. kamchatica*. It was thus possible to design *A. halleri*-specific primers that worked as a high-throughput and simple PCR-based screening marker to discriminate between genomes of the *A. lyrata *and *A. halleri *complex. Unfortunately, it was not possible to develop a PCR-based reciprocal marker system characterising alleles from the *A. lyrata *gene pool because of a lack of appropriate DNA sequence variation. In general, the alleles from both duplicated *Pgi*C loci were only weakly differentiated among and within species. However, within the *Pgi*C1 locus we found a deletion of 7 bp length in the *A. lyrata *complex compared to the *A. halleri *complex (Additional file 4, Figure S2). Both groups of alleles were also substantially differentiated by various SNPs. A primer pair with the forward primer located partly within this 7 bp indel (5'-CATTCAACAGATTGTG-3') and the reverse primer 5'-CCAGTAAACATCATGT-3' was developed to amplify a 92 bp fragment within the *Pgi*C1 locus (Additional file 5, Figure S3). The PCR reaction volume of 50 μl contained 1× PCR buffer (10 mM TRIS/50 mM KCl buffer, pH 8.0), 2.5 mM MgCl_2_, 0.13 μM of each primer, 0.2 mM of each dNTP, 1 U Taq DNA polymerase (Amersham Biosciences, Chalfont St Giles, England), and approximately 1 ng of template DNA. The PCR cycling scheme was 3 min at 94°C, 35 cycles of 20 sec at 94°C, 30 sec at 56°C, and 20 sec at 68°C, 20 sec extension at 68°C, and a final hold at 4°C. By screening the absence/presence of the PCR product of this taxon-specific primer pair, we were able to follow the genetic footprint of the *A. halleri *complex in its allopolyploid hybrids throughout its distribution range.

## Results

### Chloroplast sequence data indicate three main genetic lineages: Eurasia, North America, and the amphi-Pacific region

**Figure 2:**
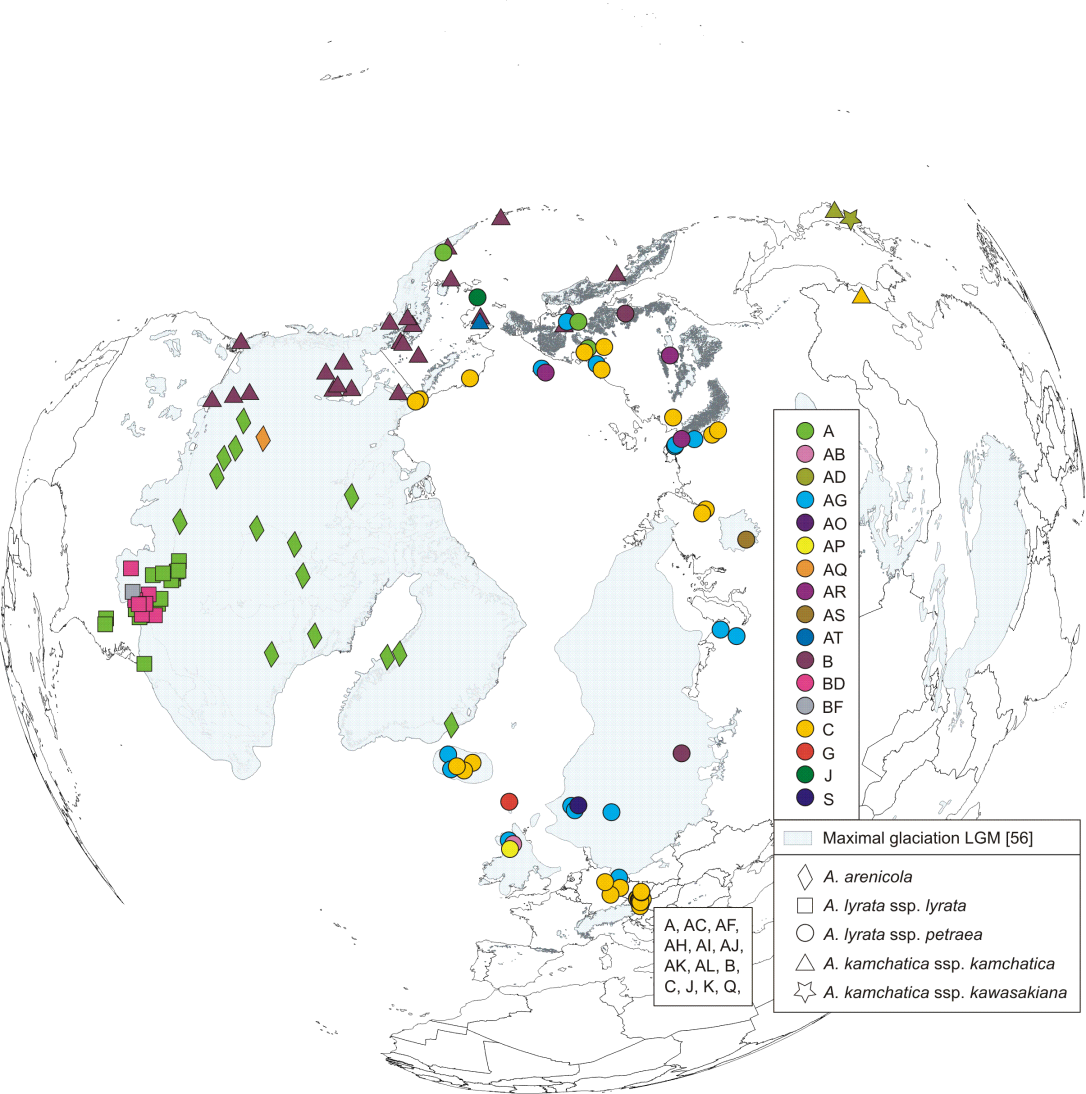
**Distribution of cpDNA *trn*L/F suprahaplotypes in the *Arabidopsis lyrata *complex**. Data newly presented in this study were combined with previous results from Koch and Matschinger [27] and Schmickl et al. [28]. *Trn*L/F suprahaplotypes were characterised as *trn*L intron and *trn*LF-IGS excluding the pseudogene-rich region in the *trn*LF-IGS. Accessions from Austria are listed separately. Maximal glaciation of the LGM is drawn according to Ehlers and Gibbard [56].

**Figure 3:**
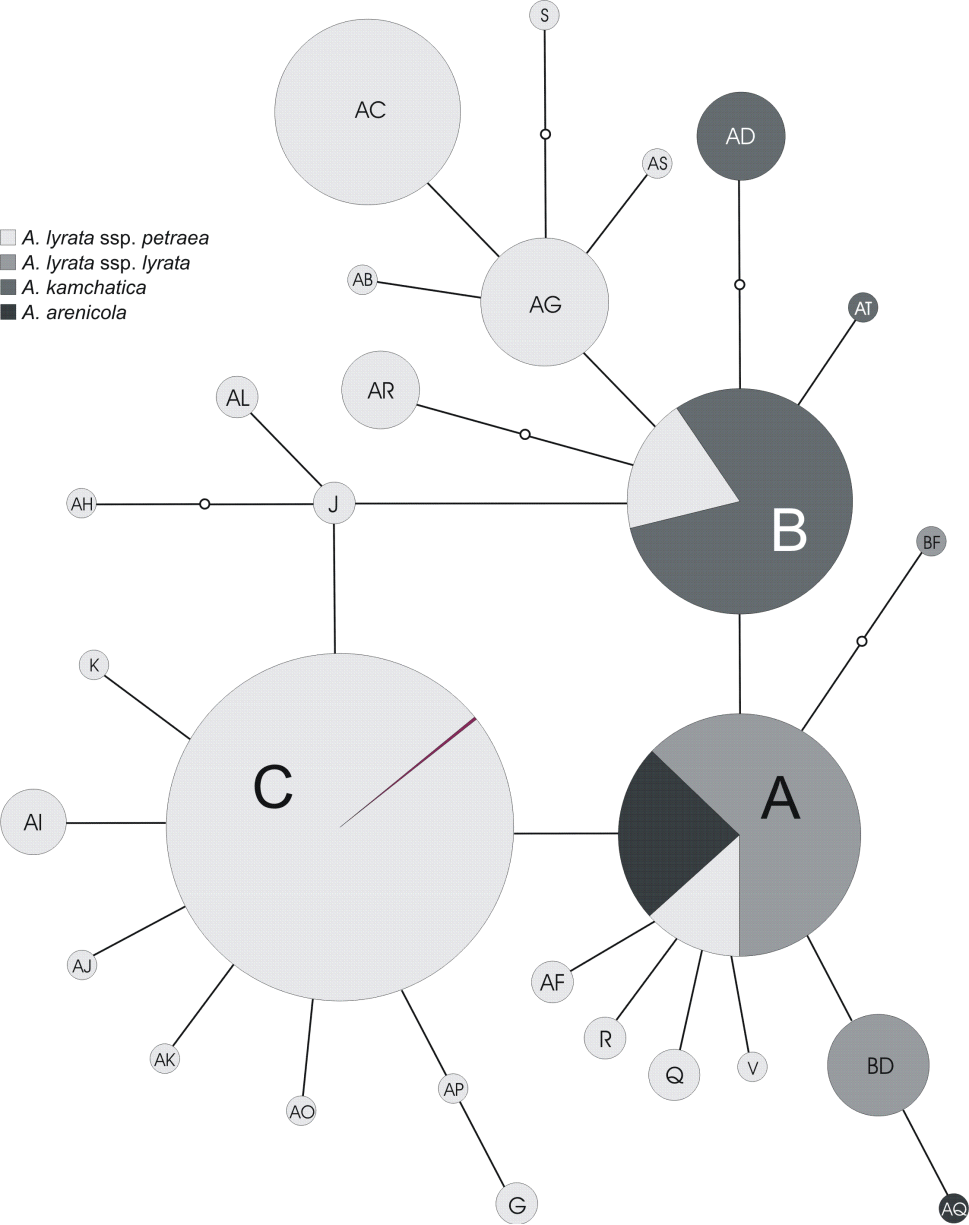
**CpDNA *trn*L/F suprahaplotype network of the *Arabidopsis lyrata *complex**. Data newly presented in this study were combined with previous results from Koch and Matschinger [27] and Schmickl et al. [28]. *Trn*L/F suprahaplotypes were characterised as *trn*L intron and *trn*LF-IGS excluding the pseudogene-rich region in the *trn*LF-IGS. The sizes of the circles indicate the relative frequency of a suprahaplotype in the whole dataset.

The investigated accessions (Figure 1), spanning the whole distribution range of the *A. lyrata *complex, were grouped into three genetic and geographically separated lineages: *trn*L/F suprahaplotype C in Eurasia, A in North America, and B in the amphi-Pacific region (Figure 2). The Eurasian lineage with suprahaplotype C had the largest distribution range: unglaciated Central Europe, formerly glaciated northern Europe, arctic Russia, Beringia, and Alaska (north of Brooks Range). The North American lineage, characterised by suprahaplotype A, included the United States (mainly around the Great Lakes), northeastern and central Canada (to the Canadian Rocky Mountains in the west), and Greenland. The third and amphi-Pacific lineage spanned from Kamchatka via Beringia into western Canada (with the Canadian Rocky Mountains as eastern border). These three suprahaplotypes were central in the suprahaplotype network (Figure 3), and all were connected to derived and less widely distributed suprahaplotypes: In the Eurasian lineage AC was widespread and found in Central Europe, mainly Austria, AG predominantly in the north (Iceland, Scandinavia, Russia), and AR in western Beringia (incl. Wrangel Island) (Figure 2). Unique suprahaplotypes, occurring only once in the whole dataset, were observed in Scotland (AB, AS), Austria (AH, AI, AJ, AK, AL, K, R, V), Iceland (AO, AP), Faeroe Islands (G), and Sweden (S). The North American lineage was additionally characterised by the unique suprahaplotypes AQ and BD, and the more widespread BF. Within the amphi-Pacific lineage, AD was detected exclusively in Japan.

Although all three lineages were characterised both by lineage-specific central and "tip" suprahaplotypes, sharing of central suprahaplotypes was observed, e.g. suprahaplotypes A and B were detected in a few accessions of the Eurasian lineage (Figure 2, Additional file 6, Table S3). This finding is congruent with the observation of central suprahaplotype sharing between the three main species complexes of the genus, *A. lyrata*, *A. halleri*, and *A. arenosa *[27]. This observation has been explained by ancestral cpDNA polymorphism predating the radiation of the genus approximately two million years ago [27].

### Cytosolic phosphoglucose isomerase identifies *Arabidopsis halleri *ssp. *gemmifera *as one parent of the allopolyploid amphi-Pacific *Arabidopsis kamchatica*

**Figure 4:**
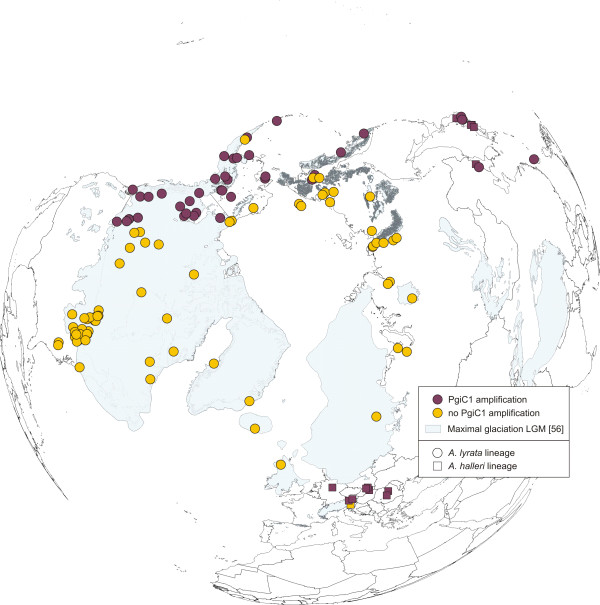
**Distribution of accessions with/without *Pgi*C1 amplification in the *Arabidopsis lyrata *complex and *A. halleri***. Amplification was successful in *Arabidopsis halleri *ssp. *gemmifera *from China and Japan, and in Central European subspecies of the *Arabidopsis halleri *complex (ssp. *dacica*, ssp. *halleri*, and ssp. *tatrica*). Amplification was also successful in all *A. kamchatica *accessions. Maximal glaciation of the LGM is drawn according to Ehlers and Gibbard [56].

The various *Pgi*C alleles detected were defined either as alleles at locus *Pgi*C1 or *Pgi*C2, and, therefore, considered as locus-specific (Additional file 3, Figure S1). However, taxon-specific lineage sorting of the various allele pools after the duplication event was not complete for *Pgi*C2. *A. kamchatica *carries *Pgi*C2 alleles hardly distinguishable from those of *A. septentrionalis *and *A. umbrosa*, which is an additional indicator that an Asian member of the *A. lyrata *complex served as one putative parental taxon. In addition, European *A. lyrata *ssp. *petraea *shares similar alleles with *A. halleri*, also indicating incomplete lineage sorting. The differentiation and lineage sorting of alleles at locus *Pgi*C1 is more taxon-specific. Here, *A. kamchatica *shares alleles most similar to those of *A. halleri *ssp. *gemmifera *from East Asia, and all of these alleles are significantly distinct from those of Eurasian *A. lyrata*. In summary, it is shown that *Pgi*C is not only a suitable marker to screen for hybrid speciation in *A. kamchatica*, but might also be a suitable marker to follow these alleles through space and time. Amplification of *Pgi*C1 alleles was successful in all *A. kamchatica *accessions (Figure 4, Additional file 5, Figure S3), but, as outlined above, failed in Eurasian and North American members of the *A. lyrata *complex (Figure 4, Additional file 5, Figure S3). *Pgi*C1 amplification was positive in all *A. halleri *ssp. *gemmifera *accessions and additionally in European subspecies of *A. halleri *(ssp. *dacica*, ssp. *halleri*, ssp. *tatrica*) (Figure 4, Additional file 5, Figure S3). Hence, presence of *Pgi*C1 alleles without deletion in the forward primer sequence was characteristic for the whole *A. halleri *species complex, except for *A. halleri *ssp. *ovirensis *(data not shown). In this subspecies either a secondary loss of this locus or a complementary mutation in the primer binding site might have occured. However, *A. halleri *ssp. *ovirensis *is a genetically distinct, local endemite at one single place in the southeastern Austrian Alps with an unclear evolutionary history [27,41].

### Gene diversity statistics show highest genetic diversity in the Eurasian lineage, strongly reduced diversity in the North American lineage, and extremely low diversity in the allopolyploid amphi-Pacific lineage

For genetic diversity statistics the distribution of the *A. lyrata *species complex was divided into nine different groups, according to the evidence for genetic lineages and Pleistocene history (Table 1). The Eurasian lineage (*A. lyrata *ssp. *petraea*) was split into four groups: unglaciated Central Europe, previously glaciated northern Europe or permafrost areas, northern Russia/western Beringia, and eastern Beringia. The North American lineage (*A. lyrata *ssp. *lyrata*) was separated into unglaciated North America/glaciated Great Lakes region and glaciated North America/Greenland. Amphi-Pacific *A. kamchatica *was differentiated into groups from Japan, Far Eastern Federal District of Russia, and Alaska/western Canada. The Eurasian lineage of *A. lyrata *ssp. *petraea *showed the highest cpDNA-based (*trn*L/F) nucleotide diversity (π), and it was equally high in unglaciated Central Europe (π = 0.0060), glaciated northern Europe (π = 0.0076), and northern Russia/western Beringia (π = 0.0077), but reduced in eastern Beringia (π = 0.0034) (Table 1). Pairwise Φ_ST _and F_ST _calculations showed only slight differentiation among unglaciated Central Europe, glaciated northern Europe, and northern Russia/western Beringia. For eastern Beringia the data were not statistically significant (*P *< 0.05; Table 2). These observations could indicate long-term survival of the *A. lyrata *complex in unglaciated Central Europe and northern Russia/western Beringia, or postglacial colonisation of formerly glaciated northern Europe from those two regions. The generally high nucleotide diversity in the Eurasian lineage was caused by a high number of unique and rare suprahaplotypes (Figure 2, Additional file 6, Table S3). In Central Europe the highest number of unique *trn*L/F suprahaplotypes (AC, AF, AH, AI, AJ, AK, AL, J, K, Q, R, V) was found in the foothills of the Eastern Austrian Alps, which remained unglaciated during Pleistocene climate oscillations. A high number of unique and rare suprahaplotypes was also found in formerly glaciated northern Europe (AB, AP - Scotland, AO - Iceland, G - Faeroe Islands, S - Norway). Subsequent geographic isolation of these populations during the Holocene warming might have caused restriction of suprahaplotypes to single geographic locations.

**Table 1:** Taxonomic and regional genetic differentiation based on cpDNA suprahaplotypes.

*Geographic region*	*n*	*π *× 10^-2^
*A. lyrata *ssp. *petraea*: Unglaciated Central Europe	196	0.603 +/- 0.329
*A. lyrata *ssp. *petraea*: Glaciated N Europe	21	0.764 +/- 0.425
*A. lyrata *ssp. *petraea*: N Russia, W Beringia	33	0.774 +/- 0.422
*A. lyrata *ssp. *petraea*: E Beringia	7	0.339 +/- 0.236
*A. lyrata *ssp. *lyrata*: Unglaciated N America, glaciated Great Lakes region	55	0.080 +/- 0.072
*A. arenicola*: Glaciated N America, Greenland	17	0.031 +/- 0.042
*A. kamchatica*: Japan	9	0.000 +/- 0.000
*A. kamchatica*: Far Eastern Federal District of Russia	9	0.000 +/- 0.000
*A. kamchatica*: Alaska, W Canada	38	0.007 +/- 0.019

Sample size (n) and nucleotide diversity (π) are provided with the corresponding standard deviation.

**Table 2:** Pairwise genetic differentiation (F_ST _and Φ_ST_) among taxonomic and regional groups.

Φ_ST_\F_ST_	*A. lyr*. ssp. *petraea*: Unglac. Central Europe	*A. lyr*. ssp. *petraea*: Glac. N Europe	*A. lyr*. ssp. *petraea*: N Russia, W Beringia	*A. lyr*. ssp. *petraea*: E Beringia	*A. lyr*. ssp. *lyrata*: Unglac. N America, glac. Great Lakes region	*A. arenicola*: Glac. N America, Greenland	*A. kamch*.: Japan	*A. kamch*.: Far Eastern Federal District of Russia	*A. kamch*.: Alaska, W Canada
*A. lyr*. ssp. *petraea*: Unglac. Central Europe		0.21832	0.17315	[-0.00029]	0.47816	0.52477	0.54797	0.53255	0.56147

*A. lyr*. ssp. *petraea*: Glac. N Europe	0.14828		[0.03253]	0.17947	0.44407	0.50004	0.48036	0.37743	0.53503

*A. lyr*. ssp. *petraea*: N Russia, W Beringia	0.19685	[-0.00620]		0.12632	0.43290	0.48110	0.49746	0.48437	0.60275

*A. lyr*. ssp. *petraea*: E Beringia	[-0.00716]	0.23663	0.26978		0.53564	0.71126	0.76892	0.76892	0.86207

*A. lyr*. ssp. *lyrata*: Unglac. N America, glac. Great Lakes region	0.37925	0.54668	0.49762	0.79114		[0.08456]	0.70822	0.70822	0.76248

*A. arenicola*: Glac. N America, Greenland	0.33441	0.40526	0.37345	0.77234	[0.02119]		0.92283	0.92283	0.92688

*A. kamch*.: Japan	0.59120	0.45048	0.40209	0.88490	0.93046	0.97806		1.00000	0.95735

*A. kamch*.: Far Eastern Federal District of Russia	0.49758	0.25161	0.19769	0.85450	0.90536	0.96954	1.00000		[-0.05564]

*A. kamch*.: Alaska, W Canada	0.54130	0.44301	0.33922	0.94213	0.93015	0.97891	0.97880	[-0.05564]	

F_ST _(above diagonal) and Φ_ST _(below diagonal) are both estimated from cpDNA suprahaplotypes. Values with *P *< 0.05 are given in brackets (permutation test with 1000 permutations).

In contrast to the Eurasian lineage of *A. lyrata *ssp. *petraea*, the North American lineage showed an approximately tenfold reduction in nucleotide diversity and strong differentiation according to pairwise Φ_ST _and F_ST _(Table 2). Nucleotide diversity of accessions from predominantly unglaciated southeastern North America (π = 0.0008) was higher than from North America and Greenland (π = 0.0003) (Table 1), which had been under the Laurentide ice sheet during the LGM, possibly indicating genetic bottlenecks with subsequent rapid postglacial immigration.

Extremely reduced nucleotide diversity was reported from amphi-Pacific *A. kamchatica *(π = 0.0000) (Table 1). When all three genetic groups of *A. kamchatica *were treated separately, only a single *trn*L/F suprahaplotype was found in each group (Japan: AD; China: C; Far Eastern Federal District of Russia/Alaska/western Canada: B) (Additional file 6, Table S3). Additionally, haplotype diversity was low, especially in *A. kamchatica *with suprahaplotype B. Only one haplotype (no. 84) was detected over a vast amphi-Pacific area from Kamchatka to western Canada (Additional file 7, Table S4).

### Refugia as areas of secondary contact of formerly allopatric populations: Beringia as an example

**Figure 5:**
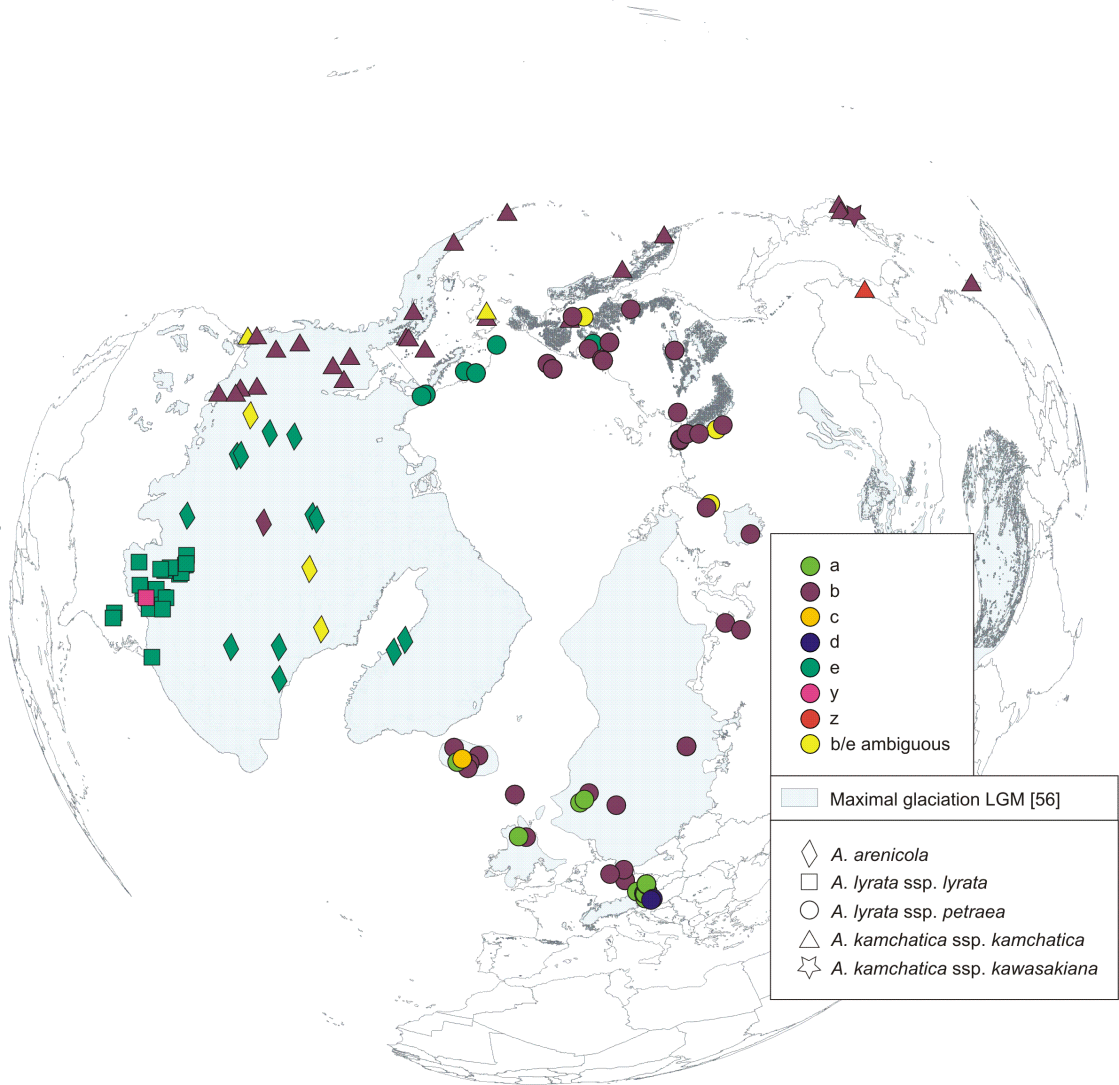
**Distribution of ITS supratypes in the *Arabidopsis lyrata *complex**. Data newly presented in this study were combined with previous results from Koch and Matschinger [27] and Schmickl et al. [28]. ITS supratypes were characterised as ITS1, 5.8 S rDNA, and ITS2 region, with ambiguous sites replaced by the bases with higher fluorescence intensity in the electropherogramm. Sequences with equal fluorescence intensity of the two bases at the ambiguous positions were only found between ITS supratypes b and e and labelled b/e ambiguous. The ITS supratypes are corresponding to those shown in Figure 6. Maximal glaciation of the LGM is drawn according to Ehlers and Gibbard [56].

**Figure 6:**
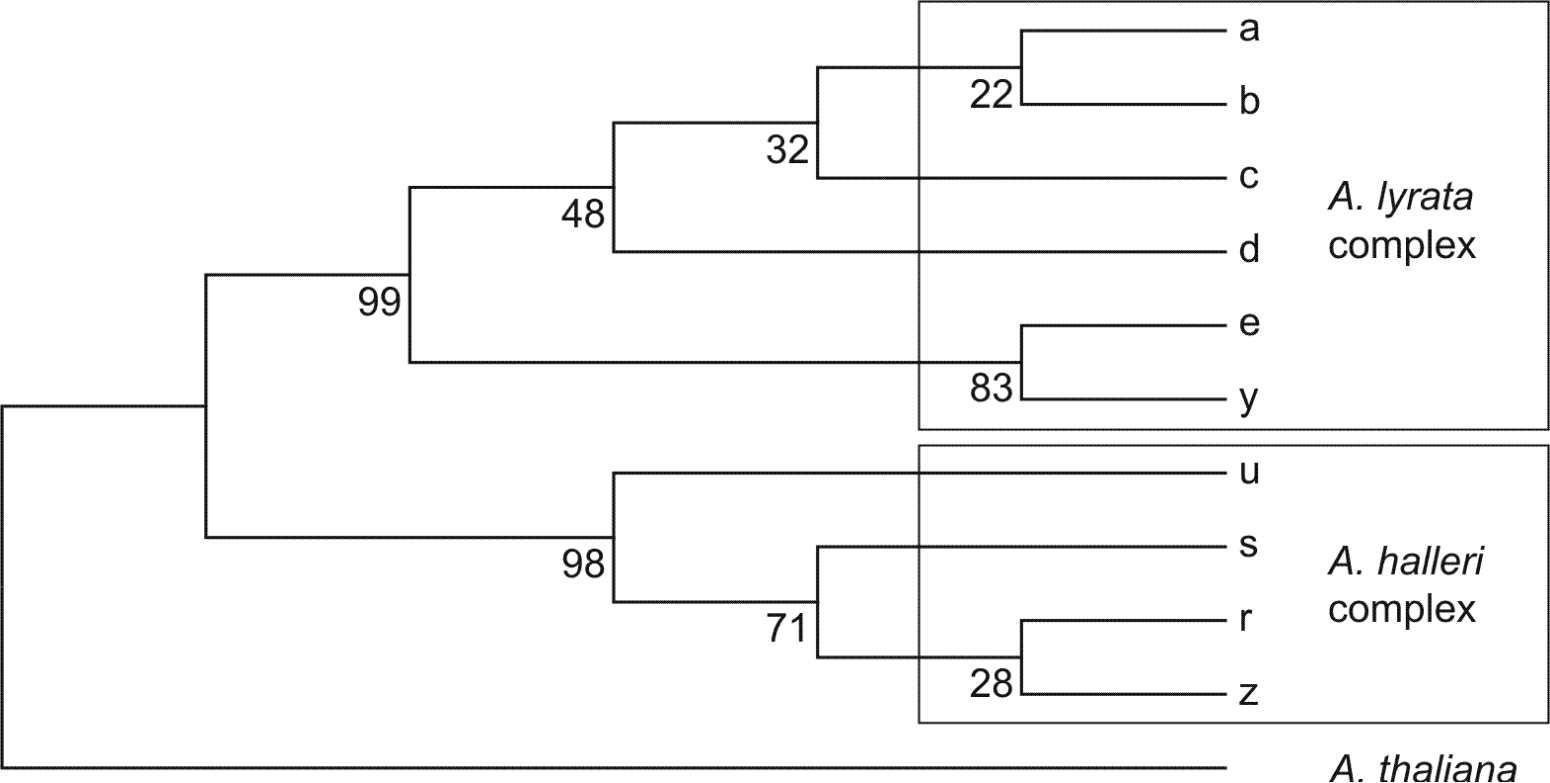
**Strict consensus tree from the maximum parsimony analysis of ITS supratypes in the *Arabidopsis lyrata*complex and *Arabidopsis halleri *ssp. *gemmifera***. From altogether 10 ITS supratypes (with ambiguous sites replaced by the bases with higher fluorescence intensity in the electropherogramm) of both the *A. lyrata *complex and *A. halleri *ssp. *gemmifera*, a strict consensus tree (length = 43) was constructed using maximum parsimony with MEGA version 4.1 [60]. Heuristic searches were performed with 10 random addition sequences and Closest Neighbour Interchange (CNI) branch swapping. Bootstrap values were calculated based on 500 replicates. Consistency index (CI) = 0.89, retention index (RI) = 0.96. *Arabidopsis thaliana *was used as outgroup.

Beringia, an arctic region ranging from Lena River in northeast Russia to Mackenzie River in Alaska and from the Arctic Ocean to mountains in southern Siberia and Alaska, is considered the major refugium for arctic taxa (reviewed by Abbott and Brochmann [64] and DeChain [65]), as it remained ice-free during Pleistocene climate oscillations. If we consider only the Eurasian and North American lineage of the *A. lyrata *complex, two major ITS groups met in Beringia (Figure 5): (1) the mainly Eurasian group carrying ITS supratype b (Figure 6), comprising Europe (with additional ITS supratypes a, c, d), northern Russia and western Beringia, and (2) the North American group carrying ITS supratypes e and, extremely rarely, y (Figure 6), including eastern and central North America, Greenland, and eastern Beringia (north of Brooks Range). The main contact zone was located in eastern Beringia, where accessions with *trn*L/F suprahaplotype C, characteristic for the Eurasian lineage, showed ITS supratype e, characteristic for the North American lineage. This is most likely due to ancient and/or recent gene flow from populations of the North American lineage into populations of the Eurasian lineage. Throughout the Beringian area ambiguous sites in ITS DNA sequences, caused by multiple ITS copies within a single genome and incomplete concerted evolution [54,28], were mainly found between ITS supratypes b (Eurasian lineage) and e (North American lineage). These results indicate that gene flow between these genetic groups may be counteracting the effects of concerted evolution. Interestingly, the allopolyploid amphi-Pacific lineage (*A. kamchatica*) also showed ITS supratype b like the Eurasian lineage.

## Discussion

### High genetic diversity of the *Arabidopsis lyrata *complex in Eurasia - postglacial migration from Central European and northern Russianrefugia

For arctic-alpine taxa, centres of species and genetic diversity are, in most cases, considered to concur with Pleistocene refugia [64,66,67]. In the genus *Arabidopsis*, both the number of accepted taxa, *trn*L/F suprahaplotype and ITS supratype diversity are highest in Central Europe, indicating this area as a centre of diversity [37,27]. Especially the Eastern Alps could have served as a refugium of *A. lyrata *ssp. *petraea*, since both diploid and cytogenetically stabilised tetraploid populations occur there (Schmickl and Koch, unpublished data). For the *A. lyrata *complex, however, Koch and Matschinger [27] concluded that Central Europe is not the only centre of diversity. Our results support this conclusion, showing that the Eurasian lineage of the *A. lyrata *complex is genetically diverse in both Central Europe and arctic Eurasia, including Beringia. To our knowledge such a pattern has not been observed for any other arctic plant with additional distribution in Central Europe. Either genetic diversity is high in the Arctic compared to the Alps, as in *Saxifraga oppositifolia *L., suggesting long-term evolution in Beringia and more recent colonisation of the Alps [33,31], or genetic diversity is highest in the Alps and decreasing towards the Arctic, as observed in *Arabis alpina *L. [68,69], indicating recent and rapid colonisation from Central Europe. Recent migration into the Arctic, associated with a loss of genetic diversity due to repeated bottlenecks, was also found in *Ranunculus glacialis *L. [70] and *Rubus chamaemorus *L. [71]. The overall high genetic diversity in the Eurasian *A. lyrata *lineage can probably be explained by long-term glacial survival in multiple refugia (Central Europe, northern Russia, western and eastern Beringia).

So far, periglacial survival of *A. lyrata *was reported only from unglaciated Central Europe, based on microsatellite data [25] and cpDNA sequences [27]. This study showed high genetic diversity also in formerly glaciated northern Europe, caused by numerous unique, locally distributed *trn*L/F suprahaplotypes. A possible explanation might be geographic isolation of populations, which either periglacially survived along the coastline or postglacially migrated into northern Europe, as reported from Ansell [personal communication]. Although several authors suggested periglacial survival on nunataks along the Norwegian coastline, mainly based on geomorphological investigations (reviewed by Brochmann et al. [34]), we assume postglacial colonisation of formerly glaciated northern Europe from both unglaciated Central Europe and northwestern Russia. Subsequent geographic isolation of populations during Holocene warming probably led to the fixation of local sequence types. Although in our study the major *trn*L/F suprahaplotypes C and AG were similarly frequent both in Central and northern Europe, a comparative microsatellite study between populations from Central Europe and Iceland revealed significant differences in marker polymorphism [25]. Riihimäki and Savolainen [9] even found divergent Central and northern European physiological morphotypes, with earlier and more frequent flowering plants in the south, and later and rarer flowering plants in the north.

We observed high genetic diversity within the Eurasian *A. lyrata *lineage, but also some genetic homogenisation. The *trn*L/F suprahaplotypes C and AG were found all over the distribution range, which could be explained by repeated gene flow between populations during glacial periods, additionally facilitated by rapid long-distance dispersal of the small *Arabidopsis *seeds across the smooth snow surface of the tundra and tundra-steppe by strong winds. This mode of long-distance dispersal could have bridged distances up to 2000 km [72] or even 4000 km (Westergaard et al., in prep.) and seems to be frequent in the Arctic [34,73].

### Ancient split of the Eurasian and North American lineage

The strong genetic differentiation we observed between the Eurasian and North American lineages is probably the result of long-term geographic isolation during Pleistocene glaciations. Muller et al. [26] detected genetic divergence between North American and western European populations in a comparative microsatellite study, and found that the former had a lower diversity. However, they focused on a few populations only and included only one North American population. The strong reduction of genetic diversity we observed in the North American lineage compared to the Eurasian lineage is congruent with nuclear and plastidic marker data of Wright et al. [23], but contradicting Balañα-Alcaide et al. [24], who reported similar genetic diversity based on two nuclear markers. However, both studies included only two populations from each of North America and Europe, and they may not have been representative. Because of the strongly reduced genetic diversity of the North American lineage, we assume this lineage to be derived from the Eurasian lineage. Future studies of more southern populations from the USA will test this assumption. Migration into North America apparently was associated with a strong genetic bottleneck, which restricted populations to ITS supratype e and *trn*L/F suprahaplotype A. Colonisation of North America is possible from different directions, either from Europe, or from East Asia. Both possibilities have been discussed for various circumboreal species (reviewed by Abbott and Brochmann [64]). In the case of *A. lyrata*, colonisation of North America from Russia seems, however, most likely, as the North American genotype was also rarely detected in western Beringia, but not at all in Iceland or Scandinavia.

### An amphi-Beringian *Arabidopsis *hybrid zone - due to allopolyploid success?

The majority of arctic polyploids have a history as postglacial colonisers [74]. It has frequently been assumed that polyploids have a broader adaptive potential for recolonising formerly glaciated areas [75,76]. As one of the major refugia for arctic plants, Beringia has a higher proportion of diploids compared to areas glaciated during Pleistocene [74]. New adaptations in polyploids may evolve either by genome rearrangements [77] and/or epigenetic changes [78-80] within the first generations after polyploid formation, as reported from rapid gene silencing in the allopolyploid *A. suecica *(Fr.) Norrl. ex O.E. Schulz [78]. Hybridisation is frequently involved in polyploidisation, leading to the formation of allopolyploids with one each of the parental genomes. Allopolyploidisations have been reported for several arctic species complexes, such as the high polyploid *Cerastium alpinum *L. complex [81], high polyploid *Primula *sect. *Aleuritia *[82-84], tetra-/hexaploid *Silene *L. [85,86], tetra- to octoploid *Saxifraga *section *Mesogyne *Sternb. [87,88], and tetra- to dodecaploid *Cardamine digitata *Richardson [89]. Introgression, the integration of genetic material from one species into another through repeated backcrossing, was observed between polyploid *Saxifraga cernua *L. and diploid *Saxifraga sibirica *L. [90]. The first known *Arabidopsis *allopolyploid was *A. suecica *with the maternal parent *A. thaliana *and the paternal parent *A. arenosa*, confirmed by artificial crosses [78]. This species probably developed around 20 000 years ago [91] or between 20 000 and 300 000 years ago [92], with a single origin in Fennoscandia [91,92]. The distribution range of this mainly outcrossing [93] allopolyploid species is rather small. The second natural *Arabidopsis *allopolyploid, *A. kamchatica*, has *A. halleri *ssp. *gemmifera *and a member of the *A. lyrata *complex as parental taxa [[43,27,8]; Jørgensen, unpublished data]. This allopolyploid origin could be confirmed not only for Japanese, but for all *A. kamchatica *accessions. According to chloroplast *trn*L/F data, three different genetic groups were found, which are geographically isolated from each other: (1) accessions of a widespread distribution range from Kamchatka, western and eastern Beringia, to pacific western Canada (*trn*L/F suprahaplotype B), (2) Japanese accessions (*trn*L/F suprahaplotype AD), and (3) accessions from pacific eastern China (*trn*L/F suprahaplotype C). In a more detailed comparison of chloroplast *trn*L/F and nuclear encoded ITS sequence data, two directions of gene flow could be observed: Either the paternal genome originated from a member of the *A. lyrata *species complex (ITS supratype b) and the maternal genome from *A. halleri *ssp. *gemmifera *(*trn*L/F suprahaplotype AD), as already reported for *A. kamchatica *from Japan [27]. Or *A. halleri *ssp. *gemmifera *represented the paternal genome (ITS supratype z, derived from ITS supratype r exclusively found in *A. halleri *ssp. *gemmifera*), and a member of the *A. lyrata *species complex served as donor of the maternal genome (*trn*L/F suprahaplotype C), as in *A. kamchatica *from China. The North American lineage of the *A. lyrata *complex could be excluded as a parent, as neither ITS supratype e nor *trn*L/F suprahaplotype A were detected in *A. kamchatica*. According to these data we suggest at least three independent origins of *A. kamchatica*, first with maternal *A. halleri *ssp. *gemmifera *in Japan, second with paternal *A. halleri *ssp. *gemmifera *in China, and third with an unknown direction of gene flow in Kamchatka, but in all cases with a member of the Eurasian *A. lyrata *lineage as hybridisation partner.

The most profound change in *A. kamchatica *in contrast to its parental species is the switch from outcrossing (with sporophytic self-incompatibility system) to selfing [ssp. *kamchatica*: [15,8]; ssp. *kawasakiana*: [94]]. Selfing increases the possibility of rapid range expansion, as a population can arise from a single individual independent of pollinators and pollen donors. Such a switch is already well known from *A. thaliana*, dated from around 413 000 [95] to one million years ago [96], and is therefore not necessarily correlated with hybridisation and polyploidisation. Change of mating systems is one of the major driving forces for speciation, initiating reproductive isolation of populations [97,98]. However, it is still unclear if there is a correlation between mating systems and hybridisation and/or polyploidisation. The breakdown of self-incompatibility in an artificial cross between *A. thaliana *and *A. lyrata *[99] could indicate a correlation between hybridisation and a switch in mating system, and it could further indicate possible dominance of the selfer *A. thaliana *over *A. lyrata*. Otherwise, breakdown of the sporophytic self-incompatibility system has been reported mainly from diploid individuals [16,100].

In contrast to *A. suecica*, allopolyploid *A. kamchatica *has a vast distribution range spanning the whole amphi-Beringian region. Our data suggest that this large distribution is partly due to postglacial colonisation of formerly glaciated areas in eastern Beringia and western Canada. The extremely reduced genetic diversity, particularly of the group with suprahaplotype B, suggests that postglacial immigration may have been rapid, possibly facilitated by de novo adaptations as a result of hybridisation and polyploidisation. Moreover, the success of *A. kamchatica *as a rapid coloniser may have been enhanced by the availability of large, open landscapes, where habitats were frequently disturbed by glacial and/or permafrost activity. However, the change in mating systems may have had a strong impact on the success of *A. kamchatica *as a postglacial coloniser, and also on the establishment of the genetic barrier between *A. kamchatica *and the Eurasian and North American lineages.

### Beringia as contact zone between the Eurasian and North American lineage of the *Arabidopsis lyrata *complex

Beringia served as a glacial refugium for numerous arctic plant taxa such as *Dryas integrifolia *Vahl. [32], *Saxifraga hirculus *L. [36], *Saxifraga oppositifolia *[33,31], and *Vaccinium uliginosum *L. [35,101]. Periglacial survival in Beringia can also be assumed for the *A. lyrata *complex, in particular, for the Eurasian lineage in arctic western and eastern Beringia north of Brooks Range. The nuclear sequence data indicate that gene exchange with populations of the North American lineage (*A. lyrata *ssp. *lyrata*, *A. arenicola*) occurred inter- and postglacially. However, the data do not support glacial survival of the North American lineage in Beringia, as no plastidic sequence types of this lineage were found in Beringia.

## Conclusions

By presenting a worldwide evolutionary history of the *Arabidopsis lyrata *species complex, we provide solid knowledge about centres of genetic diversity, different genetic lineages, their contact zones, and hybrid speciation. We could clearly differentiate three genetic lineages, a Eurasian, a North American, and an amphi-Pacific one. The latter is constituted of the allopolyploid A. kamchatica, a hybrid between *A. lyrata and A. halleri*. Further investigations of the population dynamics and the role of selfing within this hybrid species should be conducted to gain a deeper understanding of hybrid establishment in the wild.

## Authors' contributions

RS carried out the molecular marker studies and statistical analyses, constructed the maps and drafted the manuscript. MHJ constructed the *Pgi*C sequence alignment and helped to draft the manuscript. AKB contributed to draft the manuscript. MAK designed and coordinated the project and drafted the manuscript. All authors read and approved the final manuscript.

## Supplementary Material

Additional file 1 **Table S1**. The two main taxonomic concepts of the *Arabidopsis lyrata *complex. Summarised from Al-Shehbaz and O'Kane [37], including revision from the Flora of North America [Al-Shehbaz, personal communication], and Elven [38]. *Arabidopsis arenicola *was integrated from Warwick et al. [42]. Elven [38] excluded *Arabidopsis lyrata *from taxonomic treatment, as they assumed it to be a non-arctic, boreal taxon. Click here for file

Additional file 2 **Table S2**. Within this list all information about taxonomic unit, name on herbarium sheet, herbarium, herbarium number, locality, latitude/longitude, collector, collection date, accession number, ITS type, ITS GenBank number, ITS supratype, *trn*L intron type, *trn*L GenBank number, *trn*L/F-IGS type, *trn*L/F-IGS GenBank number, *trn*L intron + *trn*L/F-IGS type, *trn*L/F suprahaplotype, and *Pgi*C1 amplification is provided. Click here for file

Additional file 3 **Figure S1**. Single most parsimonious tree (length = 553) with bootstrap/jackknife values above 95, based on 37 *Arabidopsis *nuclear DNA *Pgi*C sequences. Heuristic searches were performed with 100 random addition sequences and TBR branch swapping, saving three trees per replicate, in TNT [102]. Gaps were treated as fifth state. Consistency index (CI) = 0.69, retention index (RI) = 0.95. Investigated accessions were from the *A. lyrata *complex (ssp. *lyrata*, ssp. *petraea*, *A. septentrionalis*, and *A. umbrosa*) and the *A. halleri *complex (ssp. *gemmifera*). Taxa with successful amplification of the chosen *Pgi*C1 fragment, and, consequently, without the deletion in the forward primer site, are marked with the blue box. Click here for file

Additional file 4 **Figure S2**. Alignment of the duplicated phosphoglucoisomerase loci *Pgi*C1 and *Pgi*C2 with the primer binding sites indicated. The forward primer is partly located within the 7 bp deletion between positions 1389 and 1396. Click here for file

Additional file 5 **Figure S3**. Selected PCR reactions from the *Pgi*C screening: No *Pgi*C1 amplification in members of the *Arabidopsis lyrata *complex (*A. lyrata *ssp. *lyrata*, *A. arenicola*, *Arabidopsis umbrosa*, and *A. septentrionalis*). Successful *Pgi*C1 amplification in members of *A. halleri *(ssp. *halleri*, ssp. *dacica*, ssp. *tatrica*, and ssp. *gemmifera*), and *A. kamchatica*. Click here for file

Additional file 6 **Table S3**. Taxonomic and regional genetic differentiation based on cpDNA suprahaplotypes. Numbers of cpDNA suprahaplotypes occurring in each region are provided. Click here for file

Additional file 7 **Table S4**. List of ITS supratypes, ITS types, *trn*L/F suprahaplotypes, and *trn*L/F haplotypes in the *Arabidopsis lyrata *complex with their corresponding frequencies of occurrence throughout the dataset *(italic)*. Click here for file
