# Supplementary material for: The evolutionary history of the Arabidopsis lyrata complex: a hybrid in the amphi-Beringian area closes a large distribution gap and builds up a genetic barrier
Source: BMC Evol Biol. 2010 Apr 8;10:98. doi: 10.1186/1471-2148-10-98 (PMC2858744; doi:10.1186/1471-2148-10-98)
Supplement: Additional file 1 — Table S1. The two main taxonomic concepts of the Arabidopsis lyrata complex. Summarised from Al-Shehbaz and O'Kane [37], including revision from the Flora of North America [Al-Shehbaz, personal communication], and Elven [38]. Arabidopsis arenicola was integrated from Warwick et al. [42]. Elven [38] excluded Arabidopsis lyrata from taxonomic treatment, as they assumed it to be a non-arctic, boreal taxon. [file 1471-2148-10-98-S1.DOC]

**Additional file 1 - Supplementary Information Table S1.**

The two main taxonomic concepts of the *Arabidopsis lyrata* complex.

| Taxonomy according to Al-Shehbaz and O´Kane [37] | | | |
| --- | --- | --- | --- |
| Species | Subspecies | Ploidy | Distribution range |
| *A. lyrata*  (L.) O'Kane &  Al-Shehbaz | ssp. *petraea*  (L.) O'Kane &  Al-Shehbaz | 2*n* = 16, 32 | Central Europe,  N Eurasia |
|  | ssp. *kamchatica*  (Fischer ex de Candolle) O'Kane & Al-Shehbaz | 2*n* = 32 | Amphi-Pacific |
|  | ssp. *lyrata* | 2*n* = 16, 32 | North America |
| *A. arenicola* (Richardson)  Al-Shehbaz, Elven, D.F.Murray & Warwick |  | 2*n* = 16 | NE North America |

| Taxonomy according to Elven [38], excluding *A. lyrata* from taxonomic treatment | | | |
| --- | --- | --- | --- |
| Species | Subspecies | Ploidy | Distribution range |
| *A. petraea* | ssp. *petraea* | 2*n* = 16 | Central and  N Europe |
|  | ssp. *septentrionalis*  (N.Busch) D.F.Murray & Elven | 2*n* = 16, 32 | Siberia |
|  | ssp. *umbrosa*  (Turcz. ex Steud.) D.F.Murray & Elven | 2*n* = 16 | Far Eastern Federal District, Russia, Alaska, Canada |
| *A. kamchatica* |  | 2*n* = 32 | Amphi-Pacific |
| *A. arenicola* (Richardson)  Al-Shehbaz, Elven, D.F.Murray & Warwick |  |  |  |

Summarised from Al-Shehbaz and O´Kane [37], including revision from the Flora of North America (Al-Shehbaz, personal communication), and Elven [38]. *Arabidopsis arenicola* was integrated from Warwick et al. [42]. Elven [38] excluded *Arabidopsis lyrata* from taxonomic treatment, as they assumed it to be a non-arctic, boreal taxon.
