# Supplementary material for: The evolutionary history of the Arabidopsis lyrata complex: a hybrid in the amphi-Beringian area closes a large distribution gap and builds up a genetic barrier
Source: BMC Evol Biol. 2010 Apr 8;10:98. doi: 10.1186/1471-2148-10-98 (PMC2858744; doi:10.1186/1471-2148-10-98)
Supplement: Additional file 2 — Table S2. Within this list all information about taxonomic unit, name on herbarium sheet, herbarium, herbarium number, locality, latitude/longitude, collector, collection date, accession number, ITS type, ITS GenBank number, ITS supratype, trnL intron type, trnL GenBank number, trnL/F-IGS type, trnL/F-IGS GenBank number, trnL intron + trnL/F-IGS type, trnL/F suprahaplotype, and PgiC1 amplification is provided. [file 1471-2148-10-98-S2.DOC]

**Additional file 2 - Supplementary Information Table S2.**

Within this list all information about taxonomic unit, name on herbarium sheet, herbarium, herbarium number, locality, latitude/longitude, collector, collection date, accession number, ITS type, ITS GenBank number, ITS supratype, *trn*L intron type, *trn*L GenBank number, *trn*L/F-IGS type, *trn*L/F-IGS GenBank number, *trn*L intron + *trn*L/F-IGS type, *trn*L/F suprahaplotype, and *Pgi*C1 amplification is provided.

| **Taxonomic**  **unit** | **Name on Herb. Sheet** | **Herb./**  **Herb. no.** | **Locality** | **Latitude/**  **Longitude** | **Collector/**  **Date** | **Acc.No** | **ITS**  **Type** | **ITS**  **GenBank no.** | **ITS**  **supra-**  **type** | ***trn*L**  **intron**  **type** | ***trn*L GenBank no.** | ***trn*L/F IGS type** | ***trn*L/F**  **IGS GenBank no.** | ***trn*L intron**  **+ *trn*L/F IGS type** | ***trn*L-*trn*L/F**  **supra-**  **haplo-**  **type** | ***Pgi*C1** |
| --- | --- | --- | --- | --- | --- | --- | --- | --- | --- | --- | --- | --- | --- | --- | --- | --- |
| arenicola | Arabis lyrata ssp. kamchatica | DAO  627787 | CAN; Alberta; ~25 miles N Fort McMurray, Mildred Lake | 57,1017  -111,4317 | M.G. Dumais & C. Hufnagel  11.06.1969 | Card0498 | 16 | DQ528878 | e | 22 | DQ313515 | 143 | EU418768 | 187 | AQ | no PgiC1 |
| arenicola | Arabis lyrata ssp. kamchatica | DAO  628274 | CAN; Alberta; Coronado, W Redwater on Highway 48 | 53,9503  -113,0842 | M.G. Dumais & K. Anderson  22.05.1968 | Card0553-02 |  |  |  | 2 | DQ313495 | 72 | DQ529068 | 31 | A |  |
| arenicola | Arabis lyrata ssp. kamchatica | DAO  628274 | CAN; Alberta; Coronado, W Redwater on Highway 48 | 53,9503  -113,0842 | M.G. Dumais & K. Anderson  22.05.1968 | Card0553-03 | 16 | DQ528878 | e |  |  |  |  |  |  | no PgiC1 |
| arenicola | Arabis lyrata ssp. kamchatica | DAO  628274 | CAN; Alberta; Coronado, W Redwater on Highway 48 | 53,9503  -113,0842 | M.G. Dumais & K. Anderson  22.05.1968 | Card0553-04 | 104 | EU418770 | e | 2 | DQ313495 | 72 | DQ529068 | 31 | A |  |
| arenicola | Arabis lyrata | DAO  564656 | CAN; Alberta; S Hondo | 55,0833  -114,0250 | E.H. Moss  27.06.1950 | Card0497 |  |  |  | 22 | DQ313515 |  |  |  |  | no PgiC1 |
| arenicola | Arabis arenicola | GH | CAN; Alberta; South shore of Lake Athabasca, Beaver Point, on lower open beach | 59,1269  -109,2822 | V.L. Harms, J.H. Hudson & R.A. Wright  09.07.1979 | Arab1798 |  |  |  | 2 | DQ313495 |  |  |  |  |  |
| arenicola | Arabis arenicola | O | CAN; Manitoba; Churchill, Churchill Northern Studies Centre | 58,7358  -93,8182 | S. Aiken & A. Brysting  22.07.2001 | OSLO-03 (01-010a) | 3 | DQ528816 | b | 2 | DQ313495 | 90 | DQ529086 | 50 | A | no PgiC1 |
| arenicola | Arabis arenicola | BM | CAN; Manitoba; Hudson Bay: Churchill, area above elevator | 48,7583  -94,0667 | Russell Marris  29.07.1970 | LON-09 | 16 | DQ528878 | e | 2 | DQ313495 | 90 | DQ529086 | 50 | A |  |
| arenicola | Arabis alpina | BM | CAN; Newfoundland; ~100 km inland from Nain, Fraser Canyon, at the head [west end] of Lake Tasisuak [Tessisoak Lake] | 56,6333  -62,6667 | G. Shepherd & B. Matthews  07.08.1973 | LON-26 | 16 | DQ528878 | e |  |  |  |  |  |  | no PgiC1 |
| arenicola | Arabis arenicola | O | CAN; Nunavut; Baffin Island: S Iqaluit, hills NE of settlement | 63,7500  -68,6500 | R. Elven  02.09.1999 | OSLO-01 (RE-3540/99) | 107 | GQ922906 | b | 2 | DQ313495 | 90 | DQ529086 | 50 | A | no PgiC1 |
| arenicola | Arabis arenicola | DAO  795214 | CAN; Nunavut; Franklin: Melville Peninsula, Committee Bay, Point 18 | 68,3197  -85,7794 | B.J. Woodruff  01.08.1948 | Arab1397 |  |  |  | 2 | DQ313495 |  |  |  |  |  |
| arenicola | Arabis arenicola | DAO  563514 | CAN; Nunavut; Keewatin: Chesterfield Inlet, SW edge of settlement | 63,3500  -90,7000 | D.B.O. Savile & C.T. Watts  10.07.1950 | Arab1395 |  |  |  | 2 | DQ313495 | 138 | DQ914838 | 181 | A |  |
| arenicola | Arabis arenicola | DAO  563542 | CAN; Nunavut; Keewatin: Southampton Island, Coral Harbour | 64,1500  -83,3000 | W.J. Cody  07.07.1948 | Arab1391 |  |  |  | 2 | DQ313495 |  |  |  |  |  |
| arenicola | Arabis arenicola | DAO  563538 | CAN; Nunavut; Keewatin: Southampton Island, Coral Harbour, beach at Munn Bay | 64,1333  -83,2833 | W.J. Cody  24.07.1948 | Arab1392 |  |  |  | 2 | DQ313495 |  |  |  |  |  |
| arenicola | Arabis arenicola | O | CAN; Nunavut; Southampton Island: Salliq [Coral Harbour], more recent graveyard near town on road to the airport | 64,1369  -83,1647 | S. Aiken & A. Brysting  30.07.2001 | OSLO-02 (01-081) | 103 | EU418769 | ambig. b/e | 2 | DQ313495 | 156 | FJ477703 | 208 | A | no PgiC1 |
| arenicola | Arabis arenicola | BM | CAN; Nunavut; Victoria Island: Cambridge Bay, Dease Strait | 69,0833  -105,1667 | Russell Marris  1974 | LON-08 |  |  |  | 2 | DQ313495 | 141 | EU418766 | 185 | A | no PgiC1 |
| arenicola | Arabis spec. | BM | CAN; Quebec; Ungava Bay: Fort Chimo | 58,1667  -68,3333 | Lord Tweedsmuir  09.-12.07.1961 | LON-22 | 16 | DQ528878 | e | 2 | DQ313495 | 90 | DQ529086 | 50 | A | no PgiC1 |
| arenicola | Arabis lyrata var. glabra | DAO  564644 | CAN; Saskatchewan; 3 miles S Hudson Bay Junction | 52,8567  -102,3850 | A.J. Breitung  01.06.1941 | Card0494 |  |  |  | 2 | DQ313495 |  |  |  |  | no PgiC1 |
| arenicola | Arabis lyrata var. glabra | DAO  564644 | CAN; Saskatchewan; 3 miles S Hudson Bay Junction | 52,8567  -102,3850 | A.J. Breitung  03.06.1941 | Card0494-02 |  |  |  | 2 | DQ313495 | 72 | DQ529068 | 31 | A |  |
| arenicola | Arabis lyrata | DAO  304225 | CAN; Saskatchewan; NE Meadow Lake, St. Cyr Sandhills | 54,2164  -108,0519 | W.G. Dore & K. Layton  26.06.1981 | Card0551-01 |  |  |  | 2 | DQ313495 | 90 | DQ529086 | 50 | A | no PgiC1 |
| arenicola | Arabis lyrata | DAO  304225 | CAN; Saskatchewan; NE Meadow Lake, St. Cyr Sandhills | 54,2164  -108,0519 | W.G. Dore & K. Layton  26.06.1981 | Card0551-02 |  |  |  | 2 | DQ313495 | 45 | DQ529042 | 188 | A | no PgiC1 |
| arenicola | Arabis lyrata ssp. kamchatica | DAO  564647 | CAN; Saskatchewan; opposite Prince Albert, N Saskatchewan River | 53,2000  -105,7500 | C. Frankton  15.05.1951 | Card0496 |  |  |  | 2 | DQ313495 | 72 | DQ529068 | 31 | A |  |
| arenicola | Arabis arenicola | DAO  03-290 | CAN | 52,4872  -71,4864 | Dignard & Desponts | Warwick19 | 16 | DQ165342 | e |  |  |  |  |  |  |  |
| arenicola | Arabis arenicola | DAO  268-62 | CAN | 54,3369  -105,6806 | Harms & Woo | Warwick20 | 16 | DQ165343 | e |  |  |  |  |  |  |  |
| arenicola | Arabis arenicola | DAO  33604 | CAN | 54,1619  -105,5950 | Argus | Warwick21 | 16 | DQ165344 | e |  |  |  |  |  |  |  |
| arenicola | Arabis arenicola | DAO  1260 | CAN | 64,4364  -96,1253 | Cody | Warwick22 | 16 | DQ165345 | e |  |  |  |  |  |  |  |
| arenicola | Arabis arenicola | DAO  1994 | CAN | 65,0947  -95,4767 | Cody | Warwick23 | 16 | DQ165346 | e |  |  |  |  |  |  |  |
| arenicola | Arabis arenicola | BM | DEN; Greenland; E-Greenland: Tunu, Blosseville Coast, east side of Korridoren glacier, extensively weathered basalt moraine | 68,7500  -26,5000 | A.J. Allan  09.08.1971 | LON-24 |  |  |  | 2 | DQ313495 | 90 | DQ529086 | 50 | A | no PgiC1 |
| arenicola | Arabis arenicola | DAO  563512 | DEN; Greenland; Holstenborg | 66,9367  -53,6694 | C.O. Erlanson  07.08.1927 | Arab1398 |  |  |  | 2 | DQ313495 |  |  |  |  |  |
| arenicola | Arabis arenicola | DAO  314914 | DEN; Greenland; Igdlorssuit | 71,2625  -52,8519 | C. Hansen, L. Kliim-Nielsen & B. Ollgaard  18.07.1967 | Arab1399 |  |  |  | 2 | DQ313495 | 139 | DQ914839 | 182 | A |  |
| arenicola | Arabis arenicola | BM | DEN; Greenland; W-Greenland: Kitaa, Disko, Nordfjord, Stordal | 69,9000  -54,2500 | L. Andersen & S. Hanfgarn  12.08.1975 | LON-10 | 16 | DQ528878 | e | 2 | DQ313495 | 90 | DQ529086 | 50 | A | no PgiC1 |
| arenicola | Arabis arenicola | BM | DEN; Greenland; W-Greenland: Kitaa, Svartenhuk Peninsula, Tartûssaq Hus | 71,6667  -55,0000 | M.P. Porsild  23.07.1935 | LON-18 | 16 | DQ528878 | e |  |  |  |  |  |  |  |
| arenicola | Arabis lyrata ssp. kamchatica | DAO  564580 | USA; Alaska; Cold Bay | 55,1858  -162,7211 | W.B. Schofield | Card0486 |  |  |  | 2 | DQ313495 | 88 | DQ529084 | 48 | A | no PgiC1 |
| kamchatica | Arabis lyrata | DAO  564663 | CAN; Alberta; Banff National Park: ~7 miles W Banff, along road to Sunshine Village (0.25 miles S Healy Creek Ranger Station) | 51,1767  -115,3900 | T. Mosquin & J.R. Seaborn  03.07.1968 | Card0470 | 3 | DQ528816 | b |  |  | 45 | DQ529042 |  |  | PgiC1 |
| kamchatica | Arabis lyrata ssp. kamchatica | DAO  564662 | CAN; Alberta; Banff National Park: Lake Agnes above Lake Louise | 51,4300  -116,1783 | J.A. Calder  27.07.1959 | Card0468 |  |  |  | 6 | DQ313499 | 45 | DQ529042 | 84 | B | PgiC1 |
| kamchatica | Arabis lyrata ssp. kamchatica | DAO  564662 | CAN; Alberta; Banff National Park: Lake Agnes above Lake Louise | 51,4300  -116,1783 | J.A. Calder  27.07.1959 | Card0468-01 | 3 | DQ528816 | b | 6 | DQ313499 | 45 | DQ529042 | 84 | B | PgiC1 |
| kamchatica | Arabis lyrata ssp. kamchatica | DAO  564662 | CAN; Alberta; Banff National Park: Lake Agnes above Lake Louise | 51,4300  -116,1783 | J.A. Calder  27.07.1959 | Card0468-02 | 3 | DQ528816 | b | 6 | DQ313499 | 45 | DQ529042 | 84 | B | PgiC1 |
| kamchatica | Arabis lyrata ssp. kamchatica | DAO  564661 | CAN; Alberta; Jasper National Park: Miette Hot Springs | 53,1767  -117,9233 | L. Jenkins  08.07.1955 | Card0469 | 3 | DQ528816 | b | 6 | DQ313499 | 45 | DQ529042 | 84 | B | PgiC1 |
| kamchatica | Arabis lyrata ssp. kamchatica | BM | CAN; British Columbia; Blue River, Cassiar Highway, at mile 21 from Alaska Highway | 59,7800  -129,1433 | J.A. Calder & J.M. Gillett  19.06.1960 | LON-13 | 3 | DQ528816 | b | 6 | DQ313499 | 45 | DQ529042 | 84 | B | PgiC1 |
| kamchatica | Arabis lyrata ssp. kamchatica | DAO  199571 | CAN; British Columbia; Ilgachuz Range [N Anahim Lake]: SE slope of Tundra Mountain, above Blue Canyon Creek | 52,7500  -125,2500 | R.L. Taylor and others  15.08.1972 | Card0484 |  |  |  | 4 | DQ313497 |  |  |  |  | PgiC1 |
| kamchatica | Arabis lyrata ssp. kamchatica | DAO  199571 | CAN; British Columbia; Ilgachuz Range [N Anahim Lake]: SE slope of Tundra Mountain, above Blue Canyon Creek | 52,7500  -125,2500 | R.L. Taylor and others  15.08.1972 | Card0484-04 | 3 | DQ528816 | b |  |  |  |  |  |  | PgiC1 |
| kamchatica | Arabis lyrata ssp. kamchatica | BM | CAN; British Columbia; Liard River Basin: Fairy Lake, slopes and cliffs at southwestern corner of lake | 48,5817  -124,3500 | G.W. Argus & E. Haber  27.07.1977 | LON-07 | 103 | EU418769 | ambig. b/e | 6 | DQ313499 | 45 | DQ529042 | 84 | B | PgiC1 |
| kamchatica | Arabis lyrata ssp. kamchatica | BM | CAN; British Columbia; Peace River Basin: Robb Lake, north side and west end of lake | 49,6167  -125,1500 | G.W. Argus & E. Haber  02.08.1977 | LON-16 | 3 | DQ528816 | b |  |  |  |  |  |  |  |
| kamchatica | Cardamine spec. | DAO  592745 | CAN; British Columbia; Ts´yl-os Provincial Park: Tchaikazan Valley, ridge of Carefree Mountains | 51,0617  -123,8667 | J. Pinder-Moss  07.08.1969 | Card0483 |  |  |  | 4 | DQ313497 |  |  |  |  | PgiC1 |
| kamchatica | Arabis lyrata ssp. kamchatica | DAO  564681 | CAN; British Columbia; Waterton Lakes National Park: Wall Lake near border to Alberta | 49,0131  -114,0956 | R.L. Taylor and others  03.08.1958 | Card0485 | 3 | DQ528816 | b | 6 | DQ313499 | 45 | DQ529042 | 84 | B | PgiC1 |
| kamchatica | Arabis lyrata v. kamtchatica Fisch. | DH  295541 | CAN; British Columbia; Yoho National Park: Kicking Horse Gorge | 51,4633  -116,2850 | W.C. McCalla  18.06.1942 | Card0265 |  |  |  | 6 | DQ313499 | 45 | DQ529042 | 84 | B | PgiC1 |
| kamchatica | Arabis lyrata ssp. kamchatica | DAO  345407 | CAN; Northwest Territories; Mackenzie District: Canol Road, mile 217 from Mackenzie River | 63,3500  -129,7167 | L. & G.P. Kershaw  25.06.1977 | Card0550 |  |  |  | 6 | DQ313499 | 45 | DQ529042 | 84 | B | PgiC1 |
| kamchatica | Arabis lyrata ssp. kamchatica | DAO  473434 | CAN; Yukon; Canol Road (Yukon Highway 6): kilometre 32.5 | 60,6000  -133,0500 | W.J. Cody  23.06.1980 | Card0471 | 3 | DQ528816 | b | 6 | DQ313499 | 45 | DQ529042 | 84 | B | PgiC1 |
| kamchatica | Arabis spec. | BM | CAN; Yukon; Little Hyland Valley, side valley | 61,7667  -128,5000 | Russell Marris  02.08.1972 | LON-23 | 3 | DQ528816 | b | 6 | DQ313499 | 45 | DQ529042 | 84 | B | PgiC1 |
| kamchatica | Arabis lyrata ssp. kamchatica | DAO  461276 | CAN; Yukon; Mackenzie Mountains: Macmillan Pass area, Hess River | 63,3167  -131,3500 | W.J. Cody  01.07.1981 | Card0472 |  |  |  |  |  | 45 | DQ529042 |  |  | PgiC1 |
| kamchatica | Arabis lyrata ssp. kamchatica | DAO  597305 | CAN; Yukon; Richardson Mountains: headwaters of Waters River | 67,6333  -137,2833 | W.J. Cody & J.H. Ginns  15.07.1982 | Card0473 |  |  |  | 6 | DQ313499 | 45 | DQ529042 | 84 | B | PgiC1 |
| kamchatica | Arabis lyrata L. v. glabra Hopkins | DH  587600 | CAN; Yukon; SW Yukon: vicinity of Mackintosh (Alaska Highway, mile 1022), Mount Archibald and along neighbouring stream | 60,7833  -137,8733 | W.B. Schefield & H.A. Crum  17.07.1957 | Card0264 |  |  |  |  |  | 45 | DQ529042 |  |  | PgiC1 |
| kamchatica | Arabis lyrata | DAO  159305 | CAN; | 61,7667  -127,1833 | S. Talbot  07.07.1976 | Card0475 |  |  |  | 6 | DQ313499 | 45 | DQ529042 | 84 | B | PgiC1 |
| kamchatica | Arabidopsis kamtchatica | HEID  501554 | JPN; Honshū; Foothills of Mount Fuji | 35,3658  138,8136 | J. Lihová, K. Marhold  10.07.2003 | JP83-01 | 3 | DQ528816 | b | 27 | DQ313519 | 75 | DQ529071 | 89 | AD | PgiC1 |
| kamchatica | Arabidopsis kamtchatica | HEID  501555 | JPN; Honshū; Foothills of Mount Fuji | 35,3658  138,8136 | J. Lihová, K. Marhold  10.07.2003 | JP83-02 | 3 | DQ528816 | b | 27 | DQ313519 | 75 | DQ529071 | 89 | AD | PgiC1 |
| kamchatica | Arabidopsis kamtchatica | HEID  501554 | JPN; Honshū; Foothills of Mount Fuji | 35,3658  138,8136 | J. Lihová, K. Marhold  10.07.2003 | JP83-03 | 3 | DQ528816 | b | 27 | DQ313519 | 75 | DQ529071 | 89 | AD | PgiC1 |
| kamchatica | Arabidopsis kamtchatica | HEID  501554 | JPN; Honshū; Foothills of Mount Fuji | 35,3658  138,8136 | J. Lihová, K. Marhold  10.07.2003 | JP83-04 | 3 | DQ528816 | b | 27 | DQ313519 | 75 | DQ529071 | 89 | AD | PgiC1 |
| kamchatica | Arabidopsis kamtchatica | HEID  501556 | JPN; Honshū; Foothills of Mount Fuji | 35,3658  138,8136 | J. Lihová, K. Marhold  10.07.2003 | JP83-05 |  |  |  | 27 | DQ313519 | 75 | DQ529071 | 89 | AD | PgiC1 |
| kamchatica | Arabidopsis kamtchatica | HEID  501556 | JPN; Honshū; Foothills of Mount Fuji | 35,3658  138,8136 | J. Lihová, K. Marhold  10.07.2003 | JP83-06 | 3 | DQ528816 | b | 27 | DQ313519 | 75 | DQ529071 | 89 | AD | PgiC1 |
| kamchatica | Arabidopsis kamtchatica | HEID  501557 | JPN; Honshū; Foothills of Mount Fuji | 35,3658  138,8136 | J. Lihová, K. Marhold  10.07.2003 | JP83-07 | 3 | DQ528816 | b | 27 | DQ313519 | 75 | DQ529071 | 89 | AD | PgiC1 |
| kamchatica | Arabidopsis kamtchatica | HEID  501557 | JPN; Honshū; Foothills of Mount Fuji | 35,3658  138,8136 | J. Lihová, K. Marhold  10.07.2003 | JP83-08 | 3 | DQ528816 | b | 27 | DQ313519 | 75 | DQ529071 | 89 | AD | PgiC1 |
| kamchatica | Arabidopsis kamtchatica | HEID  501557 | JPN; Honshū; Foothills of Mount Fuji | 35,3658  138,8136 | J. Lihová, K. Marhold  10.07.2003 | JP83-09 | 3 | DQ528816 | b |  |  | 75 | DQ529071 |  |  | PgiC1 |
| kamchatica | Arabis lyrata L. v. kamtchatica Fisch. | CAS  1020212 | JPN; Nagano; Kamiina: Hase Village, Mount Shiraiwa-dake | 35,7900  138,0967 | H. Kato, S. Katoh, M. Ho & M. Wakabayashi  27.06.2001 | Card0277 |  |  |  | 8 | DQ313501 |  |  |  |  | PgiC1 |
| kamchatica | Arabis lyrata ssp. kamchatica | BM | JPN; Shinano; Suwa-gun, Kamanashi-yama, Dôgasawa | 35,8500  138,1833 | Takasi Yamazaki  14.06.1968 | LON-11 | 3 | DQ528816 | b |  |  |  |  |  |  | PgiC1 |
| kamchatica | Arabis lyrata ssp. kamchatica | BM | ROC; Taiwan; Miao-li Hsien: Taian Hsiang, Kuanwu, guesthouse of the Taiwan Forestry Bureau | 24,4000  121,0833 | Ching-I Peng, T.S. Hsu, C.C. Wang, W.P. Leu & Mincho Peng  02.04.1992 | LON-17 | 3 | DQ528816 | b |  |  |  |  |  |  |  |
| kamchatica |  |  | ROC; SE Jilin, Changbai Mountains, Mount Changbai | 42,0097  128,0556 |  | 917275-01 | 113 | GU647161 | z | 2 | DQ313495 | 186 | GQ922902 | 253 | C | PgiC1 |
| kamchatica | Arabis lyrata L. ssp. kamtchatica (Fisch. ex DC.) Hult. | CAS  945388 | ROC; T´ai-chung Hsien, Ho-p´ing Hsinag, km 49 along route 7 between Sung-mao and Huan-shan | 24,5000  121,3333 | B. Bartholomen, Y.C. Kao & S.H. Lai  28.11.1997 | Card0278 |  |  |  | 8 | DQ313501 |  |  |  |  | PgiC1 |
| kamchatica | Arabis kamtchatica | O | RUS; Chukotka; Chukchi Peninsula: Yanrakinot, Bukhta Penkigney | 64,8673  172,6683 | H. Solstad & R. Elven  31.07.2005 | OSLO-05 (05/0457) | 109 | GQ922908 | e | 6 | DQ313499 | 45 | DQ529042 | 84 | B | PgiC1 |
| kamchatica | Arabis kamtchatica | LE | RUS; Chukotka; Gora Gilmimliney | 65,9083  173,6830 | P.G. Zhukova  12.07.1972 | OSLO-49 (72-144a) | 3 | DQ528816 | b | 6 | DQ313499 | 45 | DQ529042 | 84 | B | PgiC1 |
| kamchatica | Arabis kamtchatica | LE | RUS; Chukotka; Gora Gilmimliney | 65,9083  173,6830 | P.G. Zhukova  12.07.1972 | OSLO-53 (72-144b) |  |  |  | 6 | DQ313499 | 45 | DQ529042 | 84 | B | PgiC1 |
| kamchatica | Arabis kamtchatica | LE | RUS; Kamtchatka; Avachinskaya Sopka [Avachinsky/Avacha volcano] | 53,2550  158,8355 | 12.09.1973 | OSLO-52 (SH-W73-99) | 3 | DQ528816 | b | 6 | DQ313499 |  |  |  |  | PgiC1 |
| kamchatica | Arabidopsis kamchatica | O | RUS; Kamtchatka; Karaginski | 58,9500  164,2333 | Eriksen & Andersson  01.08.2005 | 1593-02 | 3 | DQ528816 | b | 6 | DQ313499 | 45 | DQ529042 | 84 | B | PgiC1 |
| kamchatica | Arabidopsis kamchatica | O | RUS; Kamtchatka; Karaginski | 58,9500  164,2333 | Eriksen & Andersson  01.08.2005 | 1593-05 | 3 | DQ528816 | b | 6 | DQ313499 | 45 | DQ529042 | 84 | B | PgiC1 |
| kamchatica | Arabidopsis kamchatica | O | RUS; Kamtchatka; Karaginski | 58,9500  164,2333 | Eriksen & Andersson  01.08.2005 | 1593-06 | 3 | DQ528816 | b | 6 | DQ313499 | 45 | DQ529042 | 84 | B | PgiC1 |
| kamchatica | Arabidopsis kamchatica | O | RUS; Kamtchatka; Karaginski | 58,9500  164,2333 | Eriksen & Andersson  01.08.2005 | 1593-08 | 3 | DQ528816 | b | 6 | DQ313499 | 45 | DQ529042 | 84 | B | PgiC1 |
| kamchatica | Arabidopsis kamchatica | O | RUS; Kamtchatka; Karaginski | 58,9500  164,2333 | Eriksen & Andersson  01.08.2005 | 1593-11 | 3 | DQ528816 | b |  |  | 45 | DQ529042 |  |  | PgiC1 |
| kamchatica | Arabidopsis kamchatica | O | RUS; Kamtchatka; Karaginski | 58,9500  164,2333 | Eriksen & Andersson  01.08.2005 | 1593-12 | 3 | DQ528816 | b |  |  | 45 | DQ529042 |  |  | PgiC1 |
| kamchatica | Arabidopsis kamchatica | O | RUS; Kamtchatka; Karaginski | 58,9500  164,2333 | Eriksen & Andersson  01.08.2005 | 1593-15 | 3 | DQ528816 | b |  |  | 45 | DQ529042 |  |  | PgiC1 |
| kamchatica | Arabidopsis kamchatica | O | RUS; Kamtchatka; Karaginski | 58,9500  164,2333 | Eriksen & Andersson  01.08.2005 | 1593-17 | 3 | DQ528816 | b | 6 | DQ313499 | 45 | DQ529042 | 84 | B |  |
| kamchatica | Arabidopsis kamchatica | O | RUS; Kamtchatka; Karaginski | 58,9500  164,2333 | Eriksen & Andersson  01.08.2005 | 1593-18 | 3 | DQ528816 | b |  |  | 45 | DQ529042 |  |  | PgiC1 |
| kamchatica | Arabidopsis kamchatica | O | RUS; Kamtchatka; Karaginski | 58,9500  164,2333 | Eriksen & Andersson  01.08.2005 | 1593-19 | 3 | DQ528816 | b |  |  | 45 | DQ529042 |  |  | PgiC1 |
| kamchatica | Arabidopsis kamchatica | O | RUS; Kamtchatka; Karaginski | 58,9500  164,2333 | Eriksen & Andersson  01.08.2005 | 1593-21 | 3 | DQ528816 | b |  |  | 45 | DQ529042 |  |  | PgiC1 |
| kamchatica | Arabidopsis kamchatica | O | RUS; Kamtchatka; Karaginski | 58,9500  164,2333 | Eriksen & Andersson  01.08.2005 | 1593-23 | 3 | DQ528816 | b |  |  |  |  |  |  | PgiC1 |
| kamchatica | Arabidopsis kamchatica | O | RUS; Kamtchatka; Karaginski | 58,9500  164,2333 | Eriksen & Andersson  01.08.2005 | OSLO-23  (1593) | 3 | DQ528816 | b | 6 | DQ313499 | 45 | DQ529042 | 84 | B | PgiC1 |
| kamchatica | Arabis lyrata ssp. kamchatica | BM | USA; Alaska; Alaska Range: Delta River, low wet area of 1 mile with high willows | 63,4333  -145,8000 | 20.06.1969 | LON-15 | 3 | DQ528816 | b | 6 | DQ313499 | 45 | DQ529042 | 84 | B | PgiC1 |
| kamchatica | Arabis lyrata v. kamtchatica Fisch. | CAS  338607 | USA; Alaska; Aleutian Islands: Attu Island, Bassett Creek, Sidden´s Valley | 52,8667  -173,2500 | R.M. Hardy  14.07.1945 | Card0269 | 3 | DQ528816 | b |  |  | 45 | DQ529042 |  |  | PgiC1 |
| kamchatica | Arabis lyrata ssp. kamchatica | DAO  564576 | USA; Alaska; Aleutian Islands: Attu Island, vicinity of Massacre Bay | 52,8533  -173,2167 | Lt. G.B. van Schaack  16.06.1945 | Card0481 |  |  |  | 6 | DQ313499 | 45 | DQ529042 | 84 | B | PgiC1 |
| kamchatica | Arabis lyrata ssp. kamchatica | DAO  564576 | USA; Alaska; Aleutian Islands: Attu Island, vicinity of Massacre Bay | 52,8533  -173,2167 | Lt. G.B. van Schaack  16.06.1945 | Card0481-02 |  |  |  | 6 | DQ313499 | 90 | DQ529086 | 191 | B | PgiC1 |
| kamchatica | Arabidopsis kamchatica | O | USA; Alaska; Anchorage | 61,2166  -149,8996 | H. Solstad  01.09.2005 | OSLO-34 (BE05-1320-1) | 3 | DQ528816 | b | 6 | DQ313499 | 45 | DQ529042 | 84 | B | PgiC1 |
| kamchatica | Arabidopsis kamchatica | O | USA; Alaska; Anchorage | 61,2166  -149,8996 | H. Solstad  01.09.2005 | OSLO-35 (BE05-1320-2) | 3 | DQ528816 | b |  |  | 45 | DQ529042 |  |  | PgiC1 |
| kamchatica | Arabis lyrata ssp. kamchatica | DAO  564581 | USA; Alaska; Cold Bay | 55,1858  -162,7211 | W.B. Schofield  25.07.1952 | Card0480 | 3 | DQ528816 | b | 6 | DQ313499 | 45 | DQ529042 | 84 | B | PgiC1 |
| kamchatica | Arabidopsis kamchatica | O | USA; Alaska; Goodnews Bay: Goodnews Bay Village, airstrip | 59,1167  -161,5833 | C.L. Parker  11.06.2004 | OSLO-06 (15510) | 18 | DQ528880 | b | 6 | DQ313499 | 45 | DQ529042 | 84 | B | PgiC1 |
| kamchatica | Arabis lyrata L. ssp. occidentalis (Wats.) Piper | DH  499790 | USA; Alaska; Katmai National Monument: southern shore of Lake Naknek, E outlet of Brooks River | 58,5500  -155,7833 | R. Barrett  12.06.1962 | Card0275 |  |  |  |  |  | 45 | DQ529042 |  |  | PgiC1 |
| kamchatica | Arabis lyrata L. ssp. kamtchatica (Fisch.) Hult. | CAS  230345 | USA; Alaska; Kodiak Archipelago: Sitkalidak Island, Port Hobron | 57,1606  -153,1564 | W.J. Eyerdam  28.07.1931 | Card0270 |  |  |  |  |  | 45 | DQ529042 |  |  | PgiC1 |
| kamchatica | Arabis lyrata | DAO  598172 | USA; Alaska; Mount Hayes Quadrangle: south end of Rainbow Mountain | 63,2500  -145,5833 | C.L. Parker & D.F. Murray  22.08.1989 | Card0479 | 3 | DQ528816 | b | 6 | DQ313499 | 45 | DQ529042 | 84 | B | PgiC1 |
| kamchatica | Arabis lyrata | DAO  598172 | USA; Alaska; Mount Hayes Quadrangle: south end of Rainbow Mountain | 63,2500  -145,5833 | C.L. Parker & D.F. Murray  22.08.1989 | Card0479-02 | 3 | DQ528816 | b | 6 | DQ313499 | 45 | DQ529042 | 84 | B | PgiC1 |
| kamchatica | Arabis lyrata ssp. kamchatica | DAO  564579 | USA; Alaska; Naknek | 58,7283  -157,0139 | W.B. Schofield  17.08.1954 | Card0490 |  |  |  |  |  | 45 | DQ529042 |  |  | PgiC1 |
| kamchatica | Arabidopsis kamchatica | O | USA; Alaska; Nome | 64,5021  -165,4067 | C.L. Parker & H. Solstad  28.08.2005 | BE05-1269-01 |  |  |  | 6 | DQ313499 | 45 | DQ529042 | 84 | B | PgiC1 |
| kamchatica | Arabidopsis kamchatica | O | USA; Alaska; Nome | 64,5021  -165,4067 | C.L. Parker & H. Solstad  28.08.2005 | BE05-1269-04 | 105 | GQ922904 | e | 6 | DQ313499 | 45 | DQ529042 | 84 | B | PgiC1 |
| kamchatica | Arabidopsis kamchatica | O | USA; Alaska; Nome | 64,5021  -165,4067 | C.L. Parker & H. Solstad  28.08.2005 | BE05-1269-06 | 105 | GQ922904 | e | 6 | DQ313499 | 45 | DQ529042 | 84 | B | PgiC1 |
| kamchatica | Arabidopsis kamchatica | O | USA; Alaska; Nome | 64,5021  -165,4067 | C.L. Parker & H. Solstad  28.08.2005 | BE05-1269-07 | 105 | GQ922904 | e | 6 | DQ313499 | 45 | DQ529042 | 84 | B | PgiC1 |
| kamchatica | Arabidopsis kamchatica | O | USA; Alaska; Nome | 64,5021  -165,4067 | C.L. Parker & H. Solstad  28.08.2005 | BE05-1269-09 | 105 | GQ922904 | e | 6 | DQ313499 | 45 | DQ529042 | 84 | B | PgiC1 |
| kamchatica | Arabidopsis kamchatica | O | USA; Alaska; Nome | 64,5021  -165,4067 | C.L. Parker & H. Solstad  28.08.2005 | BE05-1269-11 | 3 | DQ528816 | b | 6 | DQ313499 | 45 | DQ529042 | 84 | B | PgiC1 |
| kamchatica | Arabidopsis kamchatica | O | USA; Alaska; Nome | 64,5021  -165,4067 | C.L. Parker & H. Solstad  28.08.2005 | BE05-1269-12 | 3 | DQ528816 | b | 6 | DQ313499 | 45 | DQ529042 | 84 | B | PgiC1 |
| kamchatica | Arabidopsis kamchatica | O | USA; Alaska; Nome | 64,5021  -165,4067 | C.L. Parker & H. Solstad  28.08.2005 | BE05-1269-14 | 3 | DQ528816 | b | 6 | DQ313499 |  |  |  |  | PgiC1 |
| kamchatica | Arabidopsis kamchatica | O | USA; Alaska; Nome | 64,5021  -165,4067 | C.L. Parker & H. Solstad  28.08.2005 | BE05-1269-15 |  |  |  | 6 | DQ313499 | 45 | DQ529042 | 84 | B | PgiC1 |
| kamchatica | Arabidopsis kamchatica | O | USA; Alaska; Nome | 64,5021  -165,4067 | C.L. Parker & H. Solstad  28.08.2005 | BE05-1269-18 | 3 | DQ528816 | b | 6 | DQ313499 | 45 | DQ529042 | 84 | B | PgiC1 |
| kamchatica | Arabidopsis kamchatica | O | USA; Alaska; Nome | 64,5021  -165,4067 | C.L. Parker & H. Solstad  28.08.2005 | BE05-1269-20 | 3 | DQ528816 | b | 6 | DQ313499 |  |  |  |  | PgiC1 |
| kamchatica | Arabidopsis kamchatica | O | USA; Alaska; Nome | 64,5021  -165,4067 | C.L. Parker & H. Solstad  28.08.2005 | BE05-1269-22 |  |  |  | 6 | DQ313499 | 45 | DQ529042 | 84 | B | PgiC1 |
| kamchatica | Arabidopsis kamchatica | O | USA; Alaska; Nome | 64,5021  -165,4067 | C.L. Parker & H. Solstad  28.08.2005 | OSLO-36  (BE05-1269-01) | 105 | GQ922904 | e | 6 | DQ313499 | 45 | DQ529042 | 84 | B | PgiC1 |
| kamchatica | Arabidopsis kamchatica | O | USA; Alaska; Nome | 64,5021  -165,4067 | C.L. Parker & H. Solstad  28.08.2005 | OSLO-37  (BE05-1269-02) | 103 | EU418769 | ambig. b/e | 6 | DQ313499 | 45 | DQ529042 | 84 | B | PgiC1 |
| kamchatica | Arabidopsis kamchatica | O | USA; Alaska; Nome | 64,5021  -165,4067 | C.L. Parker & H. Solstad  28.08.2005 | OSLO-38  (BE05-1269-03) | 105 | GQ922904 | e | 6 | DQ313499 | 45 | DQ529042 | 84 | B | PgiC1 |
| kamchatica | Arabis lyrata L. ssp. kamtchatica (Fisch.) Hult. | CAS  525565 | USA; Alaska; Prince William Sound, Sheppard Bay, Hawkins Island | 60,5000  -146,0833 | W.J. Eyerdam  1965 | Card0274 |  |  |  | 6 | DQ313499 | 45 | DQ529042 | 84 | B | PgiC1 |
| kamchatica | Arabidopsis kamchatica | O | USA; Alaska; Seward Peninsula: Kigluaik Mountains, W Star Creek, N Taylor Highway | 64,9167  -164,9833 | R. Elven  09.07.2002 | OSLO-04 (219302) | 108 | GQ922907 | e | 34 | FJ477683 | 157 | FJ477704 | 207 | AT | PgiC1 |
| kamchatica | Arabis lyrata ssp. kamchatica | DAO  564542 | USA; Alaska; Susitna Valley: Montana Creek Lodge | 62,0797  -150,0728 | Wm. W. Mitchell  02.07.1964 | Card0476 |  |  |  | 6 | DQ313499 | 45 | DQ529042 | 84 | B | PgiC1 |
| kamchatica | Arabis kamtchatica | O | USA; Alaska; White Mountains: Fossil Creek | 65,5314  -147,5434 | O. Gjærevoll  1954 | OSLO-07 (831) | 3 | DQ528816 | b | 6 | DQ313499 | 45 | DQ529042 | 84 | B | PgiC1 |
| kamchatica | Arabis lyrata | DAO  295466 | USA; Colorado; Maroon Creek Valley | 54,7667  -128,6167 | B.R. Foster  10.06.1977 | Card0482 | 3 | DQ528816 | b | 4 | DQ313497 |  |  |  |  | PgiC1 |
| kawasa-kiana | Arabidopsis kawasakiana |  | JPN | 34,5531  136,5969 |  | B2002-E2II-18-8 | 3 | DQ528816 | b | 27 | DQ313519 | 137 | DQ914837 | 256 | AD |  |
| lyrata |  |  | CAN; Ontario; Lake Erie: Long Point Provincial Park | 42,5800  -80,3900 | B. Mable | LPT-5 | 108 | GQ922907 | e | 42 | GQ922894 | 135 | DQ529009 | 242 | BD | no PgiC1 |
| lyrata |  |  | CAN; Ontario; Lake Erie: Long Point Provincial Park | 42,5800  -80,3900 | B. Mable | LPT-6 |  |  |  | 42 | GQ922894 | 135 | DQ529009 | 242 | BD | no PgiC1 |
| lyrata | Arabis lyrata | DAO  296874 | CAN; Ontario; Lake Erie: Long Point Provincial Park [Norfolk County], Squire´s Ridge | 42,5550  -80,2500 | A.A. Reznicek & P.M. Catling  25.05.1980 | Card0491 |  |  |  |  |  | 135 | DQ529009 |  |  | no PgiC1 |
| lyrata |  |  | CAN; Ontario; Lake Erie: Point Pelee Provincial Park | 41,9200  -82,5100 | B. Mable | PTP-3 | 108 | GQ922907 | e |  |  | 135 | DQ529009 |  | A | no PgiC1 |
| lyrata |  |  | CAN; Ontario; Lake Erie: Point Pelee Provincial Park | 41,9200  -82,5100 | B. Mable | PTP-4 |  |  |  | 42 | GQ922894 | 135 | DQ529009 | 242 | BD | no PgiC1 |
| lyrata |  |  | CAN; Ontario; Lake Erie: Rondeau Bay | 42,2600  -81,8500 | B. Mable | RON-1 | 110 | GQ922909 | y | 42 | GQ922894 | 135 | DQ529009 | 242 | BD | no PgiC1 |
| lyrata |  |  | CAN; Ontario; Lake Erie: Rondeau Bay | 42,2600  -81,8500 | B. Mable | RON-3 | 110 | GQ922909 | y | 42 | GQ922894 | 135 | DQ529009 | 242 | BD | no PgiC1 |
| lyrata |  |  | CAN; Ontario; Lake Huron: Georgian Bay, [Notta]Wasaga Bay | 44,5100  -80,0100 | B. Mable | WAS-1 |  |  |  | 42 | GQ922894 | 143 | EU418768 | 251 | BD | no PgiC1 |
| lyrata |  |  | CAN; Ontario; Lake Huron: Georgian Bay, [Notta]Wasaga Bay | 44,5100  -80,0100 | B. Mable | WAS-3 | 108 | GQ922907 | e | 42 | GQ922894 | 143 | EU418768 | 251 | BD | no PgiC1 |
| lyrata |  |  | CAN; Ontario; Lake Huron: Manitoulin Island | 45,6600  -82,2600 | B. Mable | MAN-5 |  |  |  | 2 | DQ313495 | 179 | GQ922895 | 245 | A | no PgiC1 |
| lyrata |  |  | CAN; Ontario; Lake Huron: Manitoulin Island | 45,6600  -82,2600 | B. Mable | MAN-6 |  |  |  |  |  | 179 | GQ922895 |  |  | no PgiC1 |
| lyrata |  |  | CAN; Ontario; Lake Huron: Pinery Provincial Park | 43,2700  -81,8300 | B. Mable | PIN-5 |  |  |  | 42 | GQ922894 | 135 | DQ529009 | 242 | BD | no PgiC1 |
| lyrata |  |  | CAN; Ontario; Lake Huron: Pinery Provincial Park | 43,2700  -81,8300 | B. Mable | PIN-9 |  |  |  | 42 | GQ922894 | 135 | DQ529009 | 242 | BD | no PgiC1 |
| lyrata |  |  | CAN; Ontario; Lake Superior: Lake Superior Provincial Park | 47,5700  -84,9700 | B. Mable | LSP-3 | 109 | GQ922908 | e | 2 | DQ313495 | 90 | DQ529086 | 50 | A | no PgiC1 |
| lyrata |  |  | CAN; Ontario; Lake Superior: Lake Superior Provincial Park | 47,5700  -84,9700 | B. Mable | LSP-4 |  |  |  | 2 | DQ313495 | 90 | DQ529086 | 50 | A |  |
| lyrata |  |  | CAN; Ontario; Lake Superior: Lake Superior Provincial Park, Old Woman Bay | 47,7900  -84,8900 | B. Mable | OWB-5 | 16 | DQ528878 | e | 2 | DQ313495 | 90 | DQ529086 | 50 | A | no PgiC1 |
| lyrata |  |  | CAN; Ontario; Lake Superior: Lake Superior Provincial Park, Old Woman Bay | 47,7900  -84,8900 | B. Mable | OWB-9 | 16 | DQ528878 | e | 2 | DQ313495 | 90 | DQ529086 | 50 | A | no PgiC1 |
| lyrata |  |  | CAN; Ontario; Lake Superior: Pukaskwa National Park | 48,4000  -86,1900 | B. Mable | PUK-8 |  |  |  | 2 | DQ313495 | 90 | DQ529086 | 50 | A | no PgiC1 |
| lyrata |  |  | CAN; Ontario; Lake Superior: Pukaskwa National Park | 48,4000  -86,1900 | B. Mable | PUK-9 | 16 | DQ528878 | e | 2 | DQ313495 | 90 | DQ529086 | 50 | A | no PgiC1 |
| lyrata | Arabis lyrata ssp. kamchatica | DAO  564622 | CAN; Ontario; SE corner of St. Ignace Island, SW corner of St. Ignace Harbour | 48,7500  -87,8017 | C.E. Garton  28.07.1958 | Card0492 | 16 | DQ528878 | e | 2 | DQ313495 | 89 | DQ529085 | 49 | A | no PgiC1 |
| lyrata | Arabis lyrata | DAO  603901 | CAN; Ontario; Thunder Bay District: Lake Superior (Loc. 687), Michipicoten Island, SW corner of island and eastwards | 47,7000  -85,9300 | J.H. Soper & F.A. Fraser  21.07.1964 | Card0493 |  |  |  | 2 | DQ313495 | 90 | DQ529086 | 50 | A | no PgiC1 |
| lyrata | Arabis lyrata | DAO  603901 | CAN; Ontario; Thunder Bay District: Lake Superior (Loc. 687), Michipicoten Island, SW corner of island and eastwards | 47,7000  -85,9300 | J.H. Soper & F.A. Fraser  21.07.1964 | Card0493-01 |  |  |  | 2 | DQ313495 | 90 | DQ529086 | 50 | A | no PgiC1 |
| lyrata | Arabis lyrata | DAO  603901 | CAN; Ontario; Thunder Bay District: Lake Superior (Loc. 687), Michipicoten Island, SW corner of island and eastwards | 47,7000  -85,9300 | J.H. Soper & F.A. Fraser  21.07.1964 | Card0493-02 |  |  |  | 2 | DQ313495 | 90 | DQ529086 | 50 | A | no PgiC1 |
| lyrata | Arabis lyrata | DAO  603901 | CAN; Ontario; Thunder Bay District: Lake Superior (Loc. 687), Michipicoten Island, SW corner of island and eastwards | 47,7000  -85,9300 | J.H. Soper & F.A. Fraser  21.07.1964 | Card0493-03 |  |  |  | 2 | DQ313495 | 72 | DQ529068 | 31 | A | no PgiC1 |
| lyrata |  |  | CAN; Ontario; Thunder Bay District: Pic River | 48,6000  -86,3000 | B. Mable | PIC-5 | 16 | DQ528878 | e | 2 | DQ313495 | 90 | DQ529086 | 50 | A | no PgiC1 |
| lyrata |  |  | CAN; Ontario; Thunder Bay District: Pic River | 48,6000  -86,3000 | B. Mable | PIC-7 | 16 | DQ528878 | e | 2 | DQ313495 | 90 | DQ529086 | 50 | A | no PgiC1 |
| lyrata |  |  | CAN; Ontario; Tobermory, Cliffs ("IN") | 45,2400  -81,5100 | B. Mable | TC(IN)-54 | 16 | DQ528878 | e | 2 | DQ313495 | 90 | DQ529086 | 50 | A | no PgiC1 |
| lyrata |  |  | CAN; Ontario; Tobermory, Cliffs ("IN") | 45,2400  -81,5100 | B. Mable | TC(IN)-56 | 16 | DQ528878 | e | 2 | DQ313495 | 90 | DQ529086 | 50 | A |  |
| lyrata |  |  | CAN; Ontario; Tobermory, Cliffs ("OUT") | 45,2500  -81,5300 | B. Mable | TC(OUT)-9 | 16 | DQ528878 | e | 2 | DQ313495 | 90 | DQ529086 | 50 | A | no PgiC1 |
| lyrata |  |  | CAN; Ontario; Tobermory, Cliffs ("OUT") | 45,2500  -81,5300 | B. Mable | TC(OUT)-10 | 16 | DQ528878 | e | 2 | DQ313495 | 90 | DQ529086 | 50 | A | no PgiC1 |
| lyrata |  |  | CAN; Ontario; Tobermory, Singing Sands Alvar | 45,1900  -81,5800 | B. Mable | TSSA-1 |  |  |  | 2 | DQ313495 | 90 | DQ529086 | 50 | A | no PgiC1 |
| lyrata |  |  | CAN; Ontario; Tobermory, Singing Sands Alvar | 45,1900  -81,5800 | B. Mable | TSSA-2 |  |  |  | 2 | DQ313495 | 90 | DQ529086 | 50 | A | no PgiC1 |
| lyrata |  |  | CAN; Ontario; Tobermory, Singing Sands Beach | 45,1900  -81,5900 | B. Mable | TSS-1 |  |  |  | 2 | DQ313495 | 90 | DQ529086 | 50 | A | no PgiC1 |
| lyrata |  |  | CAN; Ontario; Tobermory, Singing Sands Beach | 45,1900  -81,5900 | B. Mable | TSS-2 | 16 | DQ528878 | e | 2 | DQ313495 | 90 | DQ529086 | 50 | A | no PgiC1 |
| lyrata |  |  | USA; Indiana; Lake Michigan: Indiana Dunes National Lakeshore | 41,6475  -87,0711 | B. Mable | IND-1 | 16 | DQ528878 | e | 2 | DQ313495 | 179 | GQ922895 | 245 | A | no PgiC1 |
| lyrata |  |  | USA; Indiana; Lake Michigan: Indiana Dunes National Lakeshore | 41,6475  -87,0711 | B. Mable | IND-4 | 16 | DQ528878 | e | 42 | GQ922894 | 135 | DQ529009 | 242 | BD | no PgiC1 |
| lyrata |  |  | USA; Michigan; Lake Huron: Port Crescent State Park | 44,0000  -83,0700 | B. Mable | PCR-1 | 108 | GQ922907 | e | 42 | GQ922894 | 135 | DQ529009 | 242 | BD | no PgiC1 |
| lyrata |  |  | USA; Michigan; Lake Huron: Port Crescent State Park | 44,0000  -83,0700 | B. Mable | PCR-2 |  |  |  | 42 | GQ922894 | 135 | DQ529009 | 242 | BD | no PgiC1 |
| lyrata |  |  | USA; Michigan; Lake Michigan: Beaver Island | 45,6469  -85,5522 | B. Mable | BEI-1 | 109 | GQ922908 | e | 2 | DQ313495 |  |  |  |  | no PgiC1 |
| lyrata |  |  | USA; Michigan; Lake Michigan: Beaver Island | 45,6469  -85,5522 | B. Mable | BEI-2 | 109 | GQ922908 | e | 2 | DQ313495 |  |  |  |  | no PgiC1 |
| lyrata |  |  | USA; Michigan; Lake Michigan: Sleeping Bear Dunes National Lakeshore | 44,9400  -85,8700 | B. Mable | SBD-1 |  |  |  | 2 | DQ313495 | 90 | DQ529086 | 50 | A | no PgiC1 |
| lyrata |  |  | USA; Michigan; Lake Michigan: Sleeping Bear Dunes National Lakeshore | 44,9400  -85,8700 | B. Mable | SBD-3 | 16 | DQ528878 | e | 2 | DQ313495 | 182 | GQ922898 | 249 | A | no PgiC1 |
| lyrata |  |  | USA; Michigan; Lake Superior: Pictured Rocks National Lakeshore | 46,3900  -86,0100 | B. Mable | PIR-2 | 16 | DQ528878 | e | 2 | DQ313495 | 179 | GQ922895 | 245 | A | no PgiC1 |
| lyrata |  |  | USA; Michigan; Lake Superior: Pictured Rocks National Lakeshore | 46,3900  -86,0100 | B. Mable | PIR-3 | 16 | DQ528878 | e | 2 | DQ313495 | 179 | GQ922895 | 245 | A | no PgiC1 |
| lyrata |  |  | USA; North Carolina; Mayodan | 36,6833  -80,6167 | D. Remington  2006 | M0604-04a | 16 | DQ528878 | e | 2 | DQ313495 | 185 | GQ922901 | 252 | A | no PgiC1 |
| lyrata |  |  | USA; North Carolina; Mayodan | 36,6833  -80,6167 | D. Remington  2006 | M0611-02b | 16 | DQ528878 | e | 2 | DQ313495 | 185 | GQ922901 | 252 | A | no PgiC1 |
| lyrata |  |  | USA; North Carolina; Mayodan | 36,6833  -80,6167 | D. Remington  2006 | M0620-04 | 16 | DQ528878 | e | 2 | DQ313495 | 185 | GQ922901 | 252 | A |  |
| lyrata |  |  | USA; North Carolina; Mayodan | 36,6833  -80,6167 | D. Remington  2006 | M0625-03a | 16 | DQ528878 | e | 2 | DQ313495 | 185 | GQ922901 | 252 | A | no PgiC1 |
| lyrata |  |  | USA; North Carolina; Mayodan | 36,6833  -80,6167 | D. Remington  2006 | M0630-01 | 16 | DQ528878 | e | 2 | DQ313495 | 185 | GQ922901 | 252 | A | no PgiC1 |
| lyrata |  |  | USA; North Carolina; Mayodan | 36,6833  -80,6167 | D. Remington  2006 | M0634-01 | 16 | DQ528878 | e | 2 | DQ313495 | 185 | GQ922901 | 252 | A | no PgiC1 |
| lyrata |  |  | USA; North Carolina; Mayodan | 36,4142  -79,9708 | B. Mable | NCM-2 | 16 | DQ528878 | e | 2 | DQ313495 | 181 | DQ922897 | 248 | A | no PgiC1 |
| lyrata |  |  | USA; North Carolina; Mayodan | 36,4142  -79,9708 | B. Mable | NCM-3 | 16 | DQ528878 | e | 2 | DQ313495 | 181 | DQ922897 | 248 | A | no PgiC1 |
| lyrata |  |  | USA; Ohio; Kitty Todd Nature Preserve | 41,6200  -83,7900 | B. Mable | KTT-1 | 16 | DQ528878 | e | 2 | DQ313495 | 183 | GQ922899 | 250 | BF | no PgiC1 |
| lyrata |  |  | USA; Ohio; Kitty Todd Nature Preserve | 41,6200  -83,7900 | B. Mable | KTT-2 | 109 | GQ922908 | e |  |  | 183 | GQ922899 |  |  |  |
| lyrata |  |  | USA; Ohio; Lake Erie: Headland Dunes State Nature Preserve | 41,7600  -81,2900 | B. Mable | HDC-4 |  |  |  | 2 | DQ313495 | 180 | GQ922896 | 247 | A | no PgiC1 |
| lyrata |  |  | USA; Ohio; Lake Erie: Headland Dunes State Nature Preserve | 41,7600  -81,2900 | B. Mable | HDC-5 |  |  |  | 2 | DQ313495 | 180 | GQ922896 | 247 | A | no PgiC1 |
| lyrata | Arabis lyrata L. | CAS  80860 | USA; Ohio; Lake Erie: Sandusky | 41,4489  -82,7081 | W.A. Wellerman  01.05.1931 | Card0279 |  |  |  |  |  | 2 | DQ528962 |  |  |  |
| lyrata |  |  | USA; Pennsylvania; Lake Erie: Presque Isle State Park | 42,1000  -80,0400 | B. Mable | PRI-A1 |  |  |  | 2 | DQ313495 | 180 | GQ922896 | 247 | A | no PgiC1 |
| lyrata |  |  | USA; Pennsylvania; Lake Erie: Presque Isle State Park | 42,1000  -80,0400 | B. Mable | PRI-A2 |  |  |  | 2 | DQ313495 | 180 | GQ922896 | 247 | A | no PgiC1 |
| lyrata |  |  | Ioma Marsh | 41,1800  -73,5800 | B. Mable | IOM-1 | 108 | GQ922907 | e | 2 | DQ313495 | 180 | GQ922896 | 247 | A | no PgiC1 |
| lyrata |  |  | Ioma Marsh | 41,1800  -73,5800 | B. Mable | IOM-3 | 108 | GQ922907 | e | 2 | DQ313495 | 180 | GQ922896 | 247 | A | no PgiC1 |
| petraea | Cardaminopsis arenosa (L.) Hay. | LI  170954 | AUT; Lower Austria; 2 km NE Melk, E Danube bridge, junction of federal road and street to Spielberg | 48,2397  15,3500 | J. Walter  23.04.1994 | Card0164 | 1 | DQ528814 | a | 2 | DQ313495 | 7 | DQ528967 | 29 | C |  |
| petraea | Cardaminopsis petraea (L.) Hiit. | W  1965-20172 | AUT; Lower Austria; along street from Freiland to Lilienfelder Hütte | 47,9803  15,5717 | G. Leute & A. Polatschek | Card0461 | 1 | DQ528814 | a | 2 | DQ313495 | 49 | DQ529046 | 22 | C |  |
| petraea | Cardaminopsis petraea (L.) Hiit. | W  1965-20168 | AUT; Lower Austria; Bad Vöslau, Merkenstein, Hoher Rain | 47,9833 16,1511 | F. Krendl & A. Polatschek | Card0464 | 17 | DQ528879 | a | 2 | DQ313495 |  |  |  |  |  |
| petraea | Cardaminopsis petraea (L.) Hiit. | HEID  501027 | AUT; Lower Austria; Baden, Castle ruin Rauheneck | 48,0068  16,2063 | M.M. Matschinger  27.04.2004 | 77-03 | 13 | DQ528856 | a | 2 | DQ313495 | 7 | DQ528967 | 29 | C |  |
| petraea | Cardaminopsis petraea (L.) Hiit. | HEID  500364 | AUT; Lower Austria; Baden, Castle ruin Rauheneck | 48,0069  16,2061 | M. Koch  01.07.1999 | Ca20 | 5 | DQ528818 | a | 6 | DQ313499 | 47 | DQ529044 | 85 | B |  |
| petraea | Cardaminopsis petraea (L.) Hiit. | HEID  501019 | AUT; Lower Austria; between Reichental and Pernitz, at railway bridge across Piesting River | 47,8839  15,9932 | M.M. Matschinger  07.05.2004 | 86-02 | 8 | DQ528821 | a | 2 | DQ313495 | 108 | DQ528979 | 52 | C |  |
| petraea | Arabis petraea Lam. | LI | AUT; Lower Austria; Brühl | 48,0833  16,2500 | A. Dürrnberger  06.05.1878 | Card0154 |  |  |  | 2 | DQ313495 | 106 | DQ528977 | 40 | C |  |
| petraea | Cardaminopsis petraea (L.) Hiit. | HEID  500385 | AUT; Lower Austria; Dunkelstein Forest: Aggsbach-Dorf, Gurhof | 48,2983  15,4481 | M. Koch  01.05.2000 | Ca11 | 1 | DQ528814 | a | 2 | DQ313495 | 49 | DQ529046 | 22 | C |  |
| petraea | Cardaminopsis petraea (L.) Hiit. | HEID  500384 | AUT; Lower Austria; Dunkelstein Forest: Aggsbach-Dorf, Gurhof | 48,3000  15,4542 | M. Koch  01.06.2000 | Ca23 | 1 | DQ528814 | a | 2 | DQ313495 | 7 | DQ528967 | 29 | C |  |
| petraea | Cardaminopsis petraea (L.) Hiit. | HEID  500197 | AUT; Lower Austria; Dunkelstein Forest: between Rossatz and Mautern, Windstallgraben, Tränktalwände | 48,3778  15,5170 | M.M. Matschinger  15.04.2003 | 12M-01 |  |  |  | 2 | DQ313495 | 7 | DQ528967 | 29 | C |  |
| petraea | Cardaminopsis petraea (L.) Hiit. | HEID  500197 | AUT; Lower Austria; Dunkelstein Forest: between Rossatz and Mautern, Windstallgraben, Tränktalwände | 48,3778  15,5170 | M.M. Matschinger  15.04.2003 | 12M-03 |  |  |  | 2 | DQ313495 | 13 | DQ529003 | 11 | C |  |
| petraea | Cardaminopsis petraea (L.) Hiit. | HEID  500197 | AUT; Lower Austria; Dunkelstein Forest: between Rossatz and Mautern, Windstallgraben, Tränktalwände | 48,3778  15,5170 | M.M. Matschinger  15.04.2003 | 12M-09 |  |  |  | 2 | DQ313495 | 35 | DQ529032 | 151 |  |  |
| petraea | Cardaminopsis petraea (L.) Hiit. | HEID  500094 | AUT; Lower Austria; Dunkelstein Forest: E Aggsbach-Dorf, W Gurhof, Mitterbachgraben, below forest road up to Bärenköpfl | 48,2967  15,4519 | M.M. Matschinger  01.05.2003 | 46M-01 |  |  |  | 2 | DQ313495 | 7 | DQ528967 | 29 | C |  |
| petraea | Cardaminopsis petraea (L.) Hiit. | HEID  500080 | AUT; Lower Austria; Dunkelstein Forest: from Aggsbach-Dorf to Gamsach, junction to Maria Langegg | 48,3081  15,4419 | M.M. Matschinger  12.05.2003 | 70M-01 |  |  |  | 2 | DQ313495 | 7 | DQ528967 | 29 | C |  |
| petraea | Cardaminopsis petraea (L.) Hiit. | HEID  500080 | AUT; Lower Austria; Dunkelstein Forest: from Aggsbach-Dorf to Gamsach, junction to Maria Langegg | 48,3081  15,4419 | M.M. Matschinger  12.05.2003 | 70M-02 |  |  |  | 2 | DQ313495 | 19 | DQ529016 | 13 | C |  |
| petraea | Cardaminopsis petraea (L.) Hiit. | HEID  500096 | AUT; Lower Austria; Dunkelstein Forest: N Paudorf, Steinaweg | 48,3703  15,5947 | M.M. Matschinger  01.05.2003 | 45M-01 |  |  |  | 2 | DQ313495 | 7 | DQ528967 | 29 | C |  |
| petraea | Cardaminopsis petraea (L.) Hiit. | HEID  500095 | AUT; Lower Austria; Dunkelstein Forest: N Paudorf, Steinaweg | 48,3703  15,5947 | M.M. Matschinger  01.05.2003 | 45M-02 |  |  |  | 2 | DQ313495 | 19 | DQ529016 | 13 | C |  |
| petraea | Cardaminopsis petraea (L.) Hiit. | HEID  500124/500127 | AUT; Lower Austria; Dunkelstein Forest: NE Aggsbach-Dorf, Knieriegel | 48,3122  15,4528 | M.M. Matschinger  12.05.2003 | 71M-01 |  |  |  | 1 | DQ313494 | 16 | DQ529013 | 1 | AC |  |
| petraea | Cardaminopsis petraea (L.) Hiit. | HEID  500125/500127 | AUT; Lower Austria; Dunkelstein Forest: NE Aggsbach-Dorf, Knieriegel | 48,3122  15,4528 | M.M. Matschinger  12.05.2003 | 71M-02 |  |  |  | 2 | DQ313495 | 19 | DQ529016 | 13 | C |  |
| petraea | Cardaminopsis petraea (L.) Hiit. | HEID  500125 | AUT; Lower Austria; Dunkelstein Forest: NE Aggsbach-Dorf, Knieriegel | 48,3122  15,4528 | M.M. Matschinger  12.05.2003 | 71M-03 |  |  |  | 2 | DQ313495 | 7 | DQ528967 | 29 | C |  |
| petraea | Cardaminopsis petraea (L.) Hiit. | HEID  500126 | AUT; Lower Austria; Dunkelstein Forest: NE Aggsbach-Dorf, Knieriegel | 48,3122  15,4528 | M.M. Matschinger  12.05.2003 | 71M-11 |  |  |  | 2 (3A) | DQ313495 | 18 | DQ529015 | 174 | A |  |
| petraea | Cardaminopsis petraea (L.) Hiit. | HEID  500194 | AUT; Lower Austria; Dunkelstein Forest: SSW Mautern, E street from Mauternbach to Unterbergern | 48,3803  15,5615 | M.M. Matschinger  15.04.2003 | 09M-01 |  |  |  | 2 | DQ313495 | 7 | DQ528967 | 29 | C |  |
| petraea | Cardaminopsis petraea (L.) Hiit. | HEID  500194 | AUT; Lower Austria; Dunkelstein Forest: SSW Mautern, E street from Mauternbach to Unterbergern | 48,3803  15,5615 | M.M. Matschinger  15.04.2003 | 09M-10 |  |  |  | 2 | DQ313495 | 19 | DQ529016 | 13 | C |  |
| petraea | Cardaminopsis petraea (L.) Hiit. | HEID  500077 | AUT; Lower Austria; Dunkelstein Forest: Wolfsteinbachtal, rocks along street to Aggsbach-Dorf | 48,2928  15,4244 | M.M. Matschinger  12.05.2003 | 69M-01 |  |  |  | 2 | DQ313495 | 7 | DQ528967 | 29 | C |  |
| petraea | Cardaminopsis petraea (L.) Hiit. | HEID  500077 | AUT; Lower Austria; Dunkelstein Forest: Wolfsteinbachtal, rocks along street to Aggsbach-Dorf | 48,2928  15,4244 | M.M. Matschinger  12.05.2003 | 69M-02 |  |  |  | 2 | DQ313495 | 19 | DQ529016 | 13 | C |  |
| petraea | Cardaminopsis petraea (L.) Hiit. | HEID  500079 | AUT; Lower Austria; Dunkelstein Forest: Wolfsteinbachtal, rocks along street to Aggsbach-Dorf | 48,2928  15,4244 | M.M. Matschinger  12.05.2003 | 69M-12 |  |  |  | 1 | DQ313494 | 16 | DQ529013 | 1 | AC |  |
| petraea | Cardaminopsis petraea | LI  311003 | AUT; Lower Austria; Eastern Alps: western edge of Steinfelde, NW Winzendorf, hill of Castle ruin Emmerberg | 47,8167  16,1000 | W. Till  03.06.1990 | Card0028 | 11 | DQ528834 | d | 2 | DQ313495 |  |  |  |  |  |
| petraea | Cardaminopsis petraea (L.) Hiit. | W  1967-9810 | AUT; Lower Austria; Gutensteiner Alpen: Dürre Wand, Gauermannhütte | 47,8411  15,9406 | A. Polatschek  06.07.1965 | Card0462 |  |  |  | 2 | DQ313495 |  |  |  |  |  |
| petraea | Cardaminopsis petraea (L.) Hiit. | W  1964-6029 | AUT; Lower Austria; Gutensteiner Alpen: Kiensteiner Öde, mountain top | 47,9964  15,6839 | K. Ronninger  04.06.1905 | Card0457 |  |  |  | 2 | DQ313495 |  |  |  |  |  |
| petraea | Cardaminopsis petraea | W  1978-19683 | AUT; Lower Austria; Gutensteiner Alpen: Kleinzell, Schwarzwaldeck | 47,9975  15,7189 | M. Fischer  14.05.1967 | Card0463 | 1 | DQ528814 | a | 2 | DQ313495 | 7 | DQ528967 | 29 | C |  |
| petraea | Cardaminopsis petraea (L.) Hiit. | W  1967-9818 | AUT; Lower Austria; Höldrichsmühle towards Gaaden | 48,0817  16,2389 | F. Kopetzki  18.06.1941 | Card0460 | 7 | DQ528820 | a | 2 | DQ313495 | 7 | DQ528967 | 29 | C |  |
| petraea | Cardaminopsis petraea (L.) Hiit. | HEID  500356 | AUT; Lower Austria; Lilienfeld, Lilienfelder Hütte | 47,9906  15,5917 | M. Koch  01.07.1999 | Ca33 | 5 | DQ528818 | a | 15 | DQ313508 | 13 | DQ529003 | 4 | K |  |
| petraea | Arabidopsis lyrata ssp. petraea | HEID  502659 | AUT; Lower Austria; NE Limestone Alps: between Lilienfeld and Freiland, SE Schrambach | 47,9981  15,5736 | R. Schmickl, M.M. Matschinger & Gong Wei | 116R-04 |  |  |  | 2 | DQ313495 | 19 | DQ529016 | 13 | C |  |
| petraea | Arabidopsis lyrata ssp. petraea | HEID  502666 | AUT; Lower Austria; NE Limestone Alps: between Lilienfeld and Freiland, SE Schrambach | 47,9981  15,5736 | R. Schmickl, M.M. Matschinger & Gong Wei | 116R-13 |  |  |  | 2 | DQ313495 | 49 | DQ529046 | 22 | C |  |
| petraea |  |  | AUT; Lower Austria; NE Limestone Alps: Castle ruin Mödling | 48,0747  16,2680 | M.M. Matschinger  12.06.2006 | 66-915144-01 |  |  |  | 2 | DQ313495 | 179 | GQ922895 | 245 | C |  |
| petraea | Cardaminopsis petraea (L.) Hiit. | HEID  402196 | AUT; Lower Austria; NE Limestone Alps: Ochsattel, 3 km SSE Hohenberg (Traisen Valley) | 47,8800  15,6233 | R. Schmickl & M. Koch | 402196-01 |  |  |  | 6 | DQ313499 | 162 | FJ477709 | 229 | B |  |
| petraea | Arabidopsis lyrata ssp. petraea | HEID | AUT; Lower Austria; NE Limestone Alps: Pernitz, "quarry" at town limits towards Pottenstein | 47,9019  15,9647 | R. Schmickl & P. Weiss | 120R-01 |  |  |  | 2 | DQ313495 | 131 | DQ529005 | 53 | R |  |
| petraea | Cardaminopsis petraea | LI  392608 | AUT; Lower Austria; NE Limestone Alps: Rax, Großes Höllental, rocks at entrance | 47,7453  15,7636 | 07.07.1997 | Card0032 | 9 | DQ528822 | d | 12 | DQ313505 | 56 | DQ529052 | 141 | AF |  |
| petraea | Cardaminopsis petraea (L.) Hiit. | WU  4422 | AUT; Lower Austria; NE Limestone Alps: Rax, Großes Höllental, rocks at entrance | 47,7333  15,9000 | J. Greimler  07.07.1997 | Card0380 |  |  |  | 2 | DQ313495 | 19 | DQ529016 | 13 | C |  |
| petraea | Arabidopsis lyrata ssp. petraea | HEID  502638 | AUT; Lower Austria; NE Limestone Alps: S St. Veit an der Gölsen, St. Veiter Staff | 48,0339  15,6617 | K. Wernisch | 115R-01 |  |  |  | 2 | DQ313495 | 7 | DQ528967 | 29 | C |  |
| petraea |  |  | AUT; Lower Austria; NE Limestone Alps: street from Pernitz to Pottenstein | 47,9165  15,9783 | M. Koch & R. Schmickl  05.06.2006 | 88-915143-03 |  |  |  | 2 | DQ313495 | 99 | DQ529095 | 54 | V |  |
| petraea |  |  | AUT; Lower Austria; NE Limestone Alps: W Bad Vöslau, rocks near Vöslauer Hütte | 47,9748  16,1590 | M.M. Matschinger  21.06.2006 | 74-915145-02 |  |  |  | 6 | DQ313499 | 47 | DQ529044 | 85 | B |  |
| petraea | Cardaminopsis petraea (L.) Hiit. | W  1967-9802 | AUT; Lower Austria; NW Neunkirchen, Würflach, Johannesbachklamm | 47,7803  16,0425 | A. Polatschek  29.05.1966 | Card0458 |  |  |  | 12 | DQ313505 | 133 | DQ528907 | 43 | AF |  |
| petraea | Cardaminopsis petraea (L.) Hiit. | HEID  501018 | AUT; Lower Austria; Pernitz, street to Pottenstein, opposite house Pottensteiner Straße 25 | 47,9016  15,9660 | M.M. Matschinger  07.05.2004 | 87-02 | 8 | DQ528821 | a | 2 | DQ313495 | 131 | DQ529005 | 53 | R |  |
| petraea | Cardaminopsis petraea (L.) Hiit. | HEID  501001 | AUT; Lower Austria; Rax: Höllental, trail to Großer Kesselgraben, bus station Weichtal-Kesselgraben | 47,7522  15,7494 | M.M. Matschinger & K. Wernisch  22.05.2004 | Ex02-02 | 13 | DQ528856 | a | 2 | DQ313495 | 19 | DQ529016 | 13 | C |  |
| petraea | Cardaminopsis cf. arenosa x Cardaminopsis petraea | HEID  501025 | AUT; Lower Austria; S Lilienfeld, along the way to Castle ruin Hohenberg | 47,9022  15,6271 | M.M. Matschinger  27.04.2004 | 79-02 | 77 | DQ528937 | a | 2 | DQ313495 | 7 | DQ528967 | 29 | C |  |
| petraea | Cardaminopsis petraea x Cardaminopsis arenosa | HEID  501026 | AUT; Lower Austria; S Lilienfeld, Castle ruin Hohenberg | 47,9039  15,6237 | M.M. Matschinger  27.04.2004 | 78-04 |  |  |  | 2 | DQ313495 | 107 | DQ528978 | 51 | C |  |
| petraea | Cardaminopsis petraea (L.) Hiit. | HEID  501020 | AUT; Lower Austria; S Lilienfeld, N Freiland | 47,9839  15,5684 | M.M. Matschinger  27.04.2005 | 85-01 | 15 | DQ528877 | a | 2 | DQ313495 |  |  |  |  |  |
| petraea | Cardaminopsis petraea | LI  284981 | AUT; Lower Austria; Thermenlinie: Bad Vöslau, Hauerberg | 47,9667  16,1500 | H. Luftensteiner  03.05.1975 | Card0030 | 10 | DQ528823 | b | 2 | DQ313495 | 7 | DQ528967 | 29 | C |  |
| petraea | Cardaminopsis petraea (L.) Hiit. | W  2001-10814 | AUT; Lower Austria; Thermenlinie: Mount Anninger | 48,0333  16,2333 | E. Vitek  08.05.1976 | Card0442 |  |  |  | 2 | DQ313495 | 7 | DQ528967 | 29 | C |  |
| petraea | Cardaminopsis arenosa (L.) Hay. | LI  115540 | AUT; Lower Austria; Traisen Valley: Freiland, along railway line | 47,9814  15,5692 | Melzer  20.05.1975 | Card0169 | 97 | DQ528957 | b | 6 | DQ313499 | 28 | DQ529025 | 81 | B |  |
| petraea | Cardaminopsis petraea x Cardaminopsis arenosa | HEID  501021 | AUT; Lower Austria; W Gutenstein, from Kalte Kuchl up to Ochsattel | 47,8889  15,6616 | M.M. Matschinger  27.04.2004 | 84-01 | 100 | DQ528824 | a | 2 | DQ313495 |  |  |  |  |  |
| petraea | Cardaminopsis arenosa x Cardaminopsis petraea | W  2001-10803 | AUT; Lower Austria; Wachau: 1 km S Aggsbach-Dorf | 48,2667  15,4000 | E. Vitek  30.04.1981 | Card0410 | 2 | DQ528815 | a | 2 | DQ313495 | 7 | DQ528967 | 29 | C |  |
| petraea | Cardaminopsis petraea | LI  329971 | AUT; Lower Austria; Wachau: 1.8 km WNW church of Schwallenbach, eastern slope of Jauerling towards Danube River, ~0.2 km SW road bend at "Platzl" | 48,3333  15,3667 | B. Wallnöfer  01.05.1997 | Card0029 | 12 | DQ528845 | a | 6 | DQ313499 | 7 | DQ528967 | 94 | J |  |
| petraea | Cardaminopsis petraea | W  2002-376 | AUT; Lower Austria; Wachau: Aggsbach Markt, trail to Froschkopf [noticeable rock] | 48,2833  15,4000 | J. Schneeweiß  07.07.1980 | Card0443 |  |  |  | 2 | DQ313495 |  |  |  |  |  |
| petraea | Cardaminopsis petraea (L.) Hiit. | LI  876188 | AUT; Lower Austria; Wachau: Aggsbach-Dorf | 48,2942  15,4264 | Dr. Mitterd.  03.05.1986 | Card0152 | 1 | DQ528814 | a | 2 | DQ313495 | 7 | DQ528967 | 29 | C |  |
| petraea | Cardaminopsis petraea (L.) Hiit. | HEID  500100 | AUT; Lower Austria; Wachau: along street from 200 m N Oberkienstock to quarry | 48,3833  15,4650 | M.M. Matschinger  02.05.2003 | 49M-01 |  |  |  | 2 | DQ313495 | 7 | DQ528967 | 29 | C |  |
| petraea | Cardaminopsis petraea (L.) Hiit. | HEID  500101 | AUT; Lower Austria; Wachau: along street from 200 m N Oberkienstock to quarry | 48,3833  15,4650 | M.M. Matschinger  02.05.2003 | 49M-03 |  |  |  | 2 | DQ313495 | 19 | DQ529016 | 13 | C |  |
| petraea | Cardaminopsis petraea (L.) Hiit. | HEID  500103 | AUT; Lower Austria; Wachau: Bacharnsdorf, Kupfertal, close to house near creek | 48,3667  15,4500 | M.M. Matschinger  02.05.2003 | 52M-02 |  |  |  | 1 | DQ313494 | 7 | DQ528967 | 167 | AL |  |
| petraea | Cardaminopsis petraea (L.) Hiit. | HEID  500102 | AUT; Lower Austria; Wachau: Bacharnsdorf, Kupfertal, close to house near creek | 48,3667  15,4500 | M.M. Matschinger  02.05.2003 | 52M-03 |  |  |  | 2 | DQ313495 | 7 | DQ528967 | 29 | C |  |
| petraea | Cardaminopsis petraea (L.) Hiit. | HEID  501569 | AUT; Lower Austria; Wachau: Castle ruin Aggstein | 48,2800  15,4228 | M. Koch  01.10.2000 | Ca09 | 1 | DQ528814 | a | 1 | DQ313494 | 16 | DQ529013 | 1 | AC |  |
| petraea | Cardaminopsis petraea (L.) Hiit. | HEID  500375 | AUT; Lower Austria; Wachau: Castle ruin Aggstein | 48,3142  15,4219 | M. Koch  01.05.2000 | Ca22 | 2 | DQ528815 | a | 1 | DQ313494 | 16 | DQ529013 | 1 | AC |  |
| petraea | Cardaminopsis petraea (L.) Hiit. | HEID | AUT; Lower Austria; Wachau: Castle ruin Aggstein | 48,3142  15,4217 | M.M. Matschinger  12.05.2003 | 72M-01 |  |  |  | 1 | DQ313494 | 16 | DQ529013 | 1 | AC |  |
| petraea | Cardaminopsis petraea (L.) Hiit. | HEID  500427 | AUT; Lower Austria; Wachau: Castle ruin Aggstein | 48,3142  15,4217 | M.M. Matschinger  12.05.2003 | 72M-08 |  |  |  | 1 | DQ313494 | 29 | DQ529026 | 152 | AC |  |
| petraea | Cardaminopsis petraea (L.) Hiit. | HEID  500365 | AUT; Lower Austria; Wachau: Castle ruin Dürnstein | 48,3983  15,5225 | M. Koch  01.10.1999 | Ca19 | 1 | DQ528814 | a | 2 | DQ313495 | 7 | DQ528967 | 29 | C |  |
| petraea | Cardaminopsis hispida (L.) Hay. | LI  62036 | AUT; Lower Austria; Wachau: Castle ruin Dürnstein | 48,3953  15,5228 | Melzer  17.05.1984 | Card0157 | 2 | DQ528815 | a | 2 | DQ313495 | 13 | DQ529003 | 11 | C |  |
| petraea | Cardaminopsis petraea (L.) Hiit. | HEID | AUT; Lower Austria; Wachau: Castle ruin Dürnstein and along trail to Fesslhütte | 48,4000  15,5239 | M.M. Matschinger  12.04.2003 | 02M-01 |  |  |  | 2 | DQ313495 | 7 | DQ528967 | 29 | C |  |
| petraea | Cardaminopsis petraea (L.) Hiit. | HEID | AUT; Lower Austria; Wachau: Castle ruin Dürnstein and along trail to Fesslhütte | 48,4000  15,5239 | M.M. Matschinger  12.04.2003 | 02M-05 |  |  |  | 1 | DQ313494 | 16 | DQ529013 | 1 | AC |  |
| petraea | Cardaminopsis petraea (L.) Hiit. | HEID  500183 | AUT; Lower Austria; Wachau: Castle ruin Dürnstein and along trail to Fesslhütte | 48,4000  15,5239 | M.M. Matschinger  17.04.2003 | 17M-01 |  |  |  | 2 | DQ313495 | 17 | DQ529014 | 146 | A |  |
| petraea | Cardaminopsis petraea (L.) Hiit. | HEID  500183 | AUT; Lower Austria; Wachau: Castle ruin Dürnstein and along trail to Fesslhütte | 48,4000  15,5239 | M.M. Matschinger  17.04.2003 | 17M-03 |  |  |  | 2 | DQ313495 | 13 | DQ529003 | 11 | C |  |
| petraea | Cardaminopsis petraea (L.) Hiit. | HEID  500183 | AUT; Lower Austria; Wachau: Castle ruin Dürnstein and along trail to Fesslhütte | 48,4000  15,5239 | M.M. Matschinger  17.04.2003 | 17M-11 |  |  |  | 5 | DQ313498 | 12 | DQ528992 | 70 | Q |  |
| petraea | Cardaminopsis petraea (L.) Hiit. | HEID  500092 | AUT; Lower Austria; Wachau: E crossing of trails from Rossatz to Rotes Kreuz | 48,3928  15,5000 | M.M. Matschinger  30.04.2003 | 39M-01 |  |  |  | 2 | DQ313495 | 7 | DQ528967 | 29 | C |  |
| petraea | Cardaminopsis petraea (L.) Hiit. | HEID  500420 | AUT; Lower Austria; Wachau: E Dürnstein, E Loibenberg | 48,3955  15,5540 | M.M. Matschinger  16.04.2003 | 16M-02 |  |  |  | 2 | DQ313495 | 7 | DQ528967 | 29 | C |  |
| petraea | Cardaminopsis petraea (L.) Hiit. | HEID  500420 | AUT; Lower Austria; Wachau: E Dürnstein, E Loibenberg | 48,3955  15,5540 | M.M. Matschinger  16.04.2003 | 16M-04 |  |  |  | 2 | DQ313495 | 17 | DQ529014 | 146 | A |  |
| petraea | Cardaminopsis petraea (L.) Hiit. | HEID  500420 | AUT; Lower Austria; Wachau: E Dürnstein, E Loibenberg | 48,3955  15,5540 | M.M. Matschinger  16.04.2003 | 16M-06 |  |  |  | 1 | DQ313494 | 16 | DQ529013 | 1 | AC |  |
| petraea | Cardaminopsis petraea (L.) Hiit. | HEID  500421 | AUT; Lower Austria; Wachau: E Dürnstein, E Loibenberg | 48,3955  15,5540 | M.M. Matschinger  16.04.2003 | 16M-08 |  |  |  | 5 | DQ313498 | 12 | DQ528992 | 70 | Q |  |
| petraea | Cardaminopsis petraea (L.) Hiit. | HEID  500421 | AUT; Lower Austria; Wachau: E Dürnstein, E Loibenberg | 48,3955  15,5540 | M.M. Matschinger  16.04.2003 | 16M-09 |  |  |  | 2 | DQ313495 | 13 | DQ529003 | 11 | C |  |
| petraea | Cardaminopsis petraea (L.) Hiit. | HEID  500200 | AUT; Lower Austria; Wachau: E Dürnstein, Loibenberg | 48,3953  15,5417 | M.M. Matschinger  16.04.2003 | 15M-02 |  |  |  | 2 | DQ313495 | 7 | DQ528967 | 29 | C |  |
| petraea | Cardaminopsis petraea (L.) Hiit. | HEID  500198 | AUT; Lower Austria; Wachau: E Dürnstein, small hill N Franzosendenkmal | 48,3970  15,5345 | M.M. Matschinger  16.04.2003 | 13M-02 |  |  |  | 2 | DQ313495 | 13 | DQ529003 | 11 | C |  |
| petraea | Cardaminopsis petraea (L.) Hiit. | HEID  500198 | AUT; Lower Austria; Wachau: E Dürnstein, small hill N Franzosendenkmal | 48,3970  15,5345 | M.M. Matschinger  16.04.2003 | 13M-04 |  |  |  | 2 | DQ313495 | 7 | DQ528967 | 29 | C |  |
| petraea | Cardaminopsis petraea (L.) Hiit. | HEID  500198 | AUT; Lower Austria; Wachau: E Dürnstein, small hill N Franzosendenkmal | 48,3970  15,5345 | M.M. Matschinger  16.04.2003 | 13M-05 |  |  |  | 2 | DQ313495 | 17 | DQ529014 | 146 | A |  |
| petraea | Cardaminopsis petraea (L.) Hiit. | HEID  500121 | AUT; Lower Austria; Wachau: from Rossatzbach to Seegraben | 48,3897  15,5039 | M.M. Matschinger  09.05.2003 | 68M-01 |  |  |  | 2 | DQ313495 | 7 | DQ528967 | 29 | C |  |
| petraea | Cardaminopsis petraea (L.) Hiit. | HEID  500122 | AUT; Lower Austria; Wachau: from Rossatzbach to Seegraben | 48,3897  15,5039 | M.M. Matschinger  09.05.2003 | 68M-06 |  |  |  | 2 | DQ313495 | 13 | DQ529003 | 11 | C |  |
| petraea | Cardaminopsis petraea (L.) Hiit. | HEID  500122 | AUT; Lower Austria; Wachau: from Rossatzbach to Seegraben | 48,3897  15,5039 | M.M. Matschinger  09.05.2003 | 68M-08 |  |  |  | 1 | DQ313494 | 16 | DQ529013 | 1 | AC |  |
| petraea | Cardaminopsis petraea (L.) Hiit. | HEID  500122 | AUT; Lower Austria; Wachau: from Rossatzbach to Seegraben | 48,3897  15,5039 | M.M. Matschinger  09.05.2003 | 68M-11 |  |  |  | 1 | DQ313494 | 25 | DQ529022 | 166 | AC |  |
| petraea | Cardaminopsis petraea (L.) Hiit. | HEID  500176 | AUT; Lower Austria; Wachau: from Rossatzbach up to Toter Berg | 48,3842  15,5167 | M.M. Matschinger  29.04.2003 | 36M-01 |  |  |  | 2 | DQ313495 | 35 | DQ529032 | 151 | C |  |
| petraea | Cardaminopsis petraea (L.) Hiit. | HEID  500176 | AUT; Lower Austria; Wachau: from Rossatzbach up to Toter Berg | 48,3842  15,5167 | M.M. Matschinger  29.04.2003 | 36M-02 |  |  |  | 2 | DQ313495 | 19 | DQ529016 | 13 | C |  |
| petraea | Cardaminopsis petraea (L.) Hiit. | HEID  500178 | AUT; Lower Austria; Wachau: from Rossatzbach up to Toter Berg | 48,3842  15,5167 | M.M. Matschinger  29.04.2003 | 36M-03 |  |  |  | 2 | DQ313495 | 7 | DQ528967 | 29 | C |  |
| petraea | Cardaminopsis petraea (L.) Hiit. | HEID  500178 | AUT; Lower Austria; Wachau: from Rossatzbach up to Toter Berg | 48,3842  15,5167 | M.M. Matschinger  29.04.2003 | 36M-05 |  |  |  | 2 | DQ313495 | 66 | DQ529062 | 162 | C |  |
| petraea | Cardaminopsis petraea (L.) Hiit. | HEID  500109 | AUT; Lower Austria; Wachau: hill (512 mNN) SE Bacharnsdorf | 48,3706  15,4617 | M.M. Matschinger  02.05.2003 | 51M-01 |  |  |  | 2 | DQ313495 | 7 | DQ528967 | 29 | C |  |
| petraea | Cardaminopsis petraea (L.) Hiit. | HEID  500110 | AUT; Lower Austria; Wachau: hill (512 mNN) SE Bacharnsdorf | 48,3706  15,4617 | M.M. Matschinger  02.05.2003 | 51M-09 |  |  |  | 1 | DQ313494 | 29 | DQ529026 | 152 | AC |  |
| petraea | Cardaminopsis petraea (L.) Hiit. | HEID  500110 | AUT; Lower Austria; Wachau: hill (512 mNN) SE Bacharnsdorf | 48,3706  15,4617 | M.M. Matschinger  02.05.2003 | 51M-11 |  |  |  | 2 | DQ313495 | 19 | DQ529016 | 13 | C |  |
| petraea | Cardaminopsis petraea (L.) Hiit. | LI  133924 | AUT; Lower Austria; Wachau: Loiben, Loibenberg | 48,3972  15,5411 | J. Schneeweiß  19.04.1980 | Card0163 | 1 | DQ528814 | a | 2 | DQ313495 | 7 | DQ528967 | 29 | C |  |
| petraea | Cardaminopsis petraea (L.) Hiit. | HEID  500118 | AUT; Lower Austria; Wachau: Mauertal, southern slope | 48,3364  15,4172 | M.M. Matschinger  07.05.2003 | 64M-01 |  |  |  | 2 | DQ313495 | 7 | DQ528967 | 29 | C |  |
| petraea | Cardaminopsis petraea (L.) Hiit. | HEID  500118 | AUT; Lower Austria; Wachau: Mauertal, southern slope | 48,3364  15,4172 | M.M. Matschinger  07.05.2003 | 64M-02 |  |  |  | 6 | DQ313499 | 28 | DQ529026 | 81 | B |  |
| petraea | Cardaminopsis petraea (L.) Hiit. | HEID  500118 | AUT; Lower Austria; Wachau: Mauertal, southern slope | 48,3364  15,4172 | M.M. Matschinger  07.05.2003 | 64M-03 |  |  |  | 1 | DQ313494 | 29 | DQ529026 | 152 | AC |  |
| petraea | Cardaminopsis petraea (L.) Hiit. | HEID  500044 | AUT; Lower Austria; Wachau: Mauertal, western slope | 48,3320  15,4132 | M.M. Matschinger | 62M-01 |  |  |  | 2 | DQ313495 | 7 | DQ528967 | 29 | C |  |
| petraea | Cardaminopsis petraea (L.) Hiit. | HEID  500044 | AUT; Lower Austria; Wachau: Mauertal, western slope | 48,3320  15,4132 | M.M. Matschinger | 62M-02 |  |  |  | 1 | DQ313494 | 29 | DQ529026 | 152 | AC |  |
| petraea | Cardaminopsis petraea (L.) Hiit. | HEID  500046 | AUT; Lower Austria; Wachau: Mauertal, western slope | 48,3320  15,4132 | M.M. Matschinger | 62M-07 |  |  |  | 6 | DQ313499 | 28 | DQ529026 | 81 | B |  |
| petraea | Cardaminopsis petraea (L.) Hiit. | HEID  500045 | AUT; Lower Austria; Wachau: Mauertal, western slope | 48,3320  15,4132 | M.M. Matschinger | 62M-10 |  |  |  | 1 | DQ313494 | 16 | DQ529013 | 1 | AC |  |
| petraea | Cardaminopsis petraea (L.) Hiit. | HEID  501561 | AUT; Lower Austria; Wachau: Mautern, Oberbergern, Ferdinandswarte | 48,3817  15,5433 | M. Koch  01.07.2002 | Ca02 | 1 | DQ528814 | a | 2 | DQ313495 | 22 | DQ529019 | 16 | C |  |
| petraea | Cardaminopsis petraea (L.) Hiit. | LI  30165 | AUT; Lower Austria; Wachau: Melk, SE Aggsbach Markt, Wolfstenbach Valley | 48,2906  15,4261 | Pils  17.04.1988 | Card0156 | 1 | DQ528814 | a | 2 | DQ313495 | 19 | DQ529016 | 13 | C |  |
| petraea | Cardaminopsis petraea (L.) Hiit. | HEID  500059 | AUT; Lower Austria; Wachau: Melk, street to Schönbühel, 300 m in front of bridge across Danube River | 48,2343  15,3430 | M.M. Matschinger  05.05.2003 | 55M-02 |  |  |  | 2 | DQ313495 | 7 | DQ528967 | 29 | C |  |
| petraea | Cardaminopsis petraea (L.) Hiit. | HEID  500129 | AUT; Lower Austria; Wachau: Melk, street to Schönbühel, between bridge across Danube River and mouth of Pielach River | 48,2397  15,3500 | M.M. Matschinger  05.05.2003 | 56M-01 |  |  |  | 9 | DQ313502 | 7 | DQ528967 | 160 | AI |  |
| petraea | Cardaminopsis petraea (L.) Hiit. | HEID  500129 | AUT; Lower Austria; Wachau: Melk, street to Schönbühel, between bridge across Danube River and mouth of Pielach River | 48,2397  15,3500 | M.M. Matschinger  05.05.2003 | 56M-04 |  |  |  | 2 | DQ313495 | 7 | DQ528967 | 29 | C |  |
| petraea | Cardaminopsis petraea (L.) Hiit. | HEID  500428 | AUT; Lower Austria; Wachau: N Castle ruin Aggstein, Gerichtsgraben | 48,3233  15,4167 | M.M. Matschinger  12.05.2003 | 73M-01 |  |  |  | 2 | DQ313495 | 7 | DQ528967 | 29 | C |  |
| petraea | Cardaminopsis petraea (L.) Hiit. | HEID  500428 | AUT; Lower Austria; Wachau: N Castle ruin Aggstein, Gerichtsgraben | 48,3233  15,4167 | M.M. Matschinger  12.05.2003 | 73M-03 |  |  |  | 10 | DQ313503 | 7 | DQ528967 | 175 | AK |  |
| petraea | Cardaminopsis petraea (L.) Hiit. | HEID  500428 | AUT; Lower Austria; Wachau: N Castle ruin Aggstein, Gerichtsgraben | 48,3233  15,4167 | M.M. Matschinger  12.05.2003 | 73M-06 |  |  |  | 1 | DQ313494 | 16 | DQ529013 | 1 | AC |  |
| petraea | Cardaminopsis petraea (L.) Hiit. | HEID  500185 | AUT; Lower Austria; Wachau: N Dürnstein, from valley opening N Fesslhütte to Biratalwand | 48,4072  15,5250 | M.M. Matschinger  17.04.2003 | 18M-01 |  |  |  | 2 | DQ313495 | 17 | DQ529014 | 146 | A |  |
| petraea | Cardaminopsis petraea (L.) Hiit. | HEID | AUT; Lower Austria; Wachau: N Dürnstein, from valley opening N Fesslhütte to Biratalwand | 48,4072  15,5250 | M.M. Matschinger  17.04.2003 | 18M-04 |  |  |  | 1 | DQ313494 | 16 | DQ529013 | 1 | AC |  |
| petraea | Cardaminopsis petraea (L.) Hiit. | HEID  500185 | AUT; Lower Austria; Wachau: N Dürnstein, from valley opening N Fesslhütte to Biratalwand | 48,4072  15,5250 | M.M. Matschinger  17.04.2003 | 18M-09 |  |  |  | 2 | DQ313495 | 7 | DQ528967 | 29 | C |  |
| petraea | Cardaminopsis petraea (L.) Hiit. | HEID  500072 | AUT; Lower Austria; Wachau: N Schönbühel, Blashausgraben | 48,2758  15,4045 | M.M. Matschinger  05.05.2003 | 58M-01 |  |  |  | 2 | DQ313495 | 7 | DQ528967 | 29 | C |  |
| petraea | Cardaminopsis petraea (L.) Hiit. | HEID  500072 | AUT; Lower Austria; Wachau: N Schönbühel, Blashausgraben | 48,2758  15,4045 | M.M. Matschinger  05.05.2003 | 58M-02 |  |  |  | 2 | DQ313495 | 31 | DQ529028 | 169 | C |  |
| petraea | Cardaminopsis petraea (L.) Hiit. | HEID  500073 | AUT; Lower Austria; Wachau: N Schönbühel, Blashausgraben | 48,2758  15,4045 | M.M. Matschinger  05.05.2003 | 58M-08 |  |  |  | 2 | DQ313495 | 19 | DQ529016 | 13 | C |  |
| petraea | Cardaminopsis petraea (L.) Hiit. | HEID  500074 | AUT; Lower Austria; Wachau: N Schönbühel, Blashausgraben | 48,2758  15,4045 | M.M. Matschinger  05.05.2003 | 58M-10 |  |  |  | 2 | DQ313495 | 32 | DQ529029 | 170 | C |  |
| petraea | Cardaminopsis petraea (L.) Hiit. | HEID  500070 | AUT; Lower Austria; Wachau: N Schönbühel, rocks along federal road | 48,2744  15,3933 | M.M. Matschinger  05.05.2003 | 57M-01 |  |  |  | 2 | DQ313495 | 7 | DQ528967 | 29 | C |  |
| petraea | Cardaminopsis petraea (L.) Hiit. | HEID  500070 | AUT; Lower Austria; Wachau: N Schönbühel, rocks along federal road | 48,2744  15,3933 | M.M. Matschinger  05.05.2003 | 57M-06 |  |  |  | 2 | DQ313495 | 30 | DQ529027 | 168 | C |  |
| petraea | Cardaminopsis petraea (L.) Hiit. | HEID  500145 | AUT; Lower Austria; Wachau: N Spitz, loop road up to Michaelerberg | 48,3732  15,4168 | M.M. Matschinger  25.04.2003 | 26M-02 |  |  |  | 2 | DQ313495 | 7 | DQ528967 | 29 | C |  |
| petraea | Cardaminopsis petraea (L.) Hiit. | HEID  500135 | AUT; Lower Austria; Wachau: N Spitz, Mieslingbachgraben, Atzberg, western slope | 48,3828  15,4170 | M.M. Matschinger  25.04.2003 | 27M-01 |  |  |  | 2 | DQ313495 | 7 | DQ528967 | 29 | C |  |
| petraea | Cardaminopsis petraea (L.) Hiit. | HEID  500140 | AUT; Lower Austria; Wachau: N Spitz, Mieslingbachgraben, forest road, S Pop. 27 | 48,3790  15,3668 | M.M. Matschinger  25.04.2003 | 28M-01 |  |  |  | 2 | DQ313495 | 7 | DQ528967 | 29 | C |  |
| petraea | Cardaminopsis petraea (L.) Hiit. | HEID  500199 | AUT; Lower Austria; Wachau: NE Dürnstein, ridge NNE Mähntalgraben towards Starhembergwarte | 48,4017  15,5305 | M.M. Matschinger  16.04.2003 | 14M-01 |  |  |  | 2 | DQ313495 | 7 | DQ528967 | 29 | C |  |
| petraea | Cardaminopsis petraea (L.) Hiit. | HEID  500199 | AUT; Lower Austria; Wachau: NE Dürnstein, ridge NNE Mähntalgraben towards Starhembergwarte | 48,4017  15,5305 | M.M. Matschinger  16.04.2003 | 14M-03 |  |  |  | 1 | DQ313494 | 16 | DQ529013 | 1 | AC |  |
| petraea | Cardaminopsis petraea (L.) Hiit. | HEID  500199 | AUT; Lower Austria; Wachau: NE Dürnstein, ridge NNE Mähntalgraben towards Starhembergwarte | 48,4017  15,5305 | M.M. Matschinger  16.04.2003 | 14M-07 |  |  |  | 1 | DQ313494 | 29 | DQ529026 | 152 |  |  |
| petraea | Cardaminopsis petraea (L.) Hiit. | HEID  500115 | AUT; Lower Austria; Wachau: NE Melk, mouth of Pielach River, rock face on south bank | 48,2375  15,3561 | M.M. Matschinger  03.05.2003 | 53M-01 |  |  |  | 2 | DQ313495 | 7 | DQ528967 | 29 | C |  |
| petraea | Cardaminopsis petraea (L.) Hiit. | HEID  500115 | AUT; Lower Austria; Wachau: NE Melk, mouth of Pielach River, rock face on south bank | 48,2375  15,3561 | M.M. Matschinger  03.05.2003 | 53M-02 |  |  |  | 9 | DQ313502 | 7 | DQ528967 | 160 | AI |  |
| petraea | Cardaminopsis petraea (L.) Hiit. | HEID  500186 | AUT; Lower Austria; Wachau: NNE Dürnstein, SSE Scheibenhof, 100 m E Hengelwand | 48,4078  15,5420 | M.M. Matschinger  12.04.2003 | 01M-01 |  |  |  | 5 | DQ313498 | 12 | DQ528992 | 70 | Q |  |
| petraea | Cardaminopsis petraea (L.) Hiit. | HEID | AUT; Lower Austria; Wachau: NNE Dürnstein, SSE Scheibenhof, 100 m E Hengelwand | 48,4078  15,5420 | M.M. Matschinger  12.04.2003 | 01M-05 |  |  |  | 2 | DQ313495 | 7 | DQ528967 | 29 | C |  |
| petraea | Cardaminopsis petraea (L.) Hiit. | HEID  500186 | AUT; Lower Austria; Wachau: NNE Dürnstein, SSE Scheibenhof, 100 m E Hengelwand | 48,4078  15,5420 | M.M. Matschinger  12.04.2003 | 01M-09 |  |  |  | 1 | DQ313494 | 16 | DQ529013 | 1 | AC |  |
| petraea | Cardaminopsis petraea (L.) Hiit. | HEID  500143 | AUT; Lower Austria; Wachau: NW crossing of trails from Rossatz to Rotes Kreuz | 48,3936  15,4967 | M.M. Matschinger  30.04.2003 | 43M-02 |  |  |  | 2 | DQ313495 | 7 | DQ528967 | 29 | C |  |
| petraea | Cardaminopsis petraea (L.) Hiit. | HEID  500052 | AUT; Lower Austria; Wachau: Oberarnsdorf, Buchental | 48,3533  15,4289 | M.M. Matschinger  07.05.2003 | 60M-01 |  |  |  | 2 | DQ313495 | 7 | DQ528967 | 29 | C |  |
| petraea | Cardaminopsis petraea (L.) Hiit. | HEID  500111 | AUT; Lower Austria; Wachau: rocks nearby trail N Bacharnsdorf | 48,3722  15,4542 | M.M. Matschinger  02.05.2003 | 50M-01 |  |  |  | 1 | DQ313494 | 25 | DQ529022 | 166 | AC |  |
| petraea | Cardaminopsis petraea (L.) Hiit. | HEID  500111 | AUT; Lower Austria; Wachau: rocks nearby trail N Bacharnsdorf | 48,3722  15,4542 | M.M. Matschinger  02.05.2003 | 50M-02 |  |  |  | 2 | DQ313495 | 7 | DQ528967 | 29 | C |  |
| petraea | Cardaminopsis petraea (L.) Hiit. | HEID  500111 | AUT; Lower Austria; Wachau: rocks nearby trail N Bacharnsdorf | 48,3722  15,4542 | M.M. Matschinger  02.05.2003 | 50M-05 |  |  |  | 1 | DQ313494 | 16 | DQ529013 | 1 | AC |  |
| petraea | Cardaminopsis petraea (L.) Hiit. | HEID  500075 | AUT; Lower Austria; Wachau: S Aggsbach-Dorf, above federal road | 48,2847  15,4068 | M.M. Matschinger  05.05.2003 | 59M-01 |  |  |  | 9 | DQ313502 | 7 | DQ528967 | 160 | AI |  |
| petraea | Cardaminopsis petraea (L.) Hiit. | HEID  500075 | AUT; Lower Austria; Wachau: S Aggsbach-Dorf, above federal road | 48,2847  15,4068 | M.M. Matschinger  05.05.2003 | 59M-02 |  |  |  | 2 | DQ313495 | 19 | DQ529016 | 13 | C |  |
| petraea | Cardaminopsis petraea (L.) Hiit. | HEID | AUT; Lower Austria; Wachau: S Aggsbach-Dorf, above federal road | 48,2847  15,4068 | M.M. Matschinger  05.05.2003 | 59M-05 |  |  |  | 2 | DQ313495 | 28 | DQ529026 | 78 | A |  |
| petraea | Cardaminopsis petraea (L.) Hiit. | HEID  500130 | AUT; Lower Austria; Wachau: S Aggsbach-Markt, southern end of Donauleiten | 48,2764  15,3794 | M.M. Matschinger | 75M-01 |  |  |  | 2 | DQ313495 | 7 | DQ528967 | 29 | C |  |
| petraea | Cardaminopsis petraea (L.) Hiit. | HEID  500132 | AUT; Lower Austria; Wachau: S Aggsbach-Markt, southern end of Donauleiten | 48,2764  15,3794 | M.M. Matschinger | 75M-02 |  |  |  | 2 | DQ313495 | 19 | DQ529016 | 13 | C |  |
| petraea | Cardaminopsis petraea (L.) Hiit. | HEID  500130 | AUT; Lower Austria; Wachau: S Aggsbach-Markt, southern end of Donauleiten | 48,2764  15,3794 | M.M. Matschinger | 75M-04 |  |  |  | 9 | DQ313502 | 19 | DQ529016 | 176 | AI |  |
| petraea | Cardaminopsis petraea (L.) Hiit. | HEID  500056 | AUT; Lower Austria; Wachau: S Oberarnsdorf, Rote Wand | 48,3486  15,4233 | M.M. Matschinger | 61M-01 |  |  |  | 2 | DQ313495 | 7 | DQ528967 | 29 | C |  |
| petraea | Cardaminopsis petraea (L.) Hiit. | HEID  500058 | AUT; Lower Austria; Wachau: S Oberarnsdorf, Rote Wand | 48,3486  15,4233 | M.M. Matschinger | 61M-10 |  |  |  | 2 | DQ313495 | 33 | DQ529030 | 171 | C |  |
| petraea | Cardaminopsis petraea (L.) Hiit. | HEID  500049 | AUT; Lower Austria; Wachau: S Oberarnsdorf, Trauntal, southern slope | 48,3428  15,4197 | M.M. Matschinger  07.05.2003 | 65M-02 |  |  |  | 2 | DQ313495 | 7 | DQ528967 | 29 | C |  |
| petraea | Cardaminopsis petraea (L.) Hiit. | HEID  500049 | AUT; Lower Austria; Wachau: S Oberarnsdorf, Trauntal, southern slope | 48,3428  15,4197 | M.M. Matschinger  07.05.2003 | 65M-03 |  |  |  | 1 | DQ313494 | 29 | DQ529026 | 152 | AC |  |
| petraea | Cardaminopsis petraea (L.) Hiit. | HEID  500051 | AUT; Lower Austria; Wachau: S Oberarnsdorf, Trauntal, southern slope | 48,3428  15,4197 | M.M. Matschinger  07.05.2003 | 65M-09 |  |  |  | 1 | DQ313494 | 39 | DQ529036 | 178 | AC |  |
| petraea | Cardaminopsis petraea (L.) Hiit. | HEID  500154 | AUT; Lower Austria; Wachau: Schwallenbach, batter close to transformer | 48,3411  15,4011 | M.M. Matschinger  26.04.2003 | 31M-01 |  |  |  | 2 | DQ313495 | 19 | DQ529016 | 13 | C |  |
| petraea | Cardaminopsis petraea (L.) Hiit. | HEID | AUT; Lower Austria; Wachau: Schwallenbach, batter close to transformer | 48,3411  15,4011 | M.M. Matschinger  26.04.2003 | 31M-03 |  |  |  | 2 | DQ313495 | 7 | DQ528967 | 29 | C |  |
| petraea | Arabidopsis lyrata ssp. petraea | HEID  502510 | AUT; Lower Austria; Wachau: SE Rossatz, forest road, 0.5 km NW Pop. 68 | 48,3989  15,5017 | R. Schmickl & M.M. Matschinger  25.04.2005 | 102R-01 |  |  |  | 2 | DQ313495 | 7 | DQ528967 | 29 | C |  |
| petraea | Arabidopsis lyrata ssp. petraea | HEID  502522 | AUT; Lower Austria; Wachau: SE Rossatz, forest road, 0.5 km NW Pop. 68 | 48,3989  15,5017 | R. Schmickl & M.M. Matschinger  25.04.2005 | 102R-13 |  |  |  | 1 | DQ313494 | 25 | DQ529022 | 166 | AC |  |
| petraea | Arabidopsis lyrata ssp. petraea | HEID  502526 | AUT; Lower Austria; Wachau: SE Rossatz, forest road, 0.5 km NW Pop. 68 | 48,3989  15,5017 | R. Schmickl & M.M. Matschinger  25.04.2005 | 102R-17 |  |  |  | 2 | DQ313495 | 13 | DQ529003 | 11 | C |  |
| petraea | Cardaminopsis petraea (L.) Hiit. | HEID  500172 | AUT; Lower Austria; Wachau: SE St. Lorenz, Steinige Ries, trail | 48,3931  15,4833 | M.M. Matschinger  29.04.2003 | 37M-01 |  |  |  | 2 | DQ313495 | 38 | DQ529035 | 161 | C |  |
| petraea | Cardaminopsis petraea (L.) Hiit. | HEID  500171 | AUT; Lower Austria; Wachau: SE St. Lorenz, Steinige Ries, trail | 48,3931  15,4833 | M.M. Matschinger  29.04.2003 | 37M-05 |  |  |  | 2 | DQ313495 | 7 | DQ528967 | 29 | C |  |
| petraea | Cardaminopsis petraea (L.) Hiit. | HEID  500169 | AUT; Lower Austria; Wachau: SE St. Lorenz, Steinige Ries, trail | 48,3931  15,4833 | M.M. Matschinger  29.04.2003 | 37M-10 |  |  |  | 1 | DQ313494 | 16 | DQ529013 | 1 | AC |  |
| petraea | Cardaminopsis petraea (L.) Hiit. | HEID  500175 | AUT; Lower Austria; Wachau: SE St. Lorenz, Steinige Ries, trail | 48,3931  15,4833 | M.M. Matschinger  29.04.2003 | 38M-07 |  |  |  | 1 | DQ313494 | 29 | DQ529026 | 152 | AC |  |
| petraea | Cardaminopsis petraea (L.) Hiit. | HEID  500147 | AUT; Lower Austria; Wachau: Spitz, Castle ruin Hinterhaus | 48,3594  15,4081 | M.M. Matschinger  26.04.2003 | 29M-01 |  |  |  | 1 | DQ313494 | 16 | DQ529013 | 1 | AC |  |
| petraea | Cardaminopsis petraea (L.) Hiit. | HEID  500147 | AUT; Lower Austria; Wachau: Spitz, Castle ruin Hinterhaus | 48,3594  15,4081 | M.M. Matschinger  26.04.2003 | 29M-02 |  |  |  | 2 | DQ313495 | 7 | DQ528967 | 29 | C |  |
| petraea | Cardaminopsis petraea (L.) Hiit. | HEID  500150 | AUT; Lower Austria; Wachau: Spitz, SW Castle ruin Hinterhaus | 48,3594  15,4064 | M.M. Matschinger  26.04.2003 | 30M-02 |  |  |  | 2 | DQ313495 | 7 | DQ528967 | 29 | C |  |
| petraea | Cardaminopsis petraea (L.) Hiit. | HEID  500151 | AUT; Lower Austria; Wachau: Spitz, SW Castle ruin Hinterhaus | 48,3594  15,4064 | M.M. Matschinger  26.04.2003 | 30M-04 |  |  |  | 1 | DQ313494 | 16 | DQ529013 | 1 | AC |  |
| petraea | Cardaminopsis petraea (L.) Hiit. | HEID  500152 | AUT; Lower Austria; Wachau: Spitz, SW Castle ruin Hinterhaus | 48,3594  15,4064 | M.M. Matschinger  26.04.2003 | 30M-08 |  |  |  | 16 | DQ313509 | 19 | DQ529016 | 157 | AH |  |
| petraea | Cardaminopsis petraea (L.) Hiit. | HEID  500151 | AUT; Lower Austria; Wachau: Spitz, SW Castle ruin Hinterhaus | 48,3594  15,4064 | M.M. Matschinger  26.04.2003 | 30M-12 |  |  |  | 1 | DQ313494 | 19 | DQ529016 | 177 | AL |  |
| petraea | Cardaminopsis petraea (L.) Hiit. | HEID  500165 | AUT; Lower Austria; Wachau: Spitz, Tausendeimerberg | 48,3633  15,4119 | M.M. Matschinger  24.04.2003 | 25M-02 |  |  |  | 2 | DQ313495 | 7 | DQ528967 | 29 | C |  |
| petraea | Cardaminopsis petraea (L.) Hiit. | HEID  500165 | AUT; Lower Austria; Wachau: Spitz, Tausendeimerberg | 48,3633  15,4119 | M.M. Matschinger  24.04.2003 | 25M-06 |  |  |  | 2 | DQ313495 | 19 | DQ529016 | 13 | C |  |
| petraea | Cardaminopsis petraea (L.) Hiit. | HEID  500087 | AUT; Lower Austria; Wachau: SSW Rossatz, Waldandacht, forest road | 48,3917  15,4897 | M.M. Matschinger  30.04.2003 | 40M-01 |  |  |  | 1 | DQ313494 | 16 | DQ529013 | 1 | AC |  |
| petraea | Cardaminopsis petraea (L.) Hiit. | HEID  500087 | AUT; Lower Austria; Wachau: SSW Rossatz, Waldandacht, forest road | 48,3917  15,4897 | M.M. Matschinger  30.04.2003 | 40M-02 |  |  |  | 2 | DQ313495 | 7 | DQ528967 | 29 | C |  |
| petraea | Cardaminopsis petraea (L.) Hiit. | HEID  500086 | AUT; Lower Austria; Wachau: SSW Rossatz, Waldandacht, forest road | 48,3917  15,4897 | M.M. Matschinger  30.04.2003 | 40M-03 |  |  |  | 5 | DQ313498 | 7 | DQ528967 | 163 | C |  |
| petraea | Cardaminopsis petraea (L.) Hiit. | HEID  500086 | AUT; Lower Austria; Wachau: SSW Rossatz, Waldandacht, forest road | 48,3917  15,4897 | M.M. Matschinger  30.04.2003 | 40M-04 |  |  |  | 7 | DQ313500 | 7 | DQ528967 | 164 | AJ |  |
| petraea | Cardaminopsis petraea (L.) Hiit. | HEID | AUT; Lower Austria; Wachau: SSW Rossatz, Waldandacht, forest road, N Pop. 41 | 48,3958  15,4947 | M.M. Matschinger  30.04.2003 | 42M-01 |  |  |  | 2 | DQ313495 | 7 | DQ528967 | 29 | C |  |
| petraea | Cardaminopsis petraea (L.) Hiit. | HEID | AUT; Lower Austria; Wachau: SSW Rossatz, Waldandacht, forest road, N Pop. 41 | 48,3958  15,4947 | M.M. Matschinger  30.04.2003 | 42M-04 |  |  |  | 1 | DQ313494 | 16 | DQ529013 | 1 | AC |  |
| petraea | Cardaminopsis petraea (L.) Hiit. | HEID  500047 | AUT; Lower Austria; Wachau: St. Johann im Mauerthale, towards Tischwand | 48,3292  15,4150 | M.M. Matschinger  07.05.2003 | 63M-01 |  |  |  | 1 | DQ313494 | 16 | DQ529013 | 1 | AC |  |
| petraea | Cardaminopsis petraea (L.) Hiit. | HEID  500048 | AUT; Lower Austria; Wachau: St. Johann im Mauerthale, towards Tischwand | 48,3292  15,4150 | M.M. Matschinger  07.05.2003 | 63M-06 |  |  |  | 1 | DQ313494 | 25 | DQ529022 | 166 | AC |  |
| petraea | Cardaminopsis petraea (L.) Hiit. | HEID  500048 | AUT; Lower Austria; Wachau: St. Johann im Mauerthale, towards Tischwand | 48,3292  15,4150 | M.M. Matschinger  07.05.2003 | 63M-07 |  |  |  | 1 | DQ313494 | 23 | DQ529020 | 172 | AC |  |
| petraea | Cardaminopsis petraea (L.) Hiit. | HEID  500066 | AUT; Lower Austria; Wachau: street S Seeb to Aggsbach-Markt, Krumme Hand | 48,2887  15,3833 | M.M. Matschinger | 35M-01 |  |  |  | 2 | DQ313495 | 38 | DQ529035 | 161 | C |  |
| petraea | Cardaminopsis petraea (L.) Hiit. | HEID  500066 | AUT; Lower Austria; Wachau: street S Seeb to Aggsbach-Markt, Krumme Hand | 48,2887  15,3833 | M.M. Matschinger | 35M-02 |  |  |  | 2 | DQ313495 | 7 | DQ528967 | 29 | C |  |
| petraea | Cardaminopsis petraea (L.) Hiit. | HEID  500082 | AUT; Lower Austria; Wachau: street up to Jauerling, between junction Vießling and Ödrent | 48,3515  15,3760 | M.M. Matschinger  28.04.2003 | 32M-02 |  |  |  | 2 | DQ313495 | 7 | DQ528967 | 29 | C |  |
| petraea | Cardaminopsis petraea (L.) Hiit. | HEID  500082 | AUT; Lower Austria; Wachau: street up to Jauerling, between junction Vießling and Ödrent | 48,3515  15,3760 | M.M. Matschinger  28.04.2003 | 32M-04 |  |  |  | 2 | DQ313495 | 27 | DQ529024 | 158 |  |  |
| petraea | Cardaminopsis petraea (L.) Hiit. | HEID  500062 | AUT; Lower Austria; Wachau: street up to Jauerling, from Platzl | 48,3440  15,3758 | M.M. Matschinger | 34M-01 |  |  |  | 6 | DQ313499 | 65 | DQ529061 | 159 | B |  |
| petraea | Cardaminopsis petraea (L.) Hiit. | HEID  500062 | AUT; Lower Austria; Wachau: street up to Jauerling, from Platzl | 48,3440  15,3758 | M.M. Matschinger | 34M-02 |  |  |  | 2 | DQ313495 | 7 | DQ528967 | 29 | C |  |
| petraea | Cardaminopsis petraea (L.) Hiit. | HEID | AUT; Lower Austria; Wachau: street up to Jauerling, from Platzl | 48,3440  15,3758 | M.M. Matschinger | 34M-12 |  |  |  | 9 | DQ313502 | 7 | DQ528967 | 160 | AI |  |
| petraea | Cardaminopsis petraea (L.) Hiit. | HEID | AUT; Lower Austria; Wachau: street up to Jauerling, hairpin bend, Schweineck | 48,3462  15,3833 | M.M. Matschinger  28.04.2003 | 33M-01 |  |  |  | 2 | DQ313495 | 7 | DQ528967 | 29 | C |  |
| petraea | Cardaminopsis petraea (L.) Hiit. | HEID  500168 | AUT; Lower Austria; Wachau: trail from St. Michael northward | 48,3731  15,4367 | M.M. Matschinger  24.04.2003 | 24M-02 |  |  |  | 2 | DQ313495 | 7 | DQ528967 | 29 | C |  |
| petraea | Cardaminopsis petraea (L.) Hiit. | HEID  500106 | AUT; Lower Austria; Wachau: Unterkienstock to trail to Hirschwand | 48,3833  15,4797 | M.M. Matschinger  02.05.2003 | 47M-01 |  |  |  | 2 | DQ313495 | 7 | DQ528967 | 29 | C |  |
| petraea | Cardaminopsis petraea (L.) Hiit. | HEID  500106 | AUT; Lower Austria; Wachau: Unterkienstock to trail to Hirschwand | 48,3833  15,4797 | M.M. Matschinger  02.05.2003 | 47M-02 |  |  |  | 2 | DQ313495 | 16 | DQ529013 | 1 | AC |  |
| petraea | Cardaminopsis petraea (L.) Hiit. | HEID  500107 | AUT; Lower Austria; Wachau: Unterkienstock, below Seekopf | 48,3764  15,4714 | M.M. Matschinger  02.05.2003 | 48M-01 |  |  |  | 1 | DQ313494 | 16 | DQ529013 | 1 | AC |  |
| petraea | Cardaminopsis petraea (L.) Hiit. | HEID  500107 | AUT; Lower Austria; Wachau: Unterkienstock, below Seekopf | 48,3764  15,4714 | M.M. Matschinger  02.05.2003 | 48M-02 |  |  |  | 1 | DQ313494 | 67 | DQ529063 | 165 | AC |  |
| petraea | Cardaminopsis petraea (L.) Hiit. | HEID  500108 | AUT; Lower Austria; Wachau: Unterkienstock, below Seekopf | 48,3764  15,4714 | M.M. Matschinger  02.05.2003 | 48M-03 |  |  |  | 2 | DQ313495 | 7 | DQ528967 | 29 | C |  |
| petraea | Cardaminopsis petraea (L.) Hiit. | HEID  500108 | AUT; Lower Austria; Wachau: Unterkienstock, below Seekopf | 48,3764  15,4714 | M.M. Matschinger  02.05.2003 | 48M-04 |  |  |  | 2 | DQ313495 | 13 | DQ529003 | 11 | C |  |
| petraea | Cardaminopsis petraea (L.) Hiit. | HEID  500108 | AUT; Lower Austria; Wachau: Unterkienstock, below Seekopf | 48,3764  15,4714 | M.M. Matschinger  02.05.2003 | 48M-05 |  |  |  | 6 | DQ313499 | 28 | DQ529026 | 81 | B |  |
| petraea | Arabidopsis lyrata ssp. petraea | HEID  502530 | AUT; Lower Austria; Wachau: valley 2 km S Pop. 62, western slope | 48,3300  15,4161 | R. Schmickl & M.M. Matschinger  28.04.2005 | 103R-01 |  |  |  | 1 | DQ313494 | 25 | DQ529022 | 166 | AC |  |
| petraea | Arabidopsis lyrata ssp. petraea | HEID  502531 | AUT; Lower Austria; Wachau: valley 2 km S Pop. 62, western slope | 48,3300  15,4161 | R. Schmickl & M.M. Matschinger  28.04.2005 | 103R-02 |  |  |  | 1 | DQ313494 | 23 | DQ529020 | 172 | AC |  |
| petraea | Arabidopsis lyrata ssp. petraea | HEID  502533 | AUT; Lower Austria; Wachau: valley 2 km S Pop. 62, western slope | 48,3300  15,4161 | R. Schmickl & M.M. Matschinger  28.04.2005 | 103R-04 |  |  |  | 1 | DQ313494 | 16 | DQ529013 | 1 | AC |  |
| petraea | Arabidopsis lyrata ssp. petraea | HEID  502544 | AUT; Lower Austria; Wachau: valley 2 km S Pop. 62, western slope | 48,3300  15,4161 | R. Schmickl & M.M. Matschinger  28.04.2005 | 103R-16 |  |  |  | 2 | DQ313495 | 7 | DQ528967 | 29 | C |  |
| petraea | Cardaminopsis petraea (L.) Hiit. | HEID  500195 | AUT; Lower Austria; Wachau: W Mauternbach, Ferdinandswarte | 48,3803  15,5415 | M.M. Matschinger  15.04.2003 | 10M-01 |  |  |  | 2 | DQ313495 | 7 | DQ528967 | 29 | C |  |
| petraea | Cardaminopsis petraea (L.) Hiit. | HEID  500195 | AUT; Lower Austria; Wachau: W Mauternbach, Ferdinandswarte | 48,3803  15,5415 | M.M. Matschinger  15.04.2003 | 10M-02 |  |  |  | 1 | DQ313494 | 16 | DQ529013 | 1 | AC |  |
| petraea | Cardaminopsis petraea (L.) Hiit. | HEID  500195 | AUT; Lower Austria; Wachau: W Mauternbach, Ferdinandswarte | 48,3803  15,5415 | M.M. Matschinger  15.04.2003 | 10M-10 |  |  |  | 2 | DQ313495 | 22 | DQ529019 | 16 | C |  |
| petraea | Cardaminopsis petraea (L.) Hiit. | HEID  500196 | AUT; Lower Austria; Wachau: W Mauternbach, hill E Ferdinandswarte | 48,3838  15,5457 | M.M. Matschinger  15.04.2003 | 11M-01 |  |  |  | 1 | DQ313494 | 16 | DQ529013 | 1 | AC |  |
| petraea | Cardaminopsis petraea (L.) Hiit. | HEID | AUT; Lower Austria; Wachau: W Mauternbach, hill E Ferdinandswarte | 48,3838  15,5457 | M.M. Matschinger  15.04.2003 | 11M-03 |  |  |  | 2 | DQ313495 | 19 | DQ529016 | 13 | C |  |
| petraea | Cardaminopsis petraea (L.) Hiit. | HEID | AUT; Lower Austria; Wachau: W Mauternbach, hill E Ferdinandswarte | 48,3838  15,5457 | M.M. Matschinger  15.04.2003 | 11M-06 |  |  |  | 2 | DQ313495 | 7 | DQ528967 | 29 | C |  |
| petraea | Cardaminopsis petraea (L.) Hiit. | W  1969-2636 | AUT; Styria; "Totes Weib" ["Dead Woman"] Waterfall, between Lahnsattel and Frein an der Mürz | 47,7303  15,4881 | A. Polatschek | Card0455 |  |  |  | 2 | DQ313495 |  |  |  |  |  |
| petraea | Cardaminopsis petraea (L.) Hiit | LI  137550 | AUT; Styria; Grazer Bergland: Arzberg, Goller Wand | 47,2394  15,5356 | H. Melzer  19.06.1983 | Card0170 |  |  |  | 2 | DQ313495 | 63 | DQ529059 | 28 | C |  |
| petraea | Cardaminopsis hispida (Myg.) Hay. | W  1974-6951 | AUT; Styria; Grazer Bergland: NW Gollersattel, street to Arzberg | 47,2392  15,5422 | H. Melzer  20.05.1973 | Card0456 | 13 | DQ528856 | a | 2 | DQ313495 | 63 | DQ529059 |  |  |  |
| petraea | Cardaminopsis petraea (L.) Hiit. | LI  52848 | AUT; Styria; Mixnitz, Rote Wand | 47,3283  15,4042 | A. Lonsing  25.06.1933 | Card0153 |  |  |  | 2 | DQ313495 |  |  |  |  |  |
| petraea | Arabis lyrata ssp. kamchatica | DAO  185319 | CAN; Northwest Territories; Mackenzie District: 28 miles W Fort Smith, 1 mile W Seven Mile Lake | 60,0333  -112,6333 | W.J. Cody & C.C. Loan  22.07.1950 | Card0489 | 16 | DQ528878 | e | 22 | DQ313515 |  |  |  |  | no PgiC1 |
| petraea | Arabis spec. | DAO  469801 | CAN; Yukon; Beaufort Sea: west side of Clarence Lagoon | 69,6150  -140,8383 | L.C. Cwynar  21.07.1975 | Card0487 |  |  |  | 2 | DQ313495 | 22 | DQ529019 | 16 | C | no PgiC1 |
| petraea | Arabis spec. | DAO  469801 | CAN; Yukon; Beaufort Sea: west side of Clarence Lagoon | 69,6150  -140,8383 | L.C. Cwynar  21.07.1975 | Card0487-01 | 16 | DQ528878 | e |  |  |  |  |  |  | no PgiC1 |
| petraea | Arabis spec. | DAO  469801 | CAN; Yukon; Beaufort Sea: west side of Clarence Lagoon | 69,6150  -140,8383 | L.C. Cwynar  21.07.1975 | Card0487-02 | 16 | DQ528878 | e |  |  |  |  |  |  | no PgiC1 |
| petraea | Arabis lyrata ssp. kamchatica | DAO  574915 | CAN; Yukon; North Yukon National Park: British Mountains, foothills and arctic coastal lowlands, Firth River Delta | 69,5000  -139,5000 | C.E. Kennedy  26.07.1988 | Card0488 | 16 | DQ528878 | e | 2 | DQ313495 | 22 | DQ529019 | 16 | C | no PgiC1 |
| petraea | Cardaminopsis petraea (L.) Hiit. | LI  375100 | CZE; Bohemia bor.-occid.: oppidum Kadaň, pagus Suchý Důl, in locis "Sebske skaly dictis in colle nedil" | 50,3667  13,2167 | V. Zila  12.05.1991 | Card0027 | 6 | DQ528819 | b | 2 | DQ313495 | 7 | DQ528967 | 29 | C |  |
| petraea | Arabis hispida | BM | CZE; Klösterle an der Eger, Schwarzberg | 50,3933  13,1700 | J. Stelzhamer  01.06.1910 | Card0295 | 3 | DQ528816 | b | 2 | DQ313495 | 19 | DQ529016 | 13 | C |  |
| petraea | Cardaminopsis petraea | LI  289596 | CZE; Morava: Oslavany, skalky v lese na pravem brehu Oslavy pod jezem JZ od obce, roztr [rocky outcrops in forests on right bank of Oslava River] | 49,1197  16,3222 | K. Kubat  09.06.1997 | Card0082 | 2 | DQ528815 | a | 2 | DQ313495 | 7 | DQ528967 | 29 | C |  |
| petraea | Cardaminopsis petraea (L.) Hiit. | HEID  501011 | CZE; Moravský Krumlov: below St. Florian | 49,0477  16,3196 |  | 94-04 | 1 | DQ528814 | a |  |  | 71 | DQ529067 | 30 | C |  |
| petraea | Cardaminopsis petraea (L.) Hiit. | HEID  501011 | CZE; Moravský Krumlov: below St. Florian | 49,0477  16,3196 | M.M. Matschinger  11.05.2004 | 94-07 | 2 | DQ528815 | a | 2 | DQ313495 | 71 | DQ529067 | 30 | C |  |
| petraea | Cardaminopsis petraea (L.) Hiit. | HEID  501012 | CZE; SW Brünn [Brno], 3 km SW Ivanice, rocks beside street between Letkovice and Renzovice | 49,0893  16,3392 | M.M. Matschinger  11.05.2004 | 95-11 | 1 | DQ528814 | a | 2 | DQ313495 |  |  |  |  |  |
| petraea | Cardaminopsis petraea (L.) Hiit. | HEID  501013 | CZE; SW Brünn [Brno], NW Ivanice, between Nová Ves and Oslavany, above Oslava River | 49,1219  16,3244 | M.M. Matschinger  11.05.2004 | 96-05 | 2 | DQ528815 | a | 2 | DQ313495 |  |  |  |  |  |
| petraea | Arabis petraea (L.) Lam. | BM | FAI; Stromo [Streymoy] | 62,1500  -7,0000 | H.G. Vevera & F.C. Evans | Card0289 | 3 | DQ528816 | b | 17 | DQ313510 | 122 | DQ528995 | 5 | G |  |
| petraea | Arabis petraea | BM | FAI; Stromo [Streymoy] | 62,1500  -7,0000 | H.G. Vevers & F.C. Evans  11.07.1937 | LON-27 | 3 | DQ528816 | b | 17 | DQ313510 | 122 | DQ528995 | 5 | G |  |
| petraea | Cardaminopsis petraea (L.) Hiit. | HEID  501425 | GBR; Aberdeenshire; Braemar, Quoich Water, near Linn of Quoich | 57,0783  -5,2500 | M. Winkler  13.07.2003 | Card0280 | 2 | DQ528815 | a | 11 | DQ313504 | 132 | DQ529006 | 2 | AB |  |
| petraea | Cardaminopsis petraea | BM | GBR; Inverness-shire; Auchlean | 57,1000  -3,8667 | Herbarium of David S. White (1920-1990)  1949 | LON-29 | 3 | DQ528816 | b |  |  |  |  |  |  |  |
| petraea | Cardaminopsis petraea | BM | GBR; Isle of Mull; Mid Ebudes: Beinn Bheag | 56,4333  -5,7667 | B.M. Mull Survey No. 2453  28.06.1968 | LON-31 |  |  |  | 2 | DQ313495 | 122 | DQ528995 | 189 | AP |  |
| petraea | Arabis petraea (Cardaminopsis petraea) | BM | GBR; Isle of Skye; Trotternish Peninsula: The Storr | 57,5000  -6,1667 | Herbarium of Donald Peter Young (1917-1972)  17.06.1958 | LON-32 |  |  |  | 6 | DQ313499 | 142 | EU418767 | 186 | AG | no PgiC1 |
| petraea | Arabidopsis lyrata ssp. petraea | HEID  503221 | GER; Bavaria; Franconian Switzerland: SE Pottenstein, rocks W Teufelshöhle | 49,7547  11,4186 | R. Schmickl, P. Weiss & W. Schmickl | 133R-03 |  |  |  | 2 | DQ313495 | 7 | DQ528967 | 29 | C |  |
| petraea | Cardaminopsis petraea | ZT | GER; BavariaHersbruck | 49,5125  11,4339 | 20.05.1903 | 1220 |  |  |  | 2 | DQ313495 |  |  |  |  |  |
| petraea | Cardaminopsis petraea (L.) Hiit. | HEID  500361 | GER; Lower Saxony; SW-Harz: Osterode am Harz, Katzenstein | 51,7500  13,4000 | M. Koch  01.05.2000 | Ca28 | 6 | DQ528819 | b | 2 | DQ313495 | 57 | DQ529053 | 26 | AG |  |
| petraea | Cardaminopsis petraea | ZT | GER; ThuringiaS-Harz: Nordhausen, Stempeda | 51,5167  10,9167 | Behrendsen  24.09.1893 | 1212 |  |  |  | 6 | DQ313499 |  |  |  |  |  |
| petraea | Cardaminopsis petraea (L.) Hiit. | HEID  500363 | GER; Thuringia; S-Harz: Nordhausen, Stempeda, Alter Stolberg | 51,5678  10,9500 | M. Koch  01.05.2000 | Ca31 | 3 | DQ528816 | b | 2 | DQ313495 | 7 | DQ528967 | 29 | C |  |
| petraea | Cardaminopsis petraea | ZT | HUN; Zala: ad ripas lacus Balaton [Lake Balaton], in ruderatis dolomiticis declivium prope pag. Vonyare-Vashegy [Vonyarvashegy] | 46,7500  17,3167 | S. Jávorka  19.04.1927 | 1068 |  |  |  | 2 | DQ313495 |  |  |  |  |  |
| petraea | Cardaminopsis petraea (L.) Hiit. | BM | ISL; 10 km S Godafoss, up Skjalfandafljot River | 65,6833  -17,4900 | W.J.L. Sladen  06.07.1953 | Card0285 | 4 | DQ528817 | b | 2 | DQ313495 | 7 | DQ528967 | 29 | C |  |
| petraea | Cardaminopsis petraea | ZT | ISL; 45 km NE Borgarnes, Hredavatnsskali | 64,7547  -21,1042 | H.R. Preisig  29.08.1978 | 1166 |  |  |  | 6 | DQ313499 |  |  |  |  |  |
| petraea | Cardaminopsis petraea | ZT | ISL; Djúpivogur, Berufjördur | 64,6617  -14,2933 | G. Meyer  02.09.1931 | 1189 |  |  |  | 8 | DQ313501 |  |  |  |  |  |
| petraea | Cardaminopsis petraea (L.) Hiit. | BM | ISL; Hnifarver, Pjorsarver vid Hofsjökull and area W campsite | 64,5000  -18,8667 | W.J.L. Sladen  13.07.1953 | Card0284 |  |  |  | 2 | DQ313495 |  |  |  |  |  |
| petraea | Cardaminopsis petraea | BM | ISL; Hnifarver, Pjorsarver vid Hofsjökull and area W campsite | 64,5500  -18,9000 | W.J.L. Sladen  13.07.1953 | LON-01 | 3 | DQ528816 | b | 2 | DQ313495 | 7 | DQ528967 | 29 | C |  |
| petraea | Cardaminopsis petraea | ZT | ISL; Isafjördur | 66,0702  -23,1477 | G. Meyer  1933 | 1182 |  |  |  | 8 | DQ313501 |  |  |  |  |  |
| petraea | Cardaminopsis petraea (L.) Hiit. | BM | ISL; Kalmanstunga, Upper Hvita Valley | 64,7167  -21,0217 |  | Card0286 | 14 | DQ528867 | c | 2 | DQ313495 | 7 | DQ528967 | 29 | C |  |
| petraea | Cardaminopsis petraea | BM | ISL; Kalmanstunga, Upper Hvita Valley | 64,7000  -20,9167 | 01.08.1962 | LON-02 | 3 | DQ528816 | b | 2 | DQ313495 | 19 | DQ529016 | 13 | C |  |
| petraea | Cardaminopsis petraea | BM | ISL; Landmannahellir, cinder deserts | 63,9333  -19,0500 | K.A. Kershaw  18.08.1960 | LON-03 | 3 | DQ528816 | b | 32 | EU418765 | 19 | DQ529016 | 184 | AO | no PgiC1 |
| petraea | Cardaminopsis petraea (L.) Hiit. | BM | ISL; Patreksfjordur, Fjosadalur | 65,5833  -24,0000 | D.E. Patreksfjardur  01.07.1947 | Card0287 | 4 | DQ528817 | b | 6 | DQ313499 | 51 | DQ529048 | 88 | AG |  |
| petraea | Arabis petraea (L.) Lam. | BM | ISL; Reykjavík | 64,1500  -21,9500 | N. Polunin | Card0288 | 2 | DQ528815 | a | 6 | DQ313499 | 51 | DQ529048 | 88 | AG |  |
| petraea | Cardaminopsis petraea (L.) Hiit. | W  2001-14403 | ISL; Reykjavík: behind Hotel Esja | 64,1500  -21,9500 | A. Polatschek  01.07.2001 | Card0440 | 3 | DQ528816 | b | 6 | DQ313499 | 95 | DQ529091 | 106 | AG |  |
| petraea | Cardaminopsis petraea | ZT | ISL; Vestmannaeyjar | 63,4167  -20,2811 | G. Meyer  01.09.1931 | 1185 |  |  |  | 2 | DQ313495 |  |  |  |  |  |
| petraea | Cardaminopsis petraea | ZT | ISL | 65,0000  -19,0000 | G. Meyer  1931-1933 | 1190 |  |  |  | 2 | DQ313495 |  |  |  |  |  |
| petraea | Arabis petraea | BM | NOR; Bergen: Sognefjord, Lærdal | 61,1000  7,4667 | A.W. Trethwwy  04.08.1932 | Card0290 | 12 | DQ528845 | a | 6 | DQ313499 | 50 | DQ529047 | 87 | AG |  |
| petraea | Cardaminopsis petraea | 501558 | NOR; Jostedalsbreen: Briksdalen, west side of glacier | 61,6567  6,8167 | K.-G. Bernhardt  29.08.1986 | Card0102 |  |  |  | 6 | DQ313499 | 50 | DQ529047 | 87 | AG |  |
| petraea | Arabis petraea | BM | NOR; Lom: Gjendebu, i lien | 61,4500  8,5000 | H. Johnsen  26.07.1919 | Card0292 | 2 | DQ528815 | a |  |  | 50 | DQ529047 |  |  |  |
| petraea | Cardaminopsis petraea (L.) Hiit. | LI  393132 | NOR; Oppland, Jotunheimen, Visdalen, Spiterstulen | 61,6167  8,3667 | P. Schönswetter, G. Schneeweiss & A. Tribsch  28.07.1999 | Card0033 | 4 | DQ528817 | b | 9 | DQ313502 | 50 | DQ529047 | 132 | S |  |
| petraea |  |  | RUS; Karhumäki, sandy banks of Kumsa River | 62,9167  34,4167 | O. Savolainen  1999 | KAR13-03 | 111 | GQ922910 | b | 6 | DQ313499 | 184 | GQ922900 | 255 | B | no PgiC1 |
| petraea |  |  | RUS; Karhumäki, sandy banks of Kumsa River | 62,9167  34,4167 | O. Savolainen  1999 | KAR15-03 | 111 | GQ922910 | b | 6 | DQ313499 | 184 | GQ922900 | 255 | B | no PgiC1 |
| petraea |  |  | RUS; Karhumäki, sandy banks of Kumsa River | 62,9167  34,4167 | O. Savolainen  1999 | KAR01-07 | 111 | GQ922910 | b | 6 | DQ313499 | 184 | GQ922900 | 255 | B | no PgiC1 |
| petraea |  |  | RUS; Karhumäki, sandy banks of Kumsa River | 62,9167  34,4167 | O. Savolainen  1999 | KAR31 | 111 | GQ922910 | b | 6 | DQ313499 | 184 | GQ922900 | 255 | B | no PgiC1 |
| petraea | Arabidopsis petraea ssp. septentrionalis | O | RUS; Yakutia; E Lena River estuary: Karaulakh Mountains, northernmost ridges | 72,1325  126,9725 | H. Solstad & R. Elven  27.07.2004 | OSLO-11 (04/0964) | 3 | DQ528816 | b | 6 | DQ313499 | 148 | FJ477695 | 195 | AG | no PgiC1 |
| petraea | Arabidopsis petraea ssp. septentrionalis | O | RUS; Yakutia; Karaulakh Mountains: Lena River [east bank], ~18 km S Tit-Ary | 71,9250  127,3183 | H. Solstad & R. Elven  21.07.2004 | OSLO-08 (04/0589) | 3 | DQ528816 | b | 6 | DQ313499 |  |  |  |  | no PgiC1 |
| petraea | Arabidopsis petraea ssp. septentrionalis | O | RUS; Yakutia; Karaulakh Mountains: Lena River [east bank], vis-à-vis [SE] Tit-Ary | 71,9825  127,2008 | H. Solstad & R. Elven  26.07.2004 | OSLO-12 (04/0852) | 3 | DQ528816 | b | 6 | DQ313499 | 149 | FJ477696 | 196 | AG | no PgiC1 |
| petraea | Arabis septentrionalis | LE | RUS; Yakutia; Kolymskoe, Olskoe Plateau | 63,0034  160,0008 | A.P. Khokhryakov  08.09.1970 | OSLO-48 (SG-70-81) | 3 | DQ528816 | b | 6 | DQ313499 | 45 | DQ529042 | 84 | B |  |
| petraea | Arabidopsis petraea ssp. septentrionalis | O | RUS; Yakutia; Lena River [east bank]: Chekurovka, Verkhoyansk Mountains vis-à-vis Chekurovka | 71,0510  127,5834 | H. Solstad & R. Elven  17.07.2004 | OSLO-21 (04/0495) | 3 | DQ528816 | b | 33 | FJ477682 | 147 | FJ477694 | 206 | AR | no PgiC1 |
| petraea | Arabidopsis petraea ssp. septentrionalis | O | RUS; Yakutia; Lena River [west bank]: Chekurovka, surroundings of settlement | 71,0483  127,5233 | H. Solstad & R. Elven  12.07.2004 | OSLO-22 (04/0242) | 3 | DQ528816 | b | 6 | DQ313499 | 154 | FJ477701 | 201 | AG | no PgiC1 |
| petraea | Arabis petraea ssp. septentrionalis | LE | RUS; Sev.-Vost. Yakutia: Khrebet Ulakhan-Chistai | 64,9993  146,0018 | P.G. Zhukova  22.07.1975 | OSLO-54 (75-68) | 3 | DQ528816 | b | 6 | DQ313499 | 152 | FJ477699 | 199 | AG | no PgiC1 |
| petraea | Arabidopsis petraea ssp. septentrionalis | O | RUS; Yamalo-Nenetsky Autonomous Okrug; Polar Ural: ~12 km NNW Kharp, Ray-Iz Massif, eastern slope towards valley of Sob River, W Krasny Kamen railway station | 66,9000  65,6856 | I.G. Alsos & A. Tribsch  06.07.2004 | OSLO-10 (RUS-282) | 3 | DQ528816 | b | 6 | DQ313499 | 25 | DQ529022 | 192 | AG | no PgiC1 |
| petraea | Arabidopsis petraea ssp. septentrionalis | O | RUS; Yamalo-Nenetsky Autonomous Okrug; Polar Ural: ~13 km W Kharp, Ray-Iz Massif, E Mountain Chornaya [Black Mountain], Engayu Valley | 68,8408  65,5000 | I.G. Alsos & A. Tribsch  04.07.2004 | OSLO-09 (RUS-284) | 3 | DQ528816 | b | 6 | DQ313499 | 26 | DQ529023 | 193 | AG | no PgiC1 |
| petraea | Arabis umbrosa | LE | RUS; Chukotka; Anyui Mountains, Bilibino | 68,0597  166,4438 | P.G. Zhukova & V.V. Petrovsky  16.06.1966 | OSLO-39 (66-04) | 3 | DQ528816 | b | 2 | DQ313495 | 22 | DQ529019 | 16 | C | no PgiC1 |
| petraea | Arabis umbrosa | LE | RUS; Chukotka; Anyui Mountains: Kytep River | 69,5217  162,9346 | M.P. Andrejev, E.Y. Norkina & V.V. Petrovskij  17.08.1977 | OSLO-46 (SG-77-34) | 3 | DQ528816 | b | 6 | DQ313499 | 67 | DQ529063 | 194 | AG | no PgiC1 |
| petraea | Arabis umbrosa | LE | RUS; Chukotka; S-Chukotka (Yuzhno-Chukotka): Anadyrskij District, Ilmynejveem River | 65,3197  170,4461 | B.A. Yurtsev  05.08.1978 | OSLO-44 (SH-78-102) | 103 |  | ambig. b/e | 2 | DQ313495 | 88 | DQ529084 | 48 | A | no PgiC1 |
| petraea | Arabis umbrosa | LE | RUS; Chukotka; S-Chukotka: Ust-Belaya | 65,5035  173,2756 | S.A. Balandin, P.V. Petrov, B.Y. Razzhivin & B.A. Yurtsev  06.08.1974 | OSLO-43 (SG-74-204) | 106 | GQ922905 | b | 6 | DQ313499 | 50 | DQ529047 | 87 | AG | no PgiC1 |
| petraea | Arabis umbrosa | LE | RUS; Chukotka; W-Chukotka: Elveneveem River [Enmynveem River?] | 67,4408  162,3667 | B.F. Shamurin & B.A. Yurtsev  07.08.1964 | OSLO-40  (SG-64-326a) | 3 | DQ528816 | b | 2 | DQ313495 | 22 | DQ529019 | 16 | C | no PgiC1 |
| petraea | Arabis umbrosa | LE | RUS; Chukotka; W-Chukotka: Elveneveem River [Enmynveem River?] | 67,4408  162,3667 | B.F. Shamurin & B.A. Yurtsev  07.08.1964 | OSLO-41  (SG-64-326b) | 3 | DQ528816 | b |  |  | 22 | DQ529019 |  |  |  |
| petraea | Arabis umbrosa | LE | RUS; Chukotka; W-Chukotka: Elveneveem River [Enmynveem River?] | 67,4408  162,3667 | B.F. Shamurin & B.A. Yurtsev  07.08.1964 | OSLO-42  (SG-64-326c) | 3 | DQ528816 | b | 2 | DQ313495 | 22 | DQ529019 | 16 | C | no PgiC1 |
| petraea | Arabis umbrosa | LE | RUS; Chukotka; W-Chukotka: Medvezhka River, Faktoriya Medvezhka | 69,6524  162,5083 | A.A. Korodkov, V.V. Pemrovskij & I.N. Safronova  16.08.1972 | OSLO-45  (SG-72-208b) | 3 | DQ528816 | b | 2 | DQ313495 | 22 | DQ529019 | 16 | C | no PgiC1 |
| petraea | Arabis umbrosa |  | RUS; Chukotka; W-Chukotka: Medvezhka River, Faktoriya Medvezhka | 69,6524  162,5083 | A.A. Korodkov, V.V. Pemrovskij & I.N. Safronova  16.08.1972 | OSLO-50  (SG-72-208a) | 3 | DQ528816 | b | 2 | DQ313495 | 22 | DQ529019 | 16 | C | no PgiC1 |
| petraea | Arabis umbrosa | W  5174 | RUS; Oblast Magadan; Tschukotskij: Bilibinskij District, Bilibino, Bolschoj Keperveem River | 68,0833  166,5333 | T. Koroleva  29.06.1974 | WIEN-08 | 16 | DQ528878 | e | 2 | DQ313495 | 88 | DQ529084 | 48 | A | no PgiC1 |
| petraea | Arabidopsis petraea ssp. umbrosa | O | RUS; Taimyrsky Autonomous Okrug; Severo-sibirskaya Nizmennost [North Siberian Lowland]: ~1-3 km SW Khatanga, Khatanga River [left bank] and adjacent forest-tundra | 71,9631  102,9017 | P. Schönswetter & A. Tribsch  16.07.2004 | OSLO-16 (T89) | 105 | GQ922904 | e | 2 | DQ313495 | 22 | DQ529019 | 16 | C | no PgiC1 |
| petraea | Arabidopsis petraea ssp. umbrosa | O | RUS; Taimyrsky Autonomous Okrug; Severo-sibirskaya Nizmennost [North Siberian Lowland]: 50-60 km NNW Khatanga, Ary-Mas Nature Reserve, Novaya River [right bank], along river and up to ~3 km S river | 72,4644  101,8636 | P. Schönswetter & A. Tribsch  27.07.2004 | OSLO-17 (T294) | 3 | DQ528816 | b | 2 | DQ313495 | 19 | DQ529016 | 13 | C | no PgiC1 |
| petraea | Arabis umbrosa | LE | RUS; Taimyrsky Autonomous Okrug; Taimyr Peninsula: Putorana Plateau, Ozero Dyupkun [Lake Dyupkun] | 67,9375  92,1871 | 03.08.1965 | OSLO-47 (SG-65-50) | 3 | DQ528816 | b | 6 | DQ313499 | 155 | FJ477702 | 202 | AS | no PgiC1 |
| petraea | Arabidopsis petraea ssp. umbrosa | O | RUS; Wrangel Island; Somnitelnaya, Somnitelnaya River | 70,9558  179,5892 | H. Solstad & R. Elven  15.-16.08.2005 | OSLO-13  (05/0997) | 106 | GQ922905 | b | 33 | FJ477682 | 145 | FJ477692 | 204 | AR | no PgiC1 |
| petraea | Arabidopsis petraea ssp. umbrosa | O | RUS; Wrangel Island; Somnitelnaya, Somnitelnaya River | 70,9558  179,5892 | H. Solstad & R. Elven  15.-16.08.2005 | OSLO-14  (05/1007) | 106 | GQ922905 | b | 6 | DQ313499 | 150 | FJ477697 | 197 | AG | no PgiC1 |
| petraea | Arabidopsis petraea ssp. umbrosa | O | RUS; Wrangel Island; Somnitelnaya, Somnitelnaya River | 70,9558  179,5892 | H. Solstad & R. Elven  15.-16.08.2005 | OSLO-15  (05/1031) | 3 | DQ528816 | b | 6 | DQ313499 | 151 | FJ477698 | 198 | AG | no PgiC1 |
| petraea | Arabidopsis petraea ssp. umbrosa | O | RUS; Wrangel Island; Somnitelnaya, Somnitelnaya River | 70,9558  179,5892 | H. Solstad & R. Elven  15.-16.08.2005 | OSLO-24  (BE05-908-1) | 106 | GQ922905 | b | 33 | FJ477682 | 144 | FJ477691 | 203 | AR | no PgiC1 |
| petraea | Arabidopsis petraea ssp. umbrosa | O | RUS; Wrangel Island; Somnitelnaya, Somnitelnaya River | 70,9558  179,5892 | H. Solstad & R. Elven  15.-16.08.2005 | OSLO-25  (BE05-908-2) | 106 | GQ922905 | b | 33 | FJ477682 | 144 | FJ477691 | 203 | AR | no PgiC1 |
| petraea | Arabidopsis petraea ssp. umbrosa | O | RUS; Wrangel Island; Somnitelnaya, Somnitelnaya River | 70,9558  179,5892 | H. Solstad & R. Elven  15.-16.08.2005 | OSLO-26  (BE05-908-3) | 106 | GQ922905 | b | 33 | FJ477682 | 144 | FJ477691 | 203 | AR | no PgiC1 |
| petraea | Arabidopsis petraea ssp. umbrosa | O | RUS; Wrangel Island; Somnitelnaya, Somnitelnaya River | 70,9558  179,5892 | H. Solstad & R. Elven  15.-16.08.2005 | OSLO-27  (BE05-908-4) |  |  |  | 33 | FJ477682 |  |  |  |  | no PgiC1 |
| petraea | Arabidopsis petraea ssp. umbrosa | O | RUS; Wrangel Island; Somnitelnaya, Somnitelnaya River | 70,9558  179,5892 | H. Solstad & R. Elven  15.-16.08.2005 | OSLO-28  (BE05-908-5) | 106 | GQ922905 | b | 33 | FJ477682 | 144 | FJ477691 | 203 | AR | no PgiC1 |
| petraea | Arabis umbrosa | LE | RUS; Yakutia; Khrebet Ulakhan-Tas | 70,1974  134,3828 | A.A. Korodkov  07.08.1974 | OSLO-55 (SG-74-81) | 3 | DQ528816 | b | 2 | DQ313495 | 22 | DQ529019 | 16 | C | no PgiC1 |
| petraea | Arabidopsis petraea ssp. umbrosa | O | RUS; Yakutia; Lena River [east bank]: Ostrov Allakh, S Kustatim | 67,1633  123,4033 | H. Solstad & R. Elven  02.07.2004 | OSLO-19 (04/0033) | 3 | DQ528816 | b | 2 | DQ313495 | 22 | DQ529019 | 16 | C | no PgiC1 |
| petraea | Arabidopsis petraea ssp. umbrosa | O | RUS; Yakutia; Lena River [west bank]: N Siktyakh | 69,9100  125,1117 | H. Solstad & R. Elven  08.07.2004 | OSLO-20 (04/0199A) | 3 | DQ528816 | b | 6 | DQ313499 | 153 | FJ477700 | 200 | AG | no PgiC1 |
| petraea | Arabidopsis petraea ssp. umbrosa | O | RUS; Yakutia; Menkere River [south bank]: 2-3 km E outlet into Lena River | 67,9983  123,3167 | H. Solstad & R. Elven  06.07.2004 | OSLO-18 (04/0138) | 105 | GQ922904 | e | 2 | DQ313495 | 22 | DQ529019 | 16 | C | no PgiC1 |
| petraea | Arabis umbrosa | LE | RUS; Sev.-Vost. Yakutia: Khrebet Ulakhan-Chistai | 64,9993  146,0018 | A.K. Sytin, B.Y. Razzhivin & B.A. Yurtsev  20.07.1975 | OSLO-51  (75-06U) | 3 | DQ528816 | b | 33 | FJ477682 | 146 | FJ477693 | 205 | AR | no PgiC1 |
| petraea | Arabis umbrosa | LE | RUS; Sev.-Vost. Yakutia: Khrebet Ulakhan-Chistai | 64,9993  146,0018 | B.Y. Razzhivin  22.07.1975 | OSLO-56  (SG-75-233) | 111 | GQ922910 | b | 2 | DQ313495 | 22 | DQ529019 | 16 | C | no PgiC1 |
| petraea | Arabis hispida | BM | SVK; Slovensky Raj: near Puste Pole | 48,8843  20,2485 | C.M. Heard  26.06.1928 | Card0296 |  |  |  | 2 | DQ313495 | 3 | DQ528963 | 17 | A |  |
| petraea | Arabis petraea (L.) Lam. | BM | SWE; Ångermans | 60,0000  15,0000 | H. Wilh. Sjögren  01.09.1908 | Card0293 | 18 | DQ528880 | b | 6 | DQ313499 | 50 | DQ529047 | 87 | AG |  |
| petraea | Arabidopsis kamchatica |  | USA; Alaska; Atqasuk | 70,4778  -157,4135 | H. Solstad  23.08.2005 | OSLO-29  (BE05-1182-1) | 16 | DQ528878 | e | 2 | DQ313495 | 22 | DQ529019 | 16 | C | PgiC1 |
| petraea | Arabidopsis kamchatica |  | USA; Alaska; Atqasuk | 70,4778  -157,4135 | H. Solstad  23.08.2005 | OSLO-33  (BE05-1182-5) | 16 | DQ528878 | e | 2 | DQ313495 | 22 | DQ529019 | 16 | C | PgiC1 |
| petraea | Arabis lyrata ssp. kamchatica | DAO  463421 | USA; Alaska; Ikpikpuk River Quadrangle: Koluktak Test Well | 69,7667  -154,6167 | D.F. Murray & A.W. Johnson  31.07.1979 | Card0477 | 16 | DQ528878 | e | 2 | DQ313495 | 22 | DQ529019 | 16 | C | no PgiC1 |
| petraea | Arabis lyrata ssp. kamchatica | DAO  143330 | USA; Alaska; Ogotoruk Creek, ~0.5 miles from mouth | 68,1500  -165,9667 | J.P. Packer | Card0478 | 16 | DQ528878 | e | 6 | DQ313499 | 22 | DQ529019 | 190 | J |  |
| dacica | Cardaminopsis ovirensis (Wulf.) Thell. | BM | ROM; Crisana; Bihor: in herbosis montanis ad stationem "Stâna de Vale" | 46,6900  22,6150 | Al. Borza & P. Pleaneu  01.05.1936 | Card0315 | 45 | DQ528907 | o | 4 | DQ313497 | 121 | DQ528994 | 74 | E |  |
| dacica | Cardaminopsis halleri (L.) Hay. ssp. ovirensis (Wulf.) Thell. | LI  151392 | ROM; Transylvania; Muntii Harghita, Vf. Harghita | 46,5000  25,5000 | Gh. Groza  11.06.1983 | Card0259 | 29 | DQ528891 | u | 8 | DQ313501 |  |  |  |  | PgiC1 |
| dacica | Arabidopsis halleri ssp. ovirensis | SAV  RMD1 | ROM; Fǎgǎraş Mountains: Saua Caprei glacial lake | 45,6020  24,6281 | M. Kolnik  10.07.2003 | Card0353 | 23 | DQ528885 | o | 8 | DQ313501 | 42 | DQ529039 | 117 | D | PgiC1 |
| dacica | Arabidopsis halleri ssp. ovirensis | SAV  RMD1 | ROM; Fǎgǎraş Mountains: Saua Caprei glacial lake | 45,6020  24,6281 | M. Kolnik  10.07.2003 | Card0354 | 24 | DQ528886 | u | 8 | DQ313501 | 42 | DQ529039 | 117 | D | PgiC1 |
| gemmifera | Cardaminopsis halleri ssp. gemmifera | Bot.Gart.Heid. | JPN; Honshū; Kyōto | 35,0000  136,2500 |  | J020 |  |  |  | 8 | DQ313501 | 76 | DQ529072 | 144 | D | PgiC1 |
| gemmifera | Cardaminopsis halleri ssp. gemmifera | SAV | JPN; Hyōgo; Taka-gun, Nakamachi, along Omoide River | 35,0000  135,0000 | Hiroshi Kudo | OMR5 |  |  |  | 8 | DQ313501 | 75 | DQ529071 | 148 | D | PgiC1 |
| gemmifera | Cardaminopsis halleri ssp. gemmifera | SAV | JPN; Hyōgo; Taka-gun, Nakamachi, along Omoide River | 35,0000  135,0000 | Hiroshi Kudo | OMR6 | 52 | DQ528912 | s | 8 | DQ313501 | 77 | DQ529073 | 143 | D | PgiC1 |
| gemmifera | Arabidopsis halleri ssp. gemmifera | SAV | JPN; Shiga; Katsuragawa, Otsu-shi, Tira, SE foot of Mount Minako, along Ado River | 35,1917  135,8583 | J. Lihová, K. Marhold, Hiroshi Kudo & Shinji Fujii  24.04.2002 | JP18-03 | 47 | DQ528909 | r | 8 | DQ313501 | 76 | DQ529072 | 144 | D | PgiC1 |
| gemmifera | Arabidopsis halleri ssp. gemmifera | SAV | JPN; Shiga; Katsuragawa, Otsu-shi, Tira, SE foot of Mount Minako, along Ado River | 35,1917  135,8583 | J. Lihová, K. Marhold, Hiroshi Kudo & Shinji Fujii  24.04.2002 | JP18-04 | 47 | DQ528909 | r | 8 | DQ313501 | 76 | DQ529072 | 144 | D | PgiC1 |
| gemmifera | Cardaminopsis halleri ssp. gemmifera | SAV | JPN; Tochigi; Nikko-shi, Senjyugahama, Lake Chuzenji, tributaries of Sotoyamasawa River | 36,7473  139,4193 | J. Lihová, K. Marhold & Hiroshi Kudo  06.07.2003 | JP81-02 | 27 | DQ528889 | s | 8 | DQ313501 | 77 | DQ529073 | 143 | D | PgiC1 |
| gemmifera | Cardaminopsis halleri ssp. gemmifera | SAV | JPN; Tochigi; Nikko-shi, Senjyugahama, Lake Chuzenji, tributaries of Sotoyamasawa River | 36,7473  139,4193 | J. Lihová, K. Marhold & Hiroshi Kudo  06.07.2003 | JP81-03 | 28 | DQ528890 | s | 8 | DQ313501 | 77 | DQ529073 | 143 | D | PgiC1 |
| gemmifera |  | MO  1526 | ROC; Taiwan; Taichung County: Taroko National Park, Nanhu Mountain Area, Nanhutashan (Nanhu Big Mountain) | 24,3619  121,4394 | T.H. Hsieh  04.09.1995 | MO-1526 | 112 | GU647160 | r | 6 | DQ313499 |  |  |  |  |  |
| gemmifera |  |  | ROC; SE Jilin, Changbai Mountains, Mount Changbai | 42,0097  128,0556 |  | 917274-01 | 47 | DQ528909 | r | 8 | DQ313501 | 36 | FJ477685 | 257 | D | PgiC1 |
| halleri | Cardaminopsis halleri (L.) Hay. | LI  167702 | AUT; Carinthia; Lower Gail Valley: NW railway station Arnoldstein | 46,5578  13,6922 | H. Melzer  01.10.1994 | Card0246 | 20 | DQ528882 | u | 6 | DQ313499 | 61 | DQ529057 | 91 | B |  |
| halleri | Cardaminopsis cf. halleri (L.) Hay. | HEID  500352 | AUT; Lower Austria; Mariazeller Land: Ulreichsberg | 47,8356  15,4169 | M. Koch  01.05.2000 | Ca34 | 20 | DQ528882 | u | 2 | DQ313495 | 7 | DQ528967 | 29 | C | PgiC1 |
| halleri | Cardaminopsis halleri (L.) Hay. | HEID  500359 | GER; Lower Saxony; S-Harz: Herzberg, Sieber | 51,7000  10,4167 | M. Koch  01.05.2000 | Ca30 | 50 | DQ528910 | u | 8 | DQ313501 |  |  |  |  | PgiC1 |
| halleri | Cardaminopsis halleri ssp. halleri | ZT | AUT; Styria; Präbichl | 47,5167  14,9500 | E.S. Büel  26.05.1922 | 1049 |  |  |  | 6 | DQ313499 |  |  |  |  | PgiC1 |
| ovirensis | Arabidopsis halleri ssp. ovirensis | HEID | AUT; Carinthia; Mount Hochobir | 46,5061  14,4872 | M.M. Matschinger | Ovir-02 |  |  |  |  |  |  |  |  |  | no PgiC1 |
| ovirensis | Arabidopsis halleri ssp. ovirensis | HEID | AUT; Carinthia; Mount Hochobir | 46,5061  14,4872 | M.M. Matschinger | Ovir-04 |  |  |  |  |  |  |  |  |  | no PgiC1 |
| ovirensis | Arabidopsis halleri ssp. ovirensis | HEID | AUT; Carinthia; Mount Hochobir | 46,5061  14,4872 | M.M. Matschinger | Ovir-07 |  |  |  |  |  |  |  |  |  | no PgiC1 |
| ovirensis | Arabidopsis halleri ssp. ovirensis | HEID | AUT; Carinthia; Mount Hochobir | 46,5061  14,4872 | M.M. Matschinger | Ovir-10 |  |  |  |  |  |  |  |  |  | no PgiC1 |
| tatrica | Arabidopsis halleri ssp. tatrica | SAV  PLDH1 | POL; Západné Tatry: Červené vrchy, Mount Kopa Kondraczka | 49,2370  19,9313 | M. Kolnik  02.08.2003 | Card0330 | 20 | DQ528882 | u | 8 | DQ313501 | 59 | DQ529055 | 121 | D | PgiC1 |
| tatrica | Cardaminopsis halleri ssp. tatrica | SAV | SVK; Košice; Slovenský kras (Slovak Karst): Zádiel, Zádielská planina (Zadielska plain) | 48,6474  20,6520 | M. Kolnik  27.05.2003 | ZAP4 | 19 | DQ528881 | u | 8 | DQ313501 |  |  |  |  | PgiC1 |
| tatrica | Arabidopsis halleri ssp. tatrica | SAV  SBH1 | SVK; Slovensky Raj: Podlesok, PR Sucha Bela Valley | 48,9315  20,3839 | M. Kolnik  05.08.2003 | Card0372 | 19 | DQ528881 | u | 8 | DQ313501 | 59 | DQ529055 | 121 | D | PgiC1 |
| tatrica | Arabidopsis halleri ssp. tatrica | SAV  SBH2 | SVK; Slovensky Raj: Podlesok, PR Sucha Bela Valley | 48,9315  20,3839 | M. Kolnik  05.08.2003 | Card0373 | 19 | DQ528881 | u | 8 | DQ313501 | 59 | DQ529055 | 121 | D | PgiC1 |
