# Supplementary figures and images for: The evolutionary history of the Arabidopsis lyrata complex: a hybrid in the amphi-Beringian area closes a large distribution gap and builds up a genetic barrier
Source: BMC Evol Biol. 2010 Apr 8;10:98. doi: 10.1186/1471-2148-10-98 (PMC2858744; doi:10.1186/1471-2148-10-98)

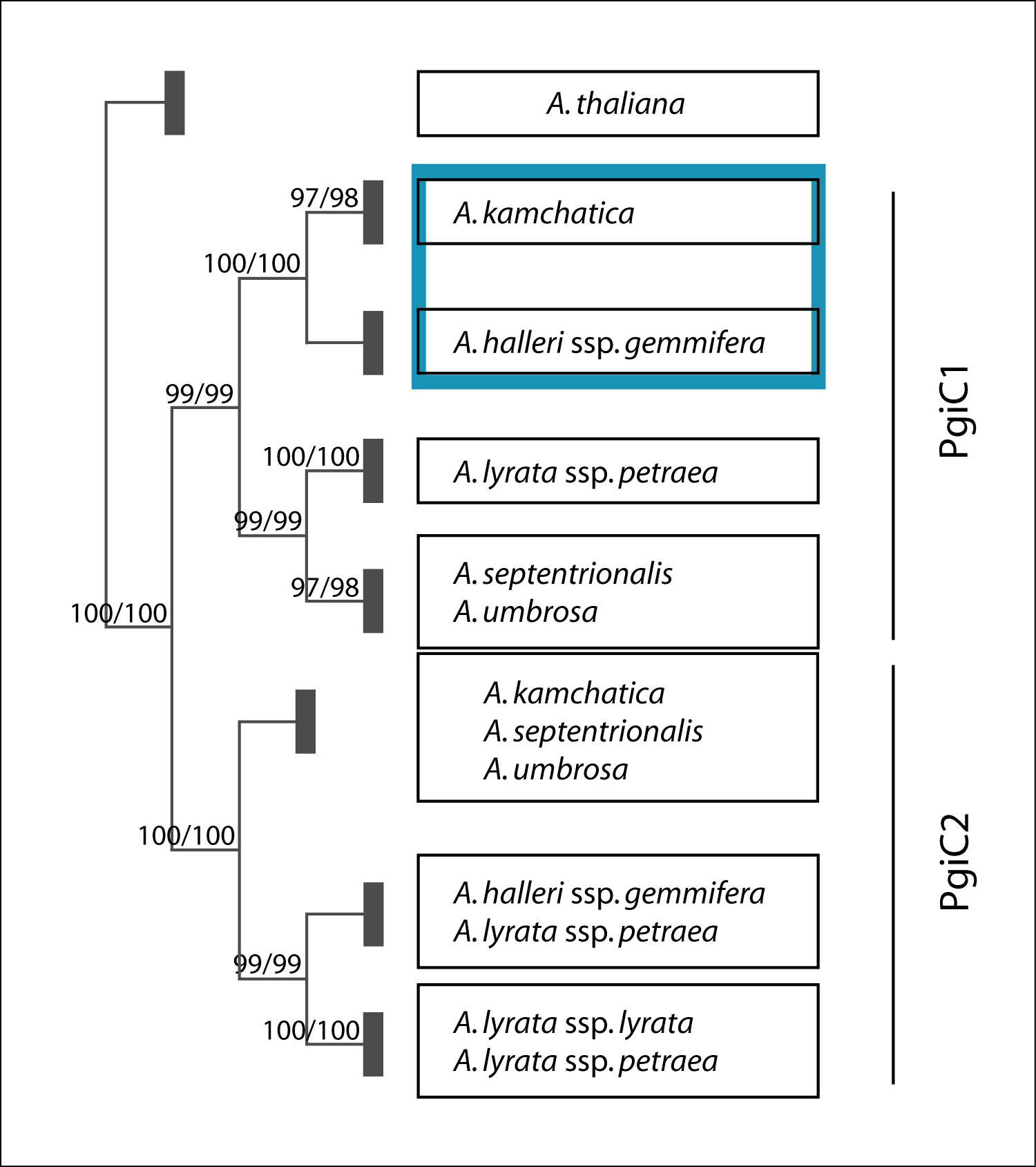

Supplement: Additional file 3 — Figure S1. Single most parsimonious tree (length = 553) with bootstrap/jackknife values above 95, based on 37 Arabidopsis nuclear DNA PgiC sequences. Heuristic searches were performed with 100 random addition sequences and TBR branch swapping, saving three trees per replicate, in TNT [102]. Gaps were treated as fifth state. Consistency index (CI) = 0.69, retention index (RI) = 0.95. Investigated accessions were from the A. lyrata complex (ssp. lyrata, ssp. petraea, A. septentrionalis, and A. umbrosa) and the A. halleri complex (ssp. gemmifera). Taxa with successful amplification of the chosen PgiC1 fragment, and, consequently, without the deletion in the forward primer site, are marked with the blue box. [file 1471-2148-10-98-S3.TIFF]

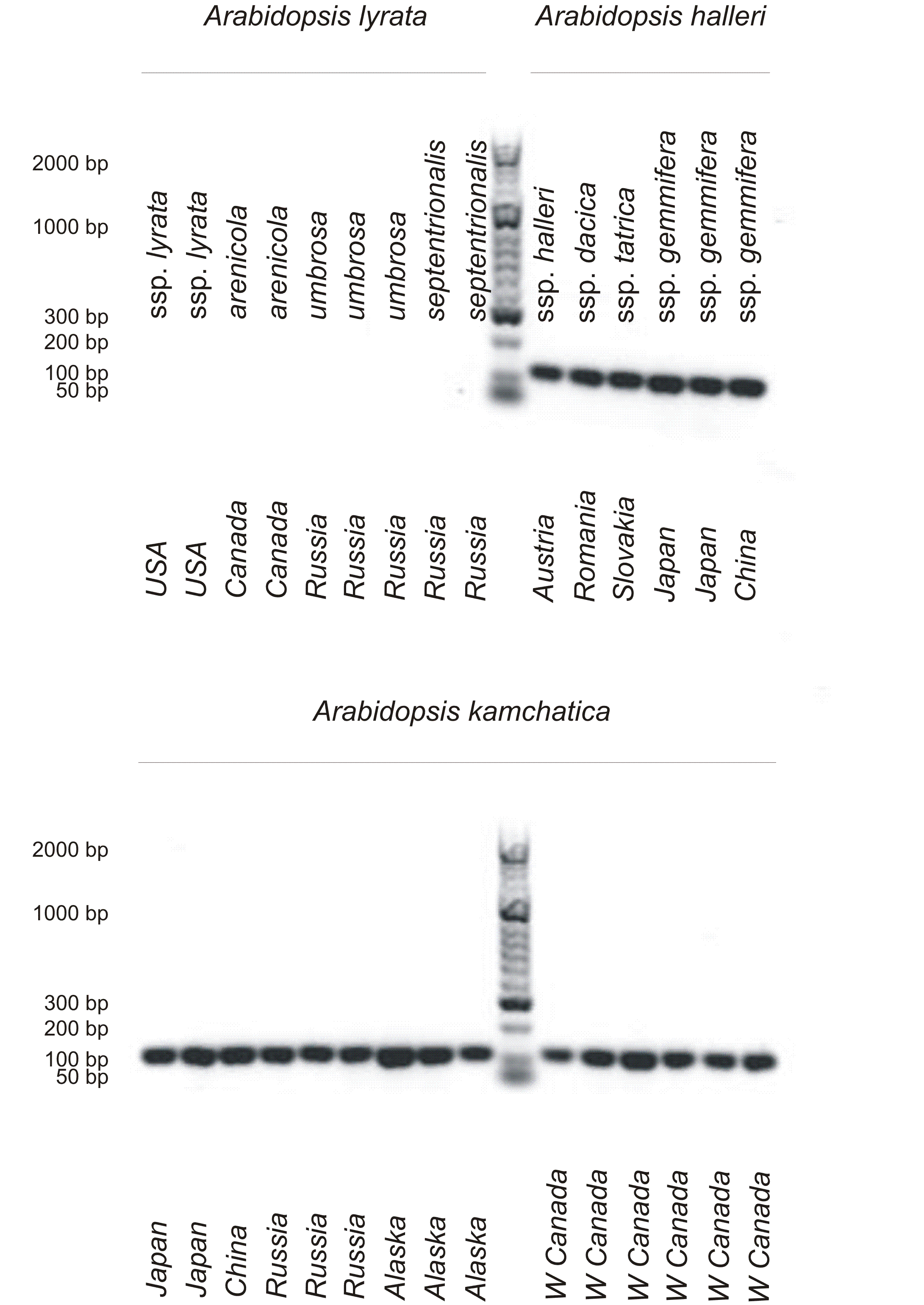

Supplement: Additional file 5 — Figure S3. Selected PCR reactions from the PgiC screening: No PgiC1 amplification in members of the Arabidopsis lyrata complex (A. lyrata ssp. lyrata, A. arenicola, Arabidopsis umbrosa, and A. septentrionalis). Successful PgiC1 amplification in members of A. halleri (ssp. halleri, ssp. dacica, ssp. tatrica, and ssp. gemmifera), and A. kamchatica. [file 1471-2148-10-98-S5.PNG]
