# Supplementary material for: The evolutionary history of the Arabidopsis lyrata complex: a hybrid in the amphi-Beringian area closes a large distribution gap and builds up a genetic barrier
Source: BMC Evol Biol. 2010 Apr 8;10:98. doi: 10.1186/1471-2148-10-98 (PMC2858744; doi:10.1186/1471-2148-10-98)
Supplement: Additional file 6 — Table S3. Taxonomic and regional genetic differentiation based on cpDNA suprahaplotypes. Numbers of cpDNA suprahaplotypes occurring in each region are provided. [file 1471-2148-10-98-S6.DOC]

**Additional file 6 - Supplementary Information Table S3.**

Taxonomic and regional genetic differentiation based on cpDNA suprahaplotypes.

| Geographic region | A | AB | AC | AD | AF | AG | AH | AI | AJ | AK | AL | AO | AP | AQ | AR | AS | AT | B | BD | BF | C | G | J | K | Q | R | S | V |
| --- | --- | --- | --- | --- | --- | --- | --- | --- | --- | --- | --- | --- | --- | --- | --- | --- | --- | --- | --- | --- | --- | --- | --- | --- | --- | --- | --- | --- |
| *A. lyrata* ssp. *petraea*:  Unglaciated  Central Europe | 7 |  | 40 |  | 2 | 1 | 1 | 5 | 1 | 1 | 2 |  |  |  |  |  |  | 8 |  |  | 120 |  | 1 | 1 | 3 | 2 |  | 1 |
| *A. lyrata* ssp. *petraea*: Glaciated  N Europe |  | 1 |  |  |  | 7 |  |  |  |  |  | 1 | 1 |  |  |  |  | 4 |  |  | 4 | 2 |  |  |  |  | 1 |  |
| *A. lyrata* ssp. *petraea*:  N Russia,  W Beringia | 2 |  |  |  |  | 11 |  |  |  |  |  |  |  |  | 7 | 1 |  | 1 |  |  | 11 |  |  |  |  |  |  |  |
| *A. lyrata* ssp. *petraea*:  E Beringia | 1 |  |  |  |  |  |  |  |  |  |  |  |  |  |  |  |  |  |  |  | 5 |  | 1 |  |  |  |  |  |
| *A. lyrata* ssp. *lyrata*: Unglaciated  N America, glaciated Great Lakes region | 42 |  |  |  |  |  |  |  |  |  |  |  |  |  |  |  |  |  | 12 | 1 |  |  |  |  |  |  |  |  |
| *A. lyrata* ssp. *arenicola*: Glaciated  N America, Greenland | 16 |  |  |  |  |  |  |  |  |  |  |  |  | 1 |  |  |  |  |  |  |  |  |  |  |  |  |  |  |
| *A. kamchatica*: Japan |  |  |  | 9 |  |  |  |  |  |  |  |  |  |  |  |  |  |  |  |  |  |  |  |  |  |  |  |  |
| *A. kamchatica*: Far Eastern Federal District, Russia |  |  |  |  |  |  |  |  |  |  |  |  |  |  |  |  |  | 9 |  |  |  |  |  |  |  |  |  |  |
| *A. kamchatica*: Alaska,  W Canada |  |  |  |  |  |  |  |  |  |  |  |  |  |  |  |  | 1 | 37 |  |  |  |  |  |  |  |  |  |  |

Numbers of cpDNA suprahaplotypes occurring in each region are provided.
