# Supplementary material for: The evolutionary history of the Arabidopsis lyrata complex: a hybrid in the amphi-Beringian area closes a large distribution gap and builds up a genetic barrier
Source: BMC Evol Biol. 2010 Apr 8;10:98. doi: 10.1186/1471-2148-10-98 (PMC2858744; doi:10.1186/1471-2148-10-98)
Supplement: Additional file 7 — Table S4. List of ITS supratypes, ITS types, trnL/F suprahaplotypes, and trnL/F haplotypes in the Arabidopsis lyrata complex with their corresponding frequencies of occurrence throughout the dataset (italic). [file 1471-2148-10-98-S7.DOC]

**Additional file 7 - Supplementary Information Table S4.**

List of ITS supratypes, ITS types, *trn*L/F suprahaplotypes, and *trn*L/F haplotypes in the *Arabidopsis lyrata* complex with their corresponding frequencies of occurrence throughout the dataset *(italic)*.

| Species | ITS supratype | ITS type | *trn*L/F suprahaplotype | *trn*L/F haplotype |
| --- | --- | --- | --- | --- |
| ***Arabidopsis arenicola*** | *1x:* **ambiguous b/e**  *2x:* **b**  *13x:* **e** | *1x:* 3, 103, 104, 107  *12x:* 16 | *1x:* **AQ**  *17x:* **A** | *1x:* 48, 181, 182, 185, 187, 188, 208  *4x:* 31  *7x:* 50 |
| ***Arabidopsis kamchatica***  **(incl. *kawasakiana*)** | *1x:* **z**  *2x:* **ambiguous b/e**  *8x:* **e**  *51x:* **b** | *1x:* 18, 108, 109, 113  *2x:* 103  *6x:* 105  *50x:* 3 | *1x:* **AT, C**  *9x:* **AD**  *46x:* **B** | *1x:* 191, 207, 253, 256  *8x:* 89  *45x:* 84 |
| ***Arabidopsis lyrata* ssp. *lyrata*** | *2x:* **y**  *35x:* e | *2x:* 110  *4x:* 109  *6x:* 108  *25x:* 16 | *1x:* **BF**  *12x:* **BD**  *41x:* **A** | *1x:* 31, 49, 249, 250  *2x:* 248, 251  *4x:* 245  *6x:* 247, 252  *10x:* 242  *20x:* 50 |
| ***Arabidopsis lyrata* ssp. *petraea*** | *1x:* **c, ambiguous b/e**  *2x:* **d**  *11x:* **e**  *36x:* **a**  *52x:* **b** | *1x:* 7, 9, 10, 11, 14, 15, 17, 18, 77, 97, 100, 103  *2x:* 5, 6, 8, 12, 105  *3x:* 4, 13  *5x:* 111  *7x:* 106  *9x:* 2, 16  *13x:* 1  *32x:* 3 | *1x:* **AB, AH, AJ, AK, AO, AP, AS, K, S, V**  *2x:* **AF, AL, G, J, R**  *3x:* **Q**  *5x:* **AI**  *7x:* **AR**  *9x:* **A**  *13x:* **B**  *19x:* **AG**  *41x:* **AC**  *142x:* **C** | *1x:* 2, 4, 17, 26, 28, 40, 43, 51, 52, 54, 78, 84, 94, 106, 132, 141, 157, 158, 159, 162, 163, 164, 165, 167, 168, 169, 170, 171, 174, 175, 176, 177, 178, 184, 186, 189, 190, 192, 193, 194, 195, 196, 197, 198, 199, 200, 201, 202, 204, 205, 206, 229, 245  *2x:* 5, 30, 48, 53, 85, 88, 151, 161, 172  *3x:* 22, 70  *4x:* 81, 87, 146, 160, 203, 255  *5x:* 166  *7x:* 152  *8x:* 11  *17x:* 16  *21x:* 13  *25x:* 1  *75x:* 29 |
